# Inventory of the Heteroptera (Insecta: Hemiptera) in Komaba Campus of the University of Tokyo, a highly urbanized area in Japan

**DOI:** 10.3897/BDJ.3.e4981

**Published:** 2015-04-24

**Authors:** Tadashi Ishikawa, Masayuki U. Saito, Keiko Kishimoto-Yamada, Toshihide Kato, Osamu Kurashima, Motomi Ito

**Affiliations:** ‡ Laboratory of Entomology, Faculty of Agriculture, Tokyo University of Agriculture, Atsugi, Kanagawa, Japan; § Department of General Systems Studies, Graduate School of Arts and Science, the University of Tokyo, Meguro, Tokyo, Japan; | Komaba Organization for Educational Excellence, the University of Tokyo, Meguro, Tokyo, Japan

**Keywords:** Arthropoda, assemblage, biodiversity information, true bugs, urban green space

## Abstract

**Background:**

The Heteroptera, or true bugs, forms one of the major insect groups with respect to the very diverse habitat preferences, including both aquatic and terrestrial species, as well as a variety of feeding types. The first comprehensive inventory of the Heteroptera at Komaba Campus of the University of Tokyo, or an urban green space in the center of the Tokyo Metropolis, Japan, was conducted.

**New information:**

A total of 115 species in 29 families of the suborder Heteroptera were identified. The area had a high species richness compared with other urbanized and suburbanized localities in Tokyo. The campus is found to show a substantial difference in heteropteran species compositions, despite being close to the other localities surrounded by highly urbanized zones in central Tokyo.

## Introduction

Although central part of the Tokyo Metropolis is a highly urbanized area, it contains several large green spaces for landscaping, such as the Imperial Palace and Meiji Jingu (Shinto Shrine), where well-preserved and managed vegetation is present (Tomokuni et al. 2000, Ishikawa and Hayashi 2013). For some of these spaces, intensive inventories on various animal groups have been conducted over the last two decades; these efforts evidently suggest substantially high species diversity even in the highly urbanized zones.

The hemipteran suborder Heteroptera (true bugs) is one of the major insect groups with respect to the habitat preferences, including aquatic, semi-aquatic, and terrestrial species with a variety of feeding types represented by varying degrees of herbivory, predation (including sucking vertebrate blood), mycophagy, and polyphagy (Schuh and Slater 1995). Due to the high habitat diversity and the relatively high environment specificity, heteropterans can be a useful bio-indicator of various environmental parameters, such as habitat structure and vegetation coverage.

The approximately 1,300 known heteropteran species of Japan are estimated to represent more than 80% of the possible total number of species (Hayashi and Miyamoto 2005, Ishikawa et al. 2012). Of these, 348 species have been recorded in the administrative districts of Tokyo from coastal plains to mountainous regions (excluding islands belonging to the metropolis), and approximately 30% of the 348 species have been found in central Tokyo (Ito et al. 2014). In faunal investigations of green spaces, 133 species have been detected at the Imperial Palace (Tomokuni et al. 2000, Tomokuni 2006) and 83 at the Meiji Jingu (Ishikawa and Hayashi 2013); these results evidently suggest that the fauna has been maintained by the diverse and well-preserved vegetation even in the highly urbanized zones. However, little is known about the fauna of relatively small green spaces, such as university campuses, parks and/or gardens.

Komaba Campus of the University of Tokyo provides the comparatively small green spaces surrounded by a highly urbanized zones in central Tokyo. Within the campus, appropriately maintained forests, shrubs and grasslands fill spaces among a number of buildings and athletic fields. Recently, two remarkable true bug species were found from broadleaf angiosperms in this campus; one was reported as a new species (Yasunaga et al. 2013) and the other as a true bug that was rediscovered after being undetected for 59 years (Ishikawa et al. 2014). Both belong to the plant bug family Miridae of the Heteroptera. These findings clearly demonstrate that further inventory surveys in such green spaces remaining in central Tokyo as Komaba Campus (apparently much smaller than the Imperial Palace or Meiji Jingu), are required. However, any comprehensive evaluation on the campus fauna or the local biodiversity of the Heteroptera is yet to be carried out.

The present paper documents the first comprehensive inventory for the heteropteran fauna in the Komaba Campus, which represents a model case of extensive research on the fauna of small green spaces in central Tokyo. We also discuss the characteristics of the heteropteran fauna on the campus in comparison with those exhibited in other urbanized or suburbanized localities in Tokyo.

## Materials and methods

### Study site

**Figure 1.**
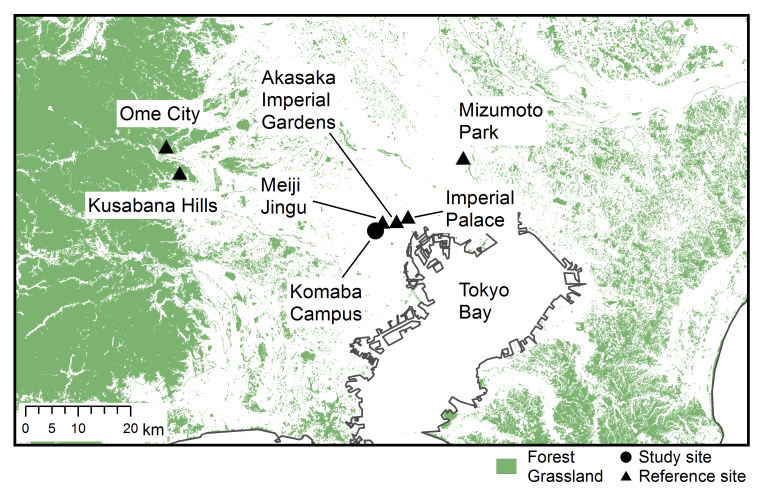
Locations of the Komaba Campus and six reference sites in Tokyo, Japan.

**Figure 2.**
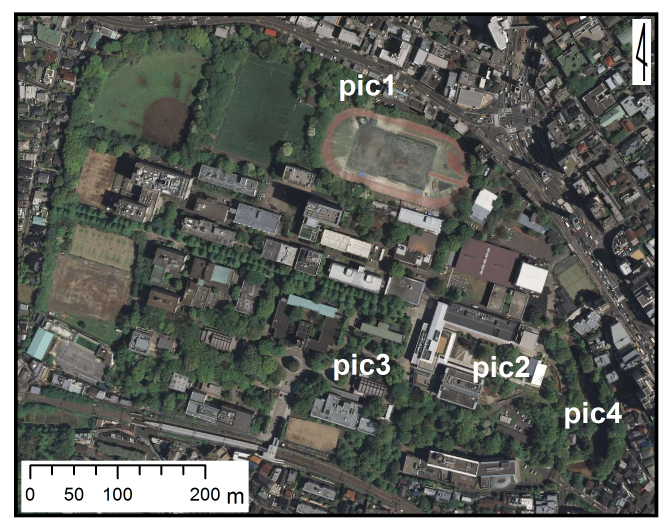
The aerial photograph of the Komaba Campus (taken in 2009 by the Geospatial Information Authority of Japan).

**Figure 3.**
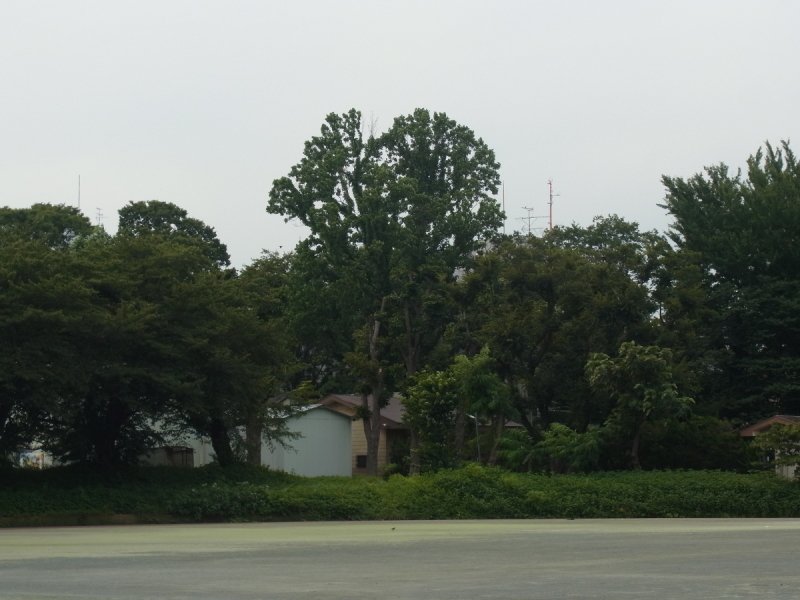
An example of a campus sampling point, indicating as "pic1" in Fig. 2.

**Figure 4.**
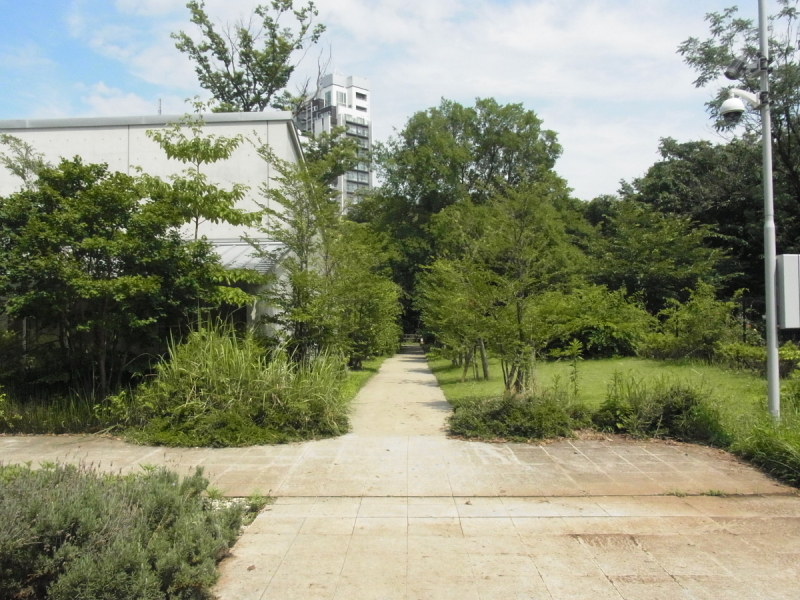
An example of a campus sampling point, indicating as "pic2" in Fig. 2.

**Figure 5.**
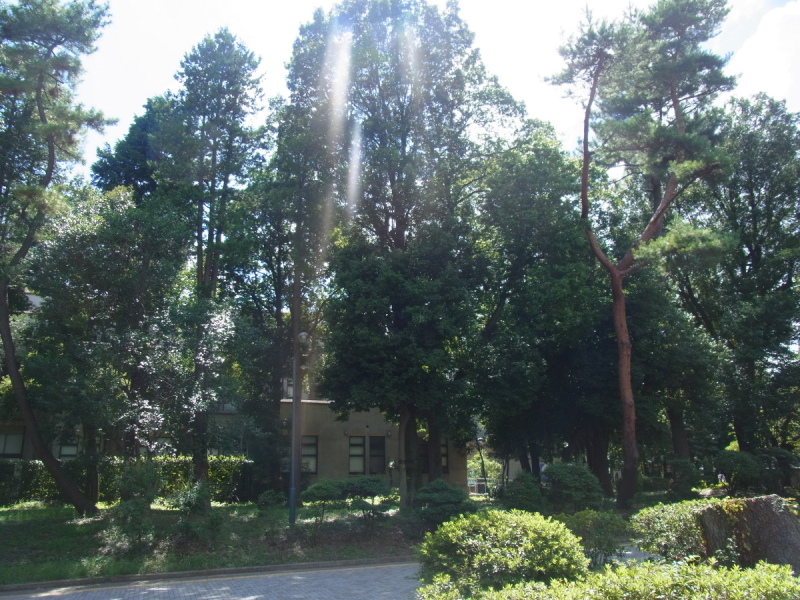
An example of a campus sampling point, indicating as "pic3" in Fig. 2.

**Figure 6.**
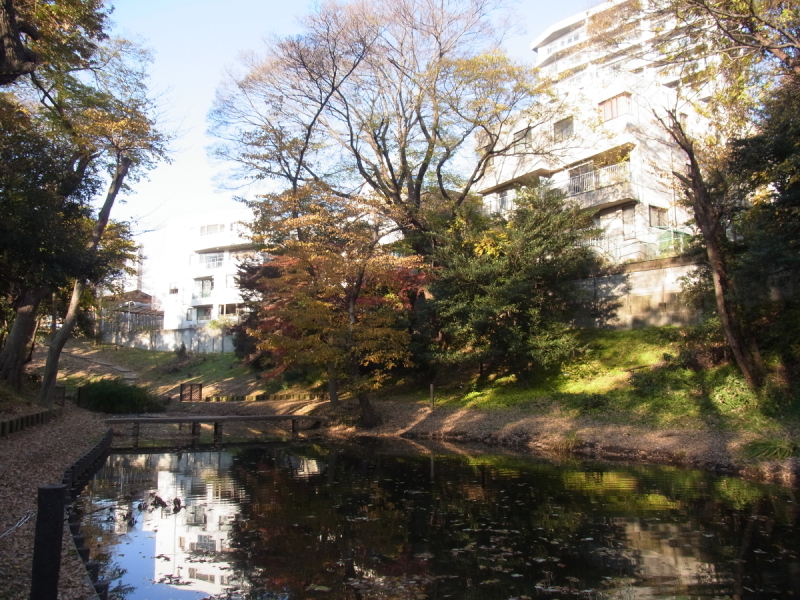
An example of a campus sampling point, indicating as "pic4" in Fig. 2.

This research was carried out at Komaba Campus (35.66006N 139.68521E; at an altitude of approximately 35 m above sea level) of the University of Tokyo, Meguro City, Tokyo, Japan, which is situated within the center of Tokyo and surrounded by highly urbanized environments including residential quarters and business complexes (Figs 1, 2​). The total site area of the campus is 25.4 ha, within which approximately 50 buildings, four athletic fields, and a few artificial pools are present. The study site was located in a warm-temperate climate zone and had an annual mean temperature of 15.4 °C and annual mean precipitation of 1,528.8 mm (Japan Meteorological Agency 2014). The vegetation is generally mosaic and characterized by various species of herbs as well as deciduous/evergreen and broadleaf/coniferous trees (Figs 2, 3, 4, 5, 6).

### Sampling methods

All specimens were collected by our research group (TI, MUS, KKY, and TK) using the following methods: net sweeping (including visual searches), UV light trap (using a high-intensity discharge lamp), and Tullgren funnels. The net sweeping method was used 41 times from April 2013 to May 2014 for a maximum of two hours per day in the daytime. The light trap method was carried out eight times from May 2013 to February 2014 for 1–1.5 hours per day shortly after sunset. Insects collected by the net sweeping and light trap methods were killed with ethyl acetate soon after capture. Sampling of the leaf litter fauna was carried out on November 28, 2013. Shortly after sampling, the leaf litter heteropterans were extracted from the sample by using Tullgren funnels, and fixed in plastic bottles filled with 60–70% ethanol. The extraction period was two days. All specimens were dried at room temperature and mounted for morphological examination.

### Identification methods

Species identification and the determination of postembryonic developmental stage and sex were performed under a stereoscopic microscope (Olympus SZ61, Tokyo, Japan) by TI, using Ishikawa et al. (2012), Hayashi and Miyamoto (2005), Yasunaga et al. (1993), and Yasunaga et al. (2001) as primary references, together with the original descriptions and/or redescriptions of each species, as necessary. For accurate identification, observations of the genitalia, if needed, were made under the stereoscopic microscope and an optical microscope (Olympus BX41) after dissection. The genitalia were preserved in small plastic tubes containing glycerin and mounted on pins with their respective specimens. All specimens examined are preserved in the Insect Collection (IC) at the Komaba Museum, the University of Tokyo, Meguro City, Tokyo, Japan (KMUT). Classification and nomenclature of taxa follow Aukema and Rieger (1995), Aukema and Rieger (1996), Aukema and Rieger (1999), Aukema and Rieger (2001), Aukema and Rieger (2006) and Aukema et al. (2012), and the family level classification within the superfamily Lygaeoidea follows Henry (1997). The arrangement of higher taxa from infraorder to family follows Ishikawa et al. (2012) and is in alphabetical order within families.

### Data analysis

The similarities in species composition (occurrence or absence) were examined using a similarity index, Jaccard distances of assemblage, for the Komaba Campus and six localities in Tokyo as reference sites (Table 1). Based on the Jaccard distances, the species compositions were compared among sites by cluster analysis with group averaging. These analyses were conducted using the ‘vegdist’ function in the ‘vegan 2.0-9’ package and ‘hclust’ function in the ‘stats 3.0.2’ package implemented in the R 3.0.2 software environment (R Core Team 2013).

## Data resources

In this study, a total of 1,541 specimens were collected and 115 species in 29 families of Heteroptera were detected on the Komaba Campus. Of these specimens, 1,451 individuals were captured by net sweeping, 75 individuals by light traps, and 15 individuals by Tullgren funnels (Table 2). Four species, *Hydrometra procera* Horváth, *Physopleurella armata* Poppius, *Botocudo japonicus* (Hidaka) and *Stigmatonotum geniculatum* (Motschulsky), were obtained using light traps only, and two species, *Stenopirates japonicus* (Esaki) and *Chilocoris confusus* Horváth by Tullgren funnels only. Five species were identified to have been introduced to Japan from abroad, *Campyloneura virgula* (Herrich-Schäffer) (Yasunaga and Yamada 2014), *Corythucha ciliata* (Say) (Tokihiro et al. 2003), *Corythucha marmorata* (Uhler) (Tomokuni 2002), *Dulinius conchatus* Distant (Tomokuni and Saito 1998), and *Leptoglossus occidentalis* Heidemann (Ishikawa and Kikuhara 2009). These alien species accounted for approximately 4.3% of all species obtained at the campus.

## Checklists

### Checklist of Heteroptera from Komaba Campus, the University of Tokyo, Tokyo, Japan

#### Enicocephalomorpha

Stichel, 1955

#### Enicocephalidae

Stål, 1860

#### Hoplitocoris lewisi

(Distant, 1903)

##### Materials

- **Type status:** Other material. **Occurrence:** recordedBy: T. Ishikawa; individualCount: 2; sex: 2 males; lifeStage: adult; otherCatalogNumbers: 2014-00001 | 2014-00002; **Taxon:** namePublishedIn: 1903; kingdom: Animalia; phylum: Arthropoda; class: Insecta; order: Hemiptera; family: Enicocephalidae; genus: Hoplitocoris; specificEpithet: lewisi; scientificNameAuthorship: Distant; **Location:** country: Japan; stateProvince: Tokyo; municipality: Meguro-ku; locality: The University of Tokyo Campus, Komaba.; minimumElevationInMeters: 31; maximumElevationInMeters: 39; decimalLatitude: 35.66006; decimalLongitude: 139.68521; geodeticDatum: WGS84; **Identification:** identifiedBy: T. Ishikawa; dateIdentified: 2013; **Event:** samplingProtocol: net sweeping; eventDate: 2013-06-16; **Record Level:** institutionCode: KMUT; collectionCode: IC
- **Type status:** Other material. **Occurrence:** recordedBy: T. Ishikawa; individualCount: 1; sex: 1 unknown; lifeStage: nymph; otherCatalogNumbers: 2014-00003; **Taxon:** namePublishedIn: 1903; kingdom: Animalia; phylum: Arthropoda; class: Insecta; order: Hemiptera; family: Enicocephalidae; genus: Hoplitocoris; specificEpithet: lewisi; scientificNameAuthorship: Distant; **Location:** country: Japan; stateProvince: Tokyo; municipality: Meguro-ku; locality: The University of Tokyo Campus, Komaba.; minimumElevationInMeters: 31; maximumElevationInMeters: 39; decimalLatitude: 35.66006; decimalLongitude: 139.68521; geodeticDatum: WGS84; **Identification:** identifiedBy: T. Ishikawa; dateIdentified: 2013; **Event:** samplingProtocol: net sweeping; eventDate: 2013-10-31; **Record Level:** institutionCode: KMUT; collectionCode: IC
- **Type status:** Other material. **Occurrence:** recordedBy: T. Ishikawa & K. Kishimoto-Yamada; individualCount: 10; sex: 10 unknowns; lifeStage: nymph; otherCatalogNumbers: 2014-00004 | 2014-00005 | 2014-00006 | 2014-00007 | 2014-00008 | 2014-00009 | 2014-00010 | 2014-00011 | 2014-00012 | 2014-00013; **Taxon:** namePublishedIn: 1903; kingdom: Animalia; phylum: Arthropoda; class: Insecta; order: Hemiptera; family: Enicocephalidae; genus: Hoplitocoris; specificEpithet: lewisi; scientificNameAuthorship: Distant; **Location:** country: Japan; stateProvince: Tokyo; municipality: Meguro-ku; locality: The University of Tokyo Campus, Komaba.; minimumElevationInMeters: 31; maximumElevationInMeters: 39; decimalLatitude: 35.66006; decimalLongitude: 139.68521; geodeticDatum: WGS84; **Identification:** identifiedBy: T. Ishikawa; dateIdentified: 2013; **Event:** samplingProtocol: Berlese funnel; eventDate: 2013-11-28; **Record Level:** institutionCode: KMUT; collectionCode: IC

#### Stenopirates japonicus

(Esaki, 1935)

##### Materials

- **Type status:** Other material. **Occurrence:** recordedBy: T. Ishikawa & K. Kishimoto-Yamada; individualCount: 1; sex: 1 unknown; lifeStage: nymph; otherCatalogNumbers: 2014-00014; **Taxon:** namePublishedIn: 1935; kingdom: Animalia; phylum: Arthropoda; class: Insecta; order: Hemiptera; family: Enicocephalidae; genus: Stenopirates; specificEpithet: japonicus; scientificNameAuthorship: Esaki; **Location:** country: Japan; stateProvince: Tokyo; municipality: Meguro-ku; locality: The University of Tokyo Campus, Komaba.; minimumElevationInMeters: 31; maximumElevationInMeters: 39; decimalLatitude: 35.66006; decimalLongitude: 139.68521; geodeticDatum: WGS84; **Identification:** identifiedBy: T. Ishikawa; dateIdentified: 2013; **Event:** samplingProtocol: Berlese funnel; eventDate: 2013-11-28; **Record Level:** institutionCode: KMUT; collectionCode: IC

#### Nepomorpha

Popov, 1968

#### Corixidae

Latreille, 1802

#### Micronecta orientalis

Wróblewski, 1960

##### Materials

- **Type status:** Other material. **Occurrence:** recordedBy: T. Ishikawa; individualCount: 1; sex: 1 female; lifeStage: adult; otherCatalogNumbers: 2014-00015; **Taxon:** namePublishedIn: 1960; kingdom: Animalia; phylum: Arthropoda; class: Insecta; order: Hemiptera; family: Corixidae; genus: Micronecta; specificEpithet: orientalis; scientificNameAuthorship: Wróblewski; **Location:** country: Japan; stateProvince: Tokyo; municipality: Meguro-ku; locality: The University of Tokyo Campus, Komaba.; minimumElevationInMeters: 31; maximumElevationInMeters: 39; decimalLatitude: 35.66006; decimalLongitude: 139.68521; geodeticDatum: WGS84; **Identification:** identifiedBy: T. Ishikawa; dateIdentified: 2013; **Event:** samplingProtocol: net sweeping; eventDate: 2013-06-14; **Record Level:** institutionCode: KMUT; collectionCode: IC

#### Notonectidae

Latreille, 1802

#### Anisops ogasawarensis

Matsumura, 1915

##### Materials

- **Type status:** Other material. **Occurrence:** recordedBy: T. Ishikawa; individualCount: 4; sex: 3 males, 1 female; lifeStage: adult; otherCatalogNumbers: 2014-00016 | 2014-00017 | 2014-00018 | 2014-00019; **Taxon:** namePublishedIn: 1915; kingdom: Animalia; phylum: Arthropoda; class: Insecta; order: Hemiptera; family: Notonectidae; genus: Anisops; specificEpithet: ogasawarensis; scientificNameAuthorship: Matsumura; **Location:** country: Japan; stateProvince: Tokyo; municipality: Meguro-ku; locality: The University of Tokyo Campus, Komaba.; minimumElevationInMeters: 31; maximumElevationInMeters: 39; decimalLatitude: 35.66006; decimalLongitude: 139.68521; geodeticDatum: WGS84; **Identification:** identifiedBy: T. Ishikawa; dateIdentified: 2013; **Event:** samplingProtocol: net sweeping; eventDate: 2013-06-22; **Record Level:** institutionCode: KMUT; collectionCode: IC
- **Type status:** Other material. **Occurrence:** recordedBy: T. Ishikawa; individualCount: 1; sex: 1 male; lifeStage: adult; otherCatalogNumbers: 2014-00020; **Taxon:** namePublishedIn: 1915; kingdom: Animalia; phylum: Arthropoda; class: Insecta; order: Hemiptera; family: Notonectidae; genus: Anisops; specificEpithet: ogasawarensis; scientificNameAuthorship: Matsumura; **Location:** country: Japan; stateProvince: Tokyo; municipality: Meguro-ku; locality: The University of Tokyo Campus, Komaba.; minimumElevationInMeters: 31; maximumElevationInMeters: 39; decimalLatitude: 35.66006; decimalLongitude: 139.68521; geodeticDatum: WGS84; **Identification:** identifiedBy: T. Ishikawa; dateIdentified: 2013; **Event:** samplingProtocol: net sweeping; eventDate: 2013-12-11; **Record Level:** institutionCode: KMUT; collectionCode: IC

#### Gerromorpha

Popov, 1971

#### Hydrometridae

Billberg, 1820

#### Hydrometra procera

Horváth, 1905

##### Materials

- **Type status:** Other material. **Occurrence:** recordedBy: T. Ishikawa; individualCount: 1; sex: 1 male; lifeStage: adult; otherCatalogNumbers: 2014-00021; **Taxon:** namePublishedIn: 1905; kingdom: Animalia; phylum: Arthropoda; class: Insecta; order: Hemiptera; family: Hydrometridae; genus: Hydrometra; specificEpithet: procera; scientificNameAuthorship: Horváth; **Location:** country: Japan; stateProvince: Tokyo; municipality: Meguro-ku; locality: The University of Tokyo Campus, Komaba.; minimumElevationInMeters: 31; maximumElevationInMeters: 39; decimalLatitude: 35.66006; decimalLongitude: 139.68521; geodeticDatum: WGS84; **Identification:** identifiedBy: T. Ishikawa; dateIdentified: 2013; **Event:** samplingProtocol: light trap; eventDate: 2013-08-02; **Record Level:** institutionCode: KMUT; collectionCode: IC

#### Veliidae

Brullé, 1836

#### Microvelia douglasi

Scott, 1874

##### Materials

- **Type status:** Other material. **Occurrence:** recordedBy: T. Ishikawa; individualCount: 28; sex: 10 males, 18 females; lifeStage: adult; otherCatalogNumbers: 2014-00022 | 2014-00023 | 2014-00024 | 2014-00025 | 2014-00026 | 2014-00027 | 2014-00028 | 2014-00029 | 2014-00030 | 2014-00031 | 2014-00032 | 2014-00033 | 2014-00034 | 2014-00035 | 2014-00036 | 2014-00037 | 2014-00038 | 2014-00039 | 2014-00040 | 2014-00041 | 2014-00042 | 2014-00043 | 2014-00044 | 2014-00045 | 2014-00046 | 2014-00047 | 2014-00048 | 2014-00049; **Taxon:** namePublishedIn: 1874; kingdom: Animalia; phylum: Arthropoda; class: Insecta; order: Hemiptera; family: Veliidae; genus: Microvelia; specificEpithet: douglasi; scientificNameAuthorship: Scott; **Location:** country: Japan; stateProvince: Tokyo; municipality: Meguro-ku; locality: The University of Tokyo Campus, Komaba.; minimumElevationInMeters: 31; maximumElevationInMeters: 39; decimalLatitude: 35.66006; decimalLongitude: 139.68521; geodeticDatum: WGS84; **Identification:** identifiedBy: T. Ishikawa; dateIdentified: 2013; **Event:** samplingProtocol: net sweeping; eventDate: 2013-06-14/2013-06-15; **Record Level:** institutionCode: KMUT; collectionCode: IC
- **Type status:** Other material. **Occurrence:** recordedBy: T. Ishikawa; individualCount: 11; sex: 7 males, 4 females; lifeStage: adult; otherCatalogNumbers: 2014-00050 | 2014-00051 | 2014-00052 | 2014-00053 | 2014-00054 | 2014-00055 | 2014-00056 | 2014-00057 | 2014-00058 | 2014-00059 | 2014-00060; **Taxon:** namePublishedIn: 1874; kingdom: Animalia; phylum: Arthropoda; class: Insecta; order: Hemiptera; family: Veliidae; genus: Microvelia; specificEpithet: douglasi; scientificNameAuthorship: Scott; **Location:** country: Japan; stateProvince: Tokyo; municipality: Meguro-ku; locality: The University of Tokyo Campus, Komaba.; minimumElevationInMeters: 31; maximumElevationInMeters: 39; decimalLatitude: 35.66006; decimalLongitude: 139.68521; geodeticDatum: WGS84; **Identification:** identifiedBy: T. Ishikawa; dateIdentified: 2013; **Event:** samplingProtocol: net sweeping; eventDate: 2013-06-22/2013-06-23; **Record Level:** institutionCode: KMUT; collectionCode: IC
- **Type status:** Other material. **Occurrence:** recordedBy: T. Ishikawa; individualCount: 58; sex: 23 males, 35 females; lifeStage: adult; otherCatalogNumbers: 2014-00061 | 2014-00062 | 2014-00063 | 2014-00064 | 2014-00065 | 2014-00066 | 2014-00067 | 2014-00068 | 2014-00069 | 2014-00070 | 2014-00071 | 2014-00072 | 2014-00073 | 2014-00074 | 2014-00075 | 2014-00076 | 2014-00077 | 2014-00078 | 2014-00079 | 2014-00080 | 2014-00081 | 2014-00082 | 2014-00083 | 2014-00084 | 2014-00085 | 2014-00086 | 2014-00087 | 2014-00088 | 2014-00089 | 2014-00090 | 2014-00091 | 2014-00092 | 2014-00093 | 2014-00094 | 2014-00095 | 2014-00096 | 2014-00097 | 2014-00098 | 2014-00099 | 2014-00100 | 2014-00101 | 2014-00102 | 2014-00103 | 2014-00104 | 2014-00105 | 2014-00106 | 2014-00107 | 2014-00108 | 2014-00109 | 2014-00110 | 2014-00111 | 2014-00112 | 2014-00113 | 2014-00114 | 2014-00115 | 2014-00116 | 2014-00117 | 2014-00118; **Taxon:** namePublishedIn: 1874; kingdom: Animalia; phylum: Arthropoda; class: Insecta; order: Hemiptera; family: Veliidae; genus: Microvelia; specificEpithet: douglasi; scientificNameAuthorship: Scott; **Location:** country: Japan; stateProvince: Tokyo; municipality: Meguro-ku; locality: The University of Tokyo Campus, Komaba.; minimumElevationInMeters: 31; maximumElevationInMeters: 39; decimalLatitude: 35.66006; decimalLongitude: 139.68521; geodeticDatum: WGS84; **Identification:** identifiedBy: T. Ishikawa; dateIdentified: 2013; **Event:** samplingProtocol: net sweeping; eventDate: 2013-06-28; **Record Level:** institutionCode: KMUT; collectionCode: IC
- **Type status:** Other material. **Occurrence:** recordedBy: T. Ishikawa; individualCount: 2; sex: 1 male, 1 female; lifeStage: adult; otherCatalogNumbers: 2014-00119 | 2014-00120; **Taxon:** namePublishedIn: 1874; kingdom: Animalia; phylum: Arthropoda; class: Insecta; order: Hemiptera; family: Veliidae; genus: Microvelia; specificEpithet: douglasi; scientificNameAuthorship: Scott; **Location:** country: Japan; stateProvince: Tokyo; municipality: Meguro-ku; locality: The University of Tokyo Campus, Komaba.; minimumElevationInMeters: 31; maximumElevationInMeters: 39; decimalLatitude: 35.66006; decimalLongitude: 139.68521; geodeticDatum: WGS84; **Identification:** identifiedBy: T. Ishikawa; dateIdentified: 2013; **Event:** samplingProtocol: net sweeping; eventDate: 2013-10-30; **Record Level:** institutionCode: KMUT; collectionCode: IC
- **Type status:** Other material. **Occurrence:** recordedBy: T. Ishikawa; individualCount: 7; sex: 6 males, 1 female; lifeStage: adult; otherCatalogNumbers: 2014-00121 | 2014-00122 | 2014-00123 | 2014-00124 | 2014-00125 | 2014-00126 | 2014-00127; **Taxon:** namePublishedIn: 1874; kingdom: Animalia; phylum: Arthropoda; class: Insecta; order: Hemiptera; family: Veliidae; genus: Microvelia; specificEpithet: douglasi; scientificNameAuthorship: Scott; **Location:** country: Japan; stateProvince: Tokyo; municipality: Meguro-ku; locality: The University of Tokyo Campus, Komaba.; minimumElevationInMeters: 31; maximumElevationInMeters: 39; decimalLatitude: 35.66006; decimalLongitude: 139.68521; geodeticDatum: WGS84; **Identification:** identifiedBy: T. Ishikawa; dateIdentified: 2013; **Event:** samplingProtocol: net sweeping; eventDate: 2013-12-11; **Record Level:** institutionCode: KMUT; collectionCode: IC

#### Microvelia horvathi

Lundblad, 1933

##### Materials

- **Type status:** Other material. **Occurrence:** recordedBy: T. Ishikawa; individualCount: 6; sex: 2 males, 4 females; lifeStage: adult; otherCatalogNumbers: 2014-00128 | 2014-00129 | 2014-00130 | 2014-00131 | 2014-00132 | 2014-00133; **Taxon:** namePublishedIn: 1933; kingdom: Animalia; phylum: Arthropoda; class: Insecta; order: Hemiptera; family: Veliidae; genus: Microvelia; specificEpithet: horvathi; scientificNameAuthorship: Lundblad; **Location:** country: Japan; stateProvince: Tokyo; municipality: Meguro-ku; locality: The University of Tokyo Campus, Komaba.; minimumElevationInMeters: 31; maximumElevationInMeters: 39; decimalLatitude: 35.66006; decimalLongitude: 139.68521; geodeticDatum: WGS84; **Identification:** identifiedBy: T. Ishikawa; dateIdentified: 2013; **Event:** samplingProtocol: net sweeping; eventDate: 2013-10-30; **Record Level:** institutionCode: KMUT; collectionCode: IC

#### Gerridae

Leach, 1815

#### Aquarius elongatus

(Uhler, 1897)

##### Materials

- **Type status:** Other material. **Occurrence:** recordedBy: T. Ishikawa; individualCount: 1; sex: 1 male; lifeStage: adult; otherCatalogNumbers: 2014-00134; **Taxon:** namePublishedIn: 1897; kingdom: Animalia; phylum: Arthropoda; class: Insecta; order: Hemiptera; family: Gerridae; genus: Aquarius; specificEpithet: elongatus; scientificNameAuthorship: Uhler; **Location:** country: Japan; stateProvince: Tokyo; municipality: Meguro-ku; locality: The University of Tokyo Campus, Komaba.; minimumElevationInMeters: 31; maximumElevationInMeters: 39; decimalLatitude: 35.66006; decimalLongitude: 139.68521; geodeticDatum: WGS84; **Identification:** identifiedBy: T. Ishikawa; dateIdentified: 2013; **Event:** samplingProtocol: net sweeping; eventDate: 2013-06-22; **Record Level:** institutionCode: KMUT; collectionCode: IC

#### Aquarius paludum paludum

(Fabricius, 1794)

##### Materials

- **Type status:** Other material. **Occurrence:** recordedBy: T. Ishikawa; individualCount: 2; sex: 2 males; lifeStage: adult; otherCatalogNumbers: 2014-00135 | 2014-00136; **Taxon:** namePublishedIn: 1794; kingdom: Animalia; phylum: Arthropoda; class: Insecta; order: Hemiptera; family: Gerridae; genus: Aquarius; specificEpithet: paludum; infraspecificEpithet: paludum; scientificNameAuthorship: Fabricius; **Location:** country: Japan; stateProvince: Tokyo; municipality: Meguro-ku; locality: The University of Tokyo Campus, Komaba.; minimumElevationInMeters: 31; maximumElevationInMeters: 39; decimalLatitude: 35.66006; decimalLongitude: 139.68521; geodeticDatum: WGS84; **Identification:** identifiedBy: T. Ishikawa; dateIdentified: 2013; **Event:** samplingProtocol: net sweeping; eventDate: 2013-06-14; **Record Level:** institutionCode: KMUT; collectionCode: IC
- **Type status:** Other material. **Occurrence:** recordedBy: T. Ishikawa; individualCount: 1; sex: 1 male; lifeStage: adult; otherCatalogNumbers: 2014-00137; **Taxon:** namePublishedIn: 1794; kingdom: Animalia; phylum: Arthropoda; class: Insecta; order: Hemiptera; family: Gerridae; genus: Aquarius; specificEpithet: paludum; infraspecificEpithet: paludum; scientificNameAuthorship: Fabricius; **Location:** country: Japan; stateProvince: Tokyo; municipality: Meguro-ku; locality: The University of Tokyo Campus, Komaba.; minimumElevationInMeters: 31; maximumElevationInMeters: 39; decimalLatitude: 35.66006; decimalLongitude: 139.68521; geodeticDatum: WGS84; **Identification:** identifiedBy: T. Ishikawa; dateIdentified: 2013; **Event:** samplingProtocol: net sweeping; eventDate: 2013-06-22; **Record Level:** institutionCode: KMUT; collectionCode: IC

#### Gerris latiabdominis

Miyamoto, 1958

##### Materials

- **Type status:** Other material. **Occurrence:** recordedBy: T. Ishikawa; individualCount: 8; sex: 3 males, 5 females; lifeStage: adult; otherCatalogNumbers: 2014-00138 | 2014-00139 | 2014-00140 | 2014-00141 | 2014-00142 | 2014-00143 | 2014-00144 | 2014-00145; **Taxon:** namePublishedIn: 1958; kingdom: Animalia; phylum: Arthropoda; class: Insecta; order: Hemiptera; family: Gerridae; genus: Gerris; specificEpithet: latiabdominis; scientificNameAuthorship: Miyamoto; **Location:** country: Japan; stateProvince: Tokyo; municipality: Meguro-ku; locality: The University of Tokyo Campus, Komaba.; minimumElevationInMeters: 31; maximumElevationInMeters: 39; decimalLatitude: 35.66006; decimalLongitude: 139.68521; geodeticDatum: WGS84; **Identification:** identifiedBy: T. Ishikawa; dateIdentified: 2013; **Event:** samplingProtocol: net sweeping; eventDate: 2013-06-14; **Record Level:** institutionCode: KMUT; collectionCode: IC
- **Type status:** Other material. **Occurrence:** recordedBy: T. Ishikawa; individualCount: 4; sex: 1 male, 3 females; lifeStage: adult; otherCatalogNumbers: 2014-00146 | 2014-00147 | 2014-00148 | 2014-00149; **Taxon:** namePublishedIn: 1958; kingdom: Animalia; phylum: Arthropoda; class: Insecta; order: Hemiptera; family: Gerridae; genus: Gerris; specificEpithet: latiabdominis; scientificNameAuthorship: Miyamoto; **Location:** country: Japan; stateProvince: Tokyo; municipality: Meguro-ku; locality: The University of Tokyo Campus, Komaba.; minimumElevationInMeters: 31; maximumElevationInMeters: 39; decimalLatitude: 35.66006; decimalLongitude: 139.68521; geodeticDatum: WGS84; **Identification:** identifiedBy: T. Ishikawa; dateIdentified: 2013; **Event:** samplingProtocol: net sweeping; eventDate: 2013-06-22; **Record Level:** institutionCode: KMUT; collectionCode: IC

#### Leptopodomorpha

Popov, 1971

#### Saldidae

Amyot et Serville, 1843

#### Saldula saltatoria

(Linnaeus, 1758)

##### Materials

- **Type status:** Other material. **Occurrence:** recordedBy: T. Ishikawa; individualCount: 1; sex: 1 male; lifeStage: adult; otherCatalogNumbers: 2014-00150; **Taxon:** namePublishedIn: 1758; kingdom: Animalia; phylum: Arthropoda; class: Insecta; order: Hemiptera; family: Saldidae; genus: Saldula; specificEpithet: saltatoria; scientificNameAuthorship: Linnaeus; **Location:** country: Japan; stateProvince: Tokyo; municipality: Meguro-ku; locality: The University of Tokyo Campus, Komaba.; minimumElevationInMeters: 31; maximumElevationInMeters: 39; decimalLatitude: 35.66006; decimalLongitude: 139.68521; geodeticDatum: WGS84; **Identification:** identifiedBy: T. Ishikawa; dateIdentified: 2013; **Event:** samplingProtocol: net sweeping; eventDate: 2013-06-22; **Record Level:** institutionCode: KMUT; collectionCode: IC

#### Cimicomorpha

Leston, Pendergrast et Southwood, 1954

#### Tingidae

Laporte, 1832

#### Corythucha ciliata

(Say, 1832)

##### Materials

- **Type status:** Other material. **Occurrence:** recordedBy: T. Ishikawa; individualCount: 2; sex: 2 males; lifeStage: adult; otherCatalogNumbers: 2014-00871 | 2014-00872; **Taxon:** namePublishedIn: 1832; kingdom: Animalia; phylum: Arthropoda; class: Insecta; order: Hemiptera; family: Tingidae; genus: Corythucha; specificEpithet: ciliata; scientificNameAuthorship: Say; **Location:** country: Japan; stateProvince: Tokyo; municipality: Meguro-ku; locality: The University of Tokyo Campus, Komaba.; minimumElevationInMeters: 31; maximumElevationInMeters: 39; decimalLatitude: 35.66006; decimalLongitude: 139.68521; geodeticDatum: WGS84; **Identification:** identifiedBy: T. Ishikawa; dateIdentified: 2013; **Event:** samplingProtocol: net sweeping; eventDate: 2013-04-28; **Record Level:** institutionCode: KMUT; collectionCode: IC
- **Type status:** Other material. **Occurrence:** recordedBy: T. Ishikawa; individualCount: 1; sex: 1 female; lifeStage: adult; otherCatalogNumbers: 2014-00873; **Taxon:** namePublishedIn: 1832; kingdom: Animalia; phylum: Arthropoda; class: Insecta; order: Hemiptera; family: Tingidae; genus: Corythucha; specificEpithet: ciliata; scientificNameAuthorship: Say; **Location:** country: Japan; stateProvince: Tokyo; municipality: Meguro-ku; locality: The University of Tokyo Campus, Komaba.; minimumElevationInMeters: 31; maximumElevationInMeters: 39; decimalLatitude: 35.66006; decimalLongitude: 139.68521; geodeticDatum: WGS84; **Identification:** identifiedBy: T. Ishikawa; dateIdentified: 2013; **Event:** samplingProtocol: net sweeping; eventDate: 2013-05-18; **Record Level:** institutionCode: KMUT; collectionCode: IC
- **Type status:** Other material. **Occurrence:** recordedBy: T. Ishikawa; individualCount: 1; sex: 1 male; lifeStage: adult; otherCatalogNumbers: 2014-00874; **Taxon:** namePublishedIn: 1832; kingdom: Animalia; phylum: Arthropoda; class: Insecta; order: Hemiptera; family: Tingidae; genus: Corythucha; specificEpithet: ciliata; scientificNameAuthorship: Say; **Location:** country: Japan; stateProvince: Tokyo; municipality: Meguro-ku; locality: The University of Tokyo Campus, Komaba.; minimumElevationInMeters: 31; maximumElevationInMeters: 39; decimalLatitude: 35.66006; decimalLongitude: 139.68521; geodeticDatum: WGS84; **Identification:** identifiedBy: T. Ishikawa; dateIdentified: 2013; **Event:** samplingProtocol: net sweeping; eventDate: 2013-08-18; **Record Level:** institutionCode: KMUT; collectionCode: IC
- **Type status:** Other material. **Occurrence:** recordedBy: T. Ishikawa; individualCount: 24; sex: 5 males, 19 females; lifeStage: adult; otherCatalogNumbers: 2014-00875 | 2014-00876 | 2014-00877 | 2014-00878 | 2014-00879 | 2014-00880 | 2014-00881 | 2014-00882 | 2014-00883 | 2014-00884 | 2014-00885 | 2014-00886 | 2014-00887 | 2014-00888 | 2014-00889 | 2014-00890 | 2014-00891 | 2014-00892 | 2014-00893 | 2014-00894 | 2014-00895 | 2014-00896 | 2014-00897 | 2014-00898; **Taxon:** namePublishedIn: 1832; kingdom: Animalia; phylum: Arthropoda; class: Insecta; order: Hemiptera; family: Tingidae; genus: Corythucha; specificEpithet: ciliata; scientificNameAuthorship: Say; **Location:** country: Japan; stateProvince: Tokyo; municipality: Meguro-ku; locality: The University of Tokyo Campus, Komaba.; minimumElevationInMeters: 31; maximumElevationInMeters: 39; decimalLatitude: 35.66006; decimalLongitude: 139.68521; geodeticDatum: WGS84; **Identification:** identifiedBy: T. Ishikawa; dateIdentified: 2014; **Event:** samplingProtocol: net sweeping; eventDate: 2014-01-01; **Record Level:** institutionCode: KMUT; collectionCode: IC

##### Notes

Known as a recent alien species to Japan (Tokihiro et al. 2003) and recorded in Tokyo for the first time by Tokyo Metropolitan Plant Protection Office (2003).

#### Corythucha marmorata

(Uhler, 1878)

##### Materials

- **Type status:** Other material. **Occurrence:** recordedBy: T. Ishikawa; individualCount: 22; sex: 8 males, 14 females; lifeStage: adult; otherCatalogNumbers: 2014-00899 | 2014-00900 | 2014-00901 | 2014-00902 | 2014-00903 | 2014-00904 | 2014-00905 | 2014-00906 | 2014-00907 | 2014-00908 | 2014-00909 | 2014-00910 | 2014-00911 | 2014-00912 | 2014-00913 | 2014-00914 | 2014-00915 | 2014-00916 | 2014-00917 | 2014-00918 | 2014-00919 | 2014-00325; **Taxon:** namePublishedIn: 1878; kingdom: Animalia; phylum: Arthropoda; class: Insecta; order: Hemiptera; family: Tingidae; genus: Corythucha; specificEpithet: marmorata; scientificNameAuthorship: Uhler; **Location:** country: Japan; stateProvince: Tokyo; municipality: Meguro-ku; locality: The University of Tokyo Campus, Komaba.; minimumElevationInMeters: 31; maximumElevationInMeters: 39; decimalLatitude: 35.66006; decimalLongitude: 139.68521; geodeticDatum: WGS84; **Identification:** identifiedBy: T. Ishikawa; dateIdentified: 2013; **Event:** samplingProtocol: net sweeping; eventDate: 2013-04-28/2013-04-29; **Record Level:** institutionCode: KMUT; collectionCode: IC
- **Type status:** Other material. **Occurrence:** recordedBy: T. Ishikawa; individualCount: 11; sex: 4 males, 7 females; lifeStage: adult; otherCatalogNumbers: 2014-00920 | 2014-00921 | 2014-00922 | 2014-00923 | 2014-00924 | 2014-00925 | 2014-00926 | 2014-00927 | 2014-00928 | 2014-00929 | 2014-00930; **Taxon:** namePublishedIn: 1878; kingdom: Animalia; phylum: Arthropoda; class: Insecta; order: Hemiptera; family: Tingidae; genus: Corythucha; specificEpithet: marmorata; scientificNameAuthorship: Uhler; **Location:** country: Japan; stateProvince: Tokyo; municipality: Meguro-ku; locality: The University of Tokyo Campus, Komaba.; minimumElevationInMeters: 31; maximumElevationInMeters: 39; decimalLatitude: 35.66006; decimalLongitude: 139.68521; geodeticDatum: WGS84; **Identification:** identifiedBy: T. Ishikawa; dateIdentified: 2013; **Event:** samplingProtocol: net sweeping; eventDate: 2013-05-12; **Record Level:** institutionCode: KMUT; collectionCode: IC
- **Type status:** Other material. **Occurrence:** recordedBy: T. Ishikawa; individualCount: 7; sex: 7 females; lifeStage: adult; otherCatalogNumbers: 2014-00931 | 2014-00932 | 2014-00933 | 2014-00934 | 2014-00935 | 2014-00936 | 2014-00937; **Taxon:** namePublishedIn: 1878; kingdom: Animalia; phylum: Arthropoda; class: Insecta; order: Hemiptera; family: Tingidae; genus: Corythucha; specificEpithet: marmorata; scientificNameAuthorship: Uhler; **Location:** country: Japan; stateProvince: Tokyo; municipality: Meguro-ku; locality: The University of Tokyo Campus, Komaba.; minimumElevationInMeters: 31; maximumElevationInMeters: 39; decimalLatitude: 35.66006; decimalLongitude: 139.68521; geodeticDatum: WGS84; **Identification:** identifiedBy: T. Ishikawa; dateIdentified: 2013; **Event:** samplingProtocol: net sweeping; eventDate: 2013-05-18; **Record Level:** institutionCode: KMUT; collectionCode: IC
- **Type status:** Other material. **Occurrence:** recordedBy: T. Ishikawa; individualCount: 2; sex: 2 females; lifeStage: adult; otherCatalogNumbers: 2014-00938 | 2014-00939; **Taxon:** namePublishedIn: 1878; kingdom: Animalia; phylum: Arthropoda; class: Insecta; order: Hemiptera; family: Tingidae; genus: Corythucha; specificEpithet: marmorata; scientificNameAuthorship: Uhler; **Location:** country: Japan; stateProvince: Tokyo; municipality: Meguro-ku; locality: The University of Tokyo Campus, Komaba.; minimumElevationInMeters: 31; maximumElevationInMeters: 39; decimalLatitude: 35.66006; decimalLongitude: 139.68521; geodeticDatum: WGS84; **Identification:** identifiedBy: T. Ishikawa; dateIdentified: 2013; **Event:** samplingProtocol: net sweeping; eventDate: 2013-05-25; **Record Level:** institutionCode: KMUT; collectionCode: IC
- **Type status:** Other material. **Occurrence:** recordedBy: T. Ishikawa; individualCount: 3; sex: 3 females; lifeStage: adult; otherCatalogNumbers: 2014-00940 | 2014-00941 | 2014-00942; **Taxon:** namePublishedIn: 1878; kingdom: Animalia; phylum: Arthropoda; class: Insecta; order: Hemiptera; family: Tingidae; genus: Corythucha; specificEpithet: marmorata; scientificNameAuthorship: Uhler; **Location:** country: Japan; stateProvince: Tokyo; municipality: Meguro-ku; locality: The University of Tokyo Campus, Komaba.; minimumElevationInMeters: 31; maximumElevationInMeters: 39; decimalLatitude: 35.66006; decimalLongitude: 139.68521; geodeticDatum: WGS84; **Identification:** identifiedBy: T. Ishikawa; dateIdentified: 2013; **Event:** samplingProtocol: net sweeping; eventDate: 2013-06-22/2013-06-23; **Record Level:** institutionCode: KMUT; collectionCode: IC

##### Notes

Known as a recent alien species to Japan (Tomokuni 2002) and recorded in Tokyo for the first time by Ishikawa and Hayashi (2013).

#### Cysteochila consueta

Drake, 1948

##### Materials

- **Type status:** Other material. **Occurrence:** recordedBy: T. Ishikawa; individualCount: 2; sex: 1 male, 1 female; lifeStage: adult; otherCatalogNumbers: 2014-00943 | 2014-00944; **Taxon:** namePublishedIn: 1948; kingdom: Animalia; phylum: Arthropoda; class: Insecta; order: Hemiptera; family: Tingidae; genus: Cysteochila; specificEpithet: consueta; scientificNameAuthorship: Drake; **Location:** country: Japan; stateProvince: Tokyo; municipality: Meguro-ku; locality: The University of Tokyo Campus, Komaba.; minimumElevationInMeters: 31; maximumElevationInMeters: 39; decimalLatitude: 35.66006; decimalLongitude: 139.68521; geodeticDatum: WGS84; **Identification:** identifiedBy: T. Ishikawa; dateIdentified: 2013; **Event:** samplingProtocol: net sweeping; eventDate: 2013-05-12; **Record Level:** institutionCode: KMUT; collectionCode: IC
- **Type status:** Other material. **Occurrence:** recordedBy: T. Ishikawa; individualCount: 25; sex: 14 males, 11 females; lifeStage: adult; otherCatalogNumbers: 2014-00945 | 2014-00946 | 2014-00947 | 2014-00948 | 2014-00949 | 2014-00950 | 2014-00951 | 2014-00952 | 2014-00953 | 2014-00954 | 2014-00955 | 2014-00956 | 2014-00957 | 2014-00958 | 2014-00959 | 2014-00960 | 2014-00961 | 2014-00962 | 2014-00963 | 2014-00964 | 2014-00965 | 2014-00966 | 2014-00967 | 2014-00968 | 2014-00969; **Taxon:** namePublishedIn: 1948; kingdom: Animalia; phylum: Arthropoda; class: Insecta; order: Hemiptera; family: Tingidae; genus: Cysteochila; specificEpithet: consueta; scientificNameAuthorship: Drake; **Location:** country: Japan; stateProvince: Tokyo; municipality: Meguro-ku; locality: The University of Tokyo Campus, Komaba.; minimumElevationInMeters: 31; maximumElevationInMeters: 39; decimalLatitude: 35.66006; decimalLongitude: 139.68521; geodeticDatum: WGS84; **Identification:** identifiedBy: T. Ishikawa; dateIdentified: 2013; **Event:** samplingProtocol: net sweeping; eventDate: 2013-05-18; **Record Level:** institutionCode: KMUT; collectionCode: IC
- **Type status:** Other material. **Occurrence:** recordedBy: T. Ishikawa; individualCount: 2; sex: 2 females; lifeStage: adult; otherCatalogNumbers: 2014-00970 | 2014-00971; **Taxon:** namePublishedIn: 1948; kingdom: Animalia; phylum: Arthropoda; class: Insecta; order: Hemiptera; family: Tingidae; genus: Cysteochila; specificEpithet: consueta; scientificNameAuthorship: Drake; **Location:** country: Japan; stateProvince: Tokyo; municipality: Meguro-ku; locality: The University of Tokyo Campus, Komaba.; minimumElevationInMeters: 31; maximumElevationInMeters: 39; decimalLatitude: 35.66006; decimalLongitude: 139.68521; geodeticDatum: WGS84; **Identification:** identifiedBy: T. Ishikawa; dateIdentified: 2013; **Event:** samplingProtocol: net sweeping; eventDate: 2013-08-15; **Record Level:** institutionCode: KMUT; collectionCode: IC
- **Type status:** Other material. **Occurrence:** recordedBy: T. Ishikawa; individualCount: 1; sex: 1 female; lifeStage: adult; otherCatalogNumbers: 2014-00972; **Taxon:** namePublishedIn: 1948; kingdom: Animalia; phylum: Arthropoda; class: Insecta; order: Hemiptera; family: Tingidae; genus: Cysteochila; specificEpithet: consueta; scientificNameAuthorship: Drake; **Location:** country: Japan; stateProvince: Tokyo; municipality: Meguro-ku; locality: The University of Tokyo Campus, Komaba.; minimumElevationInMeters: 31; maximumElevationInMeters: 39; decimalLatitude: 35.66006; decimalLongitude: 139.68521; geodeticDatum: WGS84; **Identification:** identifiedBy: T. Ishikawa; dateIdentified: 2013; **Event:** samplingProtocol: net sweeping; eventDate: 2013-08-18; **Record Level:** institutionCode: KMUT; collectionCode: IC
- **Type status:** Other material. **Occurrence:** recordedBy: T. Ishikawa; individualCount: 2; sex: 2 females; lifeStage: adult; otherCatalogNumbers: 2014-00973 | 2014-00974; **Taxon:** namePublishedIn: 1948; kingdom: Animalia; phylum: Arthropoda; class: Insecta; order: Hemiptera; family: Tingidae; genus: Cysteochila; specificEpithet: consueta; scientificNameAuthorship: Drake; **Location:** country: Japan; stateProvince: Tokyo; municipality: Meguro-ku; locality: The University of Tokyo Campus, Komaba.; minimumElevationInMeters: 31; maximumElevationInMeters: 39; decimalLatitude: 35.66006; decimalLongitude: 139.68521; geodeticDatum: WGS84; **Identification:** identifiedBy: T. Ishikawa; dateIdentified: 2013; **Event:** samplingProtocol: net sweeping; eventDate: 2013-09-06; **Record Level:** institutionCode: KMUT; collectionCode: IC
- **Type status:** Other material. **Occurrence:** recordedBy: T. Ishikawa; individualCount: 2; sex: 2 females; lifeStage: adult; otherCatalogNumbers: 2014-00975 | 2014-00976; **Taxon:** namePublishedIn: 1948; kingdom: Animalia; phylum: Arthropoda; class: Insecta; order: Hemiptera; family: Tingidae; genus: Cysteochila; specificEpithet: consueta; scientificNameAuthorship: Drake; **Location:** country: Japan; stateProvince: Tokyo; municipality: Meguro-ku; locality: The University of Tokyo Campus, Komaba.; minimumElevationInMeters: 31; maximumElevationInMeters: 39; decimalLatitude: 35.66006; decimalLongitude: 139.68521; geodeticDatum: WGS84; **Identification:** identifiedBy: T. Ishikawa; dateIdentified: 2013; **Event:** samplingProtocol: net sweeping; eventDate: 2013-10-21; **Record Level:** institutionCode: KMUT; collectionCode: IC
- **Type status:** Other material. **Occurrence:** recordedBy: T. Ishikawa; individualCount: 2; sex: 1 male, 1 female; lifeStage: adult; otherCatalogNumbers: 2014-00977 | 2014-00978; **Taxon:** namePublishedIn: 1948; kingdom: Animalia; phylum: Arthropoda; class: Insecta; order: Hemiptera; family: Tingidae; genus: Cysteochila; specificEpithet: consueta; scientificNameAuthorship: Drake; **Location:** country: Japan; stateProvince: Tokyo; municipality: Meguro-ku; locality: The University of Tokyo Campus, Komaba.; minimumElevationInMeters: 31; maximumElevationInMeters: 39; decimalLatitude: 35.66006; decimalLongitude: 139.68521; geodeticDatum: WGS84; **Identification:** identifiedBy: T. Ishikawa; dateIdentified: 2013; **Event:** samplingProtocol: net sweeping; eventDate: 2013-10-30; **Record Level:** institutionCode: KMUT; collectionCode: IC

#### Dulinius conchatus

Distant, 1903

##### Materials

- **Type status:** Other material. **Occurrence:** recordedBy: T. Ishikawa; individualCount: 2; sex: 2 females; lifeStage: adult; otherCatalogNumbers: 2014-00979 | 2014-00980; **Taxon:** namePublishedIn: 1903; kingdom: Animalia; phylum: Arthropoda; class: Insecta; order: Hemiptera; family: Tingidae; genus: Dulinius; specificEpithet: conchatus; scientificNameAuthorship: Distant; **Location:** country: Japan; stateProvince: Tokyo; municipality: Meguro-ku; locality: The University of Tokyo Campus, Komaba.; minimumElevationInMeters: 31; maximumElevationInMeters: 39; decimalLatitude: 35.66006; decimalLongitude: 139.68521; geodeticDatum: WGS84; **Identification:** identifiedBy: T. Ishikawa; dateIdentified: 2013; **Event:** samplingProtocol: net sweeping; eventDate: 2013-06-22; **Record Level:** institutionCode: KMUT; collectionCode: IC
- **Type status:** Other material. **Occurrence:** recordedBy: T. Ishikawa; individualCount: 1; sex: 1 female; lifeStage: adult; otherCatalogNumbers: 2014-00981; **Taxon:** namePublishedIn: 1903; kingdom: Animalia; phylum: Arthropoda; class: Insecta; order: Hemiptera; family: Tingidae; genus: Dulinius; specificEpithet: conchatus; scientificNameAuthorship: Distant; **Location:** country: Japan; stateProvince: Tokyo; municipality: Meguro-ku; locality: The University of Tokyo Campus, Komaba.; minimumElevationInMeters: 31; maximumElevationInMeters: 39; decimalLatitude: 35.66006; decimalLongitude: 139.68521; geodeticDatum: WGS84; **Identification:** identifiedBy: T. Ishikawa; dateIdentified: 2013; **Event:** samplingProtocol: net sweeping; eventDate: 2013-08-15; **Record Level:** institutionCode: KMUT; collectionCode: IC
- **Type status:** Other material. **Occurrence:** recordedBy: T. Ishikawa; individualCount: 7; sex: 3 males, 4 females; lifeStage: adult; otherCatalogNumbers: 2014-00982 | 2014-00983 | 2014-00984 | 2014-00985 | 2014-00986 | 2014-00987 | 2014-00988; **Taxon:** namePublishedIn: 1903; kingdom: Animalia; phylum: Arthropoda; class: Insecta; order: Hemiptera; family: Tingidae; genus: Dulinius; specificEpithet: conchatus; scientificNameAuthorship: Distant; **Location:** country: Japan; stateProvince: Tokyo; municipality: Meguro-ku; locality: The University of Tokyo Campus, Komaba.; minimumElevationInMeters: 31; maximumElevationInMeters: 39; decimalLatitude: 35.66006; decimalLongitude: 139.68521; geodeticDatum: WGS84; **Identification:** identifiedBy: T. Ishikawa; dateIdentified: 2013; **Event:** samplingProtocol: net sweeping; eventDate: 2013-08-18; **Record Level:** institutionCode: KMUT; collectionCode: IC
- **Type status:** Other material. **Occurrence:** recordedBy: T. Ishikawa; individualCount: 3; sex: 1 male, 2 females; lifeStage: adult; otherCatalogNumbers: 2014-00989 | 2014-00990 | 2014-00991; **Taxon:** namePublishedIn: 1903; kingdom: Animalia; phylum: Arthropoda; class: Insecta; order: Hemiptera; family: Tingidae; genus: Dulinius; specificEpithet: conchatus; scientificNameAuthorship: Distant; **Location:** country: Japan; stateProvince: Tokyo; municipality: Meguro-ku; locality: The University of Tokyo Campus, Komaba.; minimumElevationInMeters: 31; maximumElevationInMeters: 39; decimalLatitude: 35.66006; decimalLongitude: 139.68521; geodeticDatum: WGS84; **Identification:** identifiedBy: T. Ishikawa; dateIdentified: 2013; **Event:** samplingProtocol: net sweeping; eventDate: 2013-09-06; **Record Level:** institutionCode: KMUT; collectionCode: IC
- **Type status:** Other material. **Occurrence:** recordedBy: T. Ishikawa; individualCount: 3; sex: 1 male, 2 females; lifeStage: adult; otherCatalogNumbers: 2014-00992 | 2014-00993 | 2014-00994; **Taxon:** namePublishedIn: 1903; kingdom: Animalia; phylum: Arthropoda; class: Insecta; order: Hemiptera; family: Tingidae; genus: Dulinius; specificEpithet: conchatus; scientificNameAuthorship: Distant; **Location:** country: Japan; stateProvince: Tokyo; municipality: Meguro-ku; locality: The University of Tokyo Campus, Komaba.; minimumElevationInMeters: 31; maximumElevationInMeters: 39; decimalLatitude: 35.66006; decimalLongitude: 139.68521; geodeticDatum: WGS84; **Identification:** identifiedBy: T. Ishikawa; dateIdentified: 2013; **Event:** samplingProtocol: net sweeping; eventDate: 2013-10-30; **Record Level:** institutionCode: KMUT; collectionCode: IC

##### Notes

Known as a recent alien species to Japan (Tomokuni and Saito 1998) and recorded in Tokyo for the first time by Yamazaki (2011).

#### Stephanitis nashi

Esaki et Takeya, 1931

##### Materials

- **Type status:** Other material. **Occurrence:** recordedBy: T. Ishikawa; individualCount: 1; sex: 1 male; lifeStage: adult; otherCatalogNumbers: 2014-00995; **Taxon:** namePublishedIn: 1931; kingdom: Animalia; phylum: Arthropoda; class: Insecta; order: Hemiptera; family: Tingidae; genus: Stephanitis; specificEpithet: nashi; scientificNameAuthorship: Esaki et Takeya; **Location:** country: Japan; stateProvince: Tokyo; municipality: Meguro-ku; locality: The University of Tokyo Campus, Komaba.; minimumElevationInMeters: 31; maximumElevationInMeters: 39; decimalLatitude: 35.66006; decimalLongitude: 139.68521; geodeticDatum: WGS84; **Identification:** identifiedBy: T. Ishikawa; dateIdentified: 2013; **Event:** samplingProtocol: net sweeping; eventDate: 2013-04-29; **Record Level:** institutionCode: KMUT; collectionCode: IC
- **Type status:** Other material. **Occurrence:** recordedBy: T. Ishikawa; individualCount: 2; sex: 1 male, 1 female; lifeStage: adult; otherCatalogNumbers: 2014-00996 | 2014-00997; **Taxon:** namePublishedIn: 1931; kingdom: Animalia; phylum: Arthropoda; class: Insecta; order: Hemiptera; family: Tingidae; genus: Stephanitis; specificEpithet: nashi; scientificNameAuthorship: Esaki et Takeya; **Location:** country: Japan; stateProvince: Tokyo; municipality: Meguro-ku; locality: The University of Tokyo Campus, Komaba.; minimumElevationInMeters: 31; maximumElevationInMeters: 39; decimalLatitude: 35.66006; decimalLongitude: 139.68521; geodeticDatum: WGS84; **Identification:** identifiedBy: T. Ishikawa; dateIdentified: 2013; **Event:** samplingProtocol: net sweeping; eventDate: 2013-05-04; **Record Level:** institutionCode: KMUT; collectionCode: IC
- **Type status:** Other material. **Occurrence:** recordedBy: T. Ishikawa; individualCount: 1; sex: 1 female; lifeStage: adult; otherCatalogNumbers: 2014-00998; **Taxon:** namePublishedIn: 1931; kingdom: Animalia; phylum: Arthropoda; class: Insecta; order: Hemiptera; family: Tingidae; genus: Stephanitis; specificEpithet: nashi; scientificNameAuthorship: Esaki et Takeya; **Location:** country: Japan; stateProvince: Tokyo; municipality: Meguro-ku; locality: The University of Tokyo Campus, Komaba.; minimumElevationInMeters: 31; maximumElevationInMeters: 39; decimalLatitude: 35.66006; decimalLongitude: 139.68521; geodeticDatum: WGS84; **Identification:** identifiedBy: T. Ishikawa; dateIdentified: 2013; **Event:** samplingProtocol: net sweeping; eventDate: 2013-05-12; **Record Level:** institutionCode: KMUT; collectionCode: IC
- **Type status:** Other material. **Occurrence:** recordedBy: T. Ishikawa; individualCount: 2; sex: 1 male, 1 female; lifeStage: adult; otherCatalogNumbers: 2014-00999 | 2014-01000; **Taxon:** namePublishedIn: 1931; kingdom: Animalia; phylum: Arthropoda; class: Insecta; order: Hemiptera; family: Tingidae; genus: Stephanitis; specificEpithet: nashi; scientificNameAuthorship: Esaki et Takeya; **Location:** country: Japan; stateProvince: Tokyo; municipality: Meguro-ku; locality: The University of Tokyo Campus, Komaba.; minimumElevationInMeters: 31; maximumElevationInMeters: 39; decimalLatitude: 35.66006; decimalLongitude: 139.68521; geodeticDatum: WGS84; **Identification:** identifiedBy: T. Ishikawa; dateIdentified: 2013; **Event:** samplingProtocol: net sweeping; eventDate: 2013-05-18/2013-05-19; **Record Level:** institutionCode: KMUT; collectionCode: IC

#### Stephanitis pyrioides

(Scott, 1874)

##### Materials

- **Type status:** Other material. **Occurrence:** recordedBy: T. Ishikawa; individualCount: 2; sex: 2 males; lifeStage: adult; otherCatalogNumbers: 2014-01001 | 2014-01002; **Taxon:** namePublishedIn: 1874; kingdom: Animalia; phylum: Arthropoda; class: Insecta; order: Hemiptera; family: Tingidae; genus: Stephanitis; specificEpithet: pyrioides; scientificNameAuthorship: Scott; **Location:** country: Japan; stateProvince: Tokyo; municipality: Meguro-ku; locality: The University of Tokyo Campus, Komaba.; minimumElevationInMeters: 31; maximumElevationInMeters: 39; decimalLatitude: 35.66006; decimalLongitude: 139.68521; geodeticDatum: WGS84; **Identification:** identifiedBy: T. Ishikawa; dateIdentified: 2013; **Event:** samplingProtocol: net sweeping; eventDate: 2013-08-15; **Record Level:** institutionCode: KMUT; collectionCode: IC

#### Stephanitis svensoni

Drake, 1912

##### Materials

- **Type status:** Other material. **Occurrence:** recordedBy: T. Ishikawa; individualCount: 1; sex: 1 male; lifeStage: adult; otherCatalogNumbers: 2014-01004; **Taxon:** namePublishedIn: 1912; kingdom: Animalia; phylum: Arthropoda; class: Insecta; order: Hemiptera; family: Tingidae; genus: Stephanitis; specificEpithet: svensoni; scientificNameAuthorship: Drake; **Location:** country: Japan; stateProvince: Tokyo; municipality: Meguro-ku; locality: The University of Tokyo Campus, Komaba.; minimumElevationInMeters: 31; maximumElevationInMeters: 39; decimalLatitude: 35.66006; decimalLongitude: 139.68521; geodeticDatum: WGS84; **Identification:** identifiedBy: T. Ishikawa; dateIdentified: 2013; **Event:** samplingProtocol: net sweeping; eventDate: 2013-05-12; **Record Level:** institutionCode: KMUT; collectionCode: IC

#### Stephanitis takeyai

Drake et Maa, 1955

##### Materials

- **Type status:** Other material. **Occurrence:** recordedBy: T. Ishikawa; individualCount: 1; sex: 1 female; lifeStage: adult; otherCatalogNumbers: 2014-01003; **Taxon:** namePublishedIn: 1955; kingdom: Animalia; phylum: Arthropoda; class: Insecta; order: Hemiptera; family: Tingidae; genus: Stephanitis; specificEpithet: takeyai; scientificNameAuthorship: Drake et Maa; **Location:** country: Japan; stateProvince: Tokyo; municipality: Meguro-ku; locality: The University of Tokyo Campus, Komaba.; minimumElevationInMeters: 31; maximumElevationInMeters: 39; decimalLatitude: 35.66006; decimalLongitude: 139.68521; geodeticDatum: WGS84; **Identification:** identifiedBy: T. Ishikawa; dateIdentified: 2013; **Event:** samplingProtocol: net sweeping; eventDate: 2013-05-18; **Record Level:** institutionCode: KMUT; collectionCode: IC

#### Uhlerites debilis

(Uhler, 1896)

##### Materials

- **Type status:** Other material. **Occurrence:** recordedBy: T. Ishikawa; individualCount: 4; sex: 4 females; lifeStage: adult; otherCatalogNumbers: 2014-01005 | 2014-01006 | 2014-01007 | 2014-01008; **Taxon:** namePublishedIn: 1896; kingdom: Animalia; phylum: Arthropoda; class: Insecta; order: Hemiptera; family: Tingidae; genus: Uhlerites; specificEpithet: debilis; scientificNameAuthorship: Uhler; **Location:** country: Japan; stateProvince: Tokyo; municipality: Meguro-ku; locality: The University of Tokyo Campus, Komaba.; minimumElevationInMeters: 31; maximumElevationInMeters: 39; decimalLatitude: 35.66006; decimalLongitude: 139.68521; geodeticDatum: WGS84; **Identification:** identifiedBy: T. Ishikawa; dateIdentified: 2013; **Event:** samplingProtocol: net sweeping; eventDate: 2013-05-04; **Record Level:** institutionCode: KMUT; collectionCode: IC
- **Type status:** Other material. **Occurrence:** recordedBy: T. Ishikawa; individualCount: 1; sex: 1 female; lifeStage: adult; otherCatalogNumbers: 2014-01009; **Taxon:** namePublishedIn: 1896; kingdom: Animalia; phylum: Arthropoda; class: Insecta; order: Hemiptera; family: Tingidae; genus: Uhlerites; specificEpithet: debilis; scientificNameAuthorship: Uhler; **Location:** country: Japan; stateProvince: Tokyo; municipality: Meguro-ku; locality: The University of Tokyo Campus, Komaba.; minimumElevationInMeters: 31; maximumElevationInMeters: 39; decimalLatitude: 35.66006; decimalLongitude: 139.68521; geodeticDatum: WGS84; **Identification:** identifiedBy: T. Ishikawa; dateIdentified: 2013; **Event:** samplingProtocol: net sweeping; eventDate: 2013-05-12; **Record Level:** institutionCode: KMUT; collectionCode: IC

#### Miridae

Hahn, 1833

#### Apolygus hilaris

(Horváth, 1905)

##### Materials

- **Type status:** Other material. **Occurrence:** recordedBy: T. Ishikawa; individualCount: 1; sex: 1 male; lifeStage: adult; otherCatalogNumbers: 2014-00378; **Taxon:** namePublishedIn: 1905; kingdom: Animalia; phylum: Arthropoda; class: Insecta; order: Hemiptera; family: Miridae; genus: Apolygus; specificEpithet: hilaris; scientificNameAuthorship: Horváth; **Location:** country: Japan; stateProvince: Tokyo; municipality: Meguro-ku; locality: The University of Tokyo Campus, Komaba.; minimumElevationInMeters: 31; maximumElevationInMeters: 39; decimalLatitude: 35.66006; decimalLongitude: 139.68521; geodeticDatum: WGS84; **Identification:** identifiedBy: T. Ishikawa; dateIdentified: 2013; **Event:** samplingProtocol: net sweeping; eventDate: 2013-05-04; **Record Level:** institutionCode: KMUT; collectionCode: IC
- **Type status:** Other material. **Occurrence:** recordedBy: T. Ishikawa; individualCount: 1; sex: 1 male; lifeStage: adult; otherCatalogNumbers: 2014-00379; **Taxon:** namePublishedIn: 1905; kingdom: Animalia; phylum: Arthropoda; class: Insecta; order: Hemiptera; family: Miridae; genus: Apolygus; specificEpithet: hilaris; scientificNameAuthorship: Horváth; **Location:** country: Japan; stateProvince: Tokyo; municipality: Meguro-ku; locality: The University of Tokyo Campus, Komaba.; minimumElevationInMeters: 31; maximumElevationInMeters: 39; decimalLatitude: 35.66006; decimalLongitude: 139.68521; geodeticDatum: WGS84; **Identification:** identifiedBy: T. Ishikawa; dateIdentified: 2013; **Event:** samplingProtocol: net sweeping; eventDate: 2013-05-12; **Record Level:** institutionCode: KMUT; collectionCode: IC
- **Type status:** Other material. **Occurrence:** recordedBy: T. Ishikawa; individualCount: 3; sex: 3 females; lifeStage: adult; otherCatalogNumbers: 2014-00380 | 2014-00381 | 2014-00382; **Taxon:** namePublishedIn: 1905; kingdom: Animalia; phylum: Arthropoda; class: Insecta; order: Hemiptera; family: Miridae; genus: Apolygus; specificEpithet: hilaris; scientificNameAuthorship: Horváth; **Location:** country: Japan; stateProvince: Tokyo; municipality: Meguro-ku; locality: The University of Tokyo Campus, Komaba.; minimumElevationInMeters: 31; maximumElevationInMeters: 39; decimalLatitude: 35.66006; decimalLongitude: 139.68521; geodeticDatum: WGS84; **Identification:** identifiedBy: T. Ishikawa; dateIdentified: 2013; **Event:** samplingProtocol: net sweeping; eventDate: 2013-05-18; **Record Level:** institutionCode: KMUT; collectionCode: IC
- **Type status:** Other material. **Occurrence:** recordedBy: T. Ishikawa; individualCount: 10; sex: 5 males, 5 females; lifeStage: adult; otherCatalogNumbers: 2014-00383 | 2014-00384 | 2014-00385 | 2014-00386 | 2014-00387 | 2014-00388 | 2014-00389 | 2014-00390 | 2014-00391 | 2014-00392; **Taxon:** namePublishedIn: 1905; kingdom: Animalia; phylum: Arthropoda; class: Insecta; order: Hemiptera; family: Miridae; genus: Apolygus; specificEpithet: hilaris; scientificNameAuthorship: Horváth; **Location:** country: Japan; stateProvince: Tokyo; municipality: Meguro-ku; locality: The University of Tokyo Campus, Komaba.; minimumElevationInMeters: 31; maximumElevationInMeters: 39; decimalLatitude: 35.66006; decimalLongitude: 139.68521; geodeticDatum: WGS84; **Identification:** identifiedBy: T. Ishikawa; dateIdentified: 2013; **Event:** samplingProtocol: net sweeping; eventDate: 2013-05-25; **Record Level:** institutionCode: KMUT; collectionCode: IC
- **Type status:** Other material. **Occurrence:** recordedBy: T. Ishikawa; individualCount: 27; sex: 11 males, 16 females; lifeStage: adult; otherCatalogNumbers: 2014-00393 | 2014-00394 | 2014-00395 | 2014-00396 | 2014-00397 | 2014-00398 | 2014-00399 | 2014-00400 | 2014-00401 | 2014-00402 | 2014-00403 | 2014-00404 | 2014-00405 | 2014-00406 | 2014-00407 | 2014-00408 | 2014-00409 | 2014-00410 | 2014-00411 | 2014-00412 | 2014-00413 | 2014-00414 | 2014-00415 | 2014-00416 | 2014-00417 | 2014-00418 | 2014-00419; **Taxon:** namePublishedIn: 1905; kingdom: Animalia; phylum: Arthropoda; class: Insecta; order: Hemiptera; family: Miridae; genus: Apolygus; specificEpithet: hilaris; scientificNameAuthorship: Horváth; **Location:** country: Japan; stateProvince: Tokyo; municipality: Meguro-ku; locality: The University of Tokyo Campus, Komaba.; minimumElevationInMeters: 31; maximumElevationInMeters: 39; decimalLatitude: 35.66006; decimalLongitude: 139.68521; geodeticDatum: WGS84; **Identification:** identifiedBy: T. Ishikawa; dateIdentified: 2013; **Event:** samplingProtocol: net sweeping; eventDate: 2013-06-15/2013-06-18; **Record Level:** institutionCode: KMUT; collectionCode: IC
- **Type status:** Other material. **Occurrence:** recordedBy: T. Ishikawa; individualCount: 5; sex: 3 males, 2 females; lifeStage: adult; otherCatalogNumbers: 2014-00420 | 2014-00421 | 2014-00422 | 2014-00423 | 2014-00424; **Taxon:** namePublishedIn: 1905; kingdom: Animalia; phylum: Arthropoda; class: Insecta; order: Hemiptera; family: Miridae; genus: Apolygus; specificEpithet: hilaris; scientificNameAuthorship: Horváth; **Location:** country: Japan; stateProvince: Tokyo; municipality: Meguro-ku; locality: The University of Tokyo Campus, Komaba.; minimumElevationInMeters: 31; maximumElevationInMeters: 39; decimalLatitude: 35.66006; decimalLongitude: 139.68521; geodeticDatum: WGS84; **Identification:** identifiedBy: T. Ishikawa; dateIdentified: 2013; **Event:** samplingProtocol: net sweeping; eventDate: 2013-06-22; **Record Level:** institutionCode: KMUT; collectionCode: IC

#### Apolygus spinolae

(Meyer-Dür, 1841)

##### Materials

- **Type status:** Other material. **Occurrence:** recordedBy: T. Ishikawa; individualCount: 1; sex: 1 male; lifeStage: adult; otherCatalogNumbers: 2014-00377; **Taxon:** namePublishedIn: 1841; kingdom: Animalia; phylum: Arthropoda; class: Insecta; order: Hemiptera; family: Miridae; genus: Apolygus; specificEpithet: spinolae; scientificNameAuthorship: Meyer-Dür; **Location:** country: Japan; stateProvince: Tokyo; municipality: Meguro-ku; locality: The University of Tokyo Campus, Komaba.; minimumElevationInMeters: 31; maximumElevationInMeters: 39; decimalLatitude: 35.66006; decimalLongitude: 139.68521; geodeticDatum: WGS84; **Identification:** identifiedBy: T. Ishikawa; dateIdentified: 2013; **Event:** samplingProtocol: net sweeping; eventDate: 2013-06-23; **Record Level:** institutionCode: KMUT; collectionCode: IC

##### Notes

First record in Tokyo.

#### Apolygus subpulchellus

(Kerzhner, 1988)

##### Materials

- **Type status:** Other material. **Occurrence:** recordedBy: T. Ishikawa; individualCount: 3; sex: 3 females; lifeStage: adult; otherCatalogNumbers: 2014-00425 | 2014-00426 | 2014-00427; **Taxon:** namePublishedIn: 1988; kingdom: Animalia; phylum: Arthropoda; class: Insecta; order: Hemiptera; family: Miridae; genus: Apolygus; specificEpithet: subpulchellus; scientificNameAuthorship: Kerzhner; **Location:** country: Japan; stateProvince: Tokyo; municipality: Meguro-ku; locality: The University of Tokyo Campus, Komaba.; minimumElevationInMeters: 31; maximumElevationInMeters: 39; decimalLatitude: 35.66006; decimalLongitude: 139.68521; geodeticDatum: WGS84; **Identification:** identifiedBy: T. Ishikawa; dateIdentified: 2013; **Event:** samplingProtocol: net sweeping; eventDate: 2013-05-12; **Record Level:** institutionCode: KMUT; collectionCode: IC
- **Type status:** Other material. **Occurrence:** recordedBy: T. Ishikawa; individualCount: 39; sex: 17 males, 22 females; lifeStage: adult; otherCatalogNumbers: 2014-00428 | 2014-00429 | 2014-00430 | 2014-00431 | 2014-00432 | 2014-00433 | 2014-00434 | 2014-00435 | 2014-00436 | 2014-00437 | 2014-00438 | 2014-00439 | 2014-00440 | 2014-00441 | 2014-00442 | 2014-00443 | 2014-00444 | 2014-00445 | 2014-00446 | 2014-00447 | 2014-00448 | 2014-00449 | 2014-00450 | 2014-00451 | 2014-00452 | 2014-00453 | 2014-00454 | 2014-00455 | 2014-00456 | 2014-00457 | 2014-00458 | 2014-00459 | 2014-00460 | 2014-00461 | 2014-00462 | 2014-00463 | 2014-00464 | 2014-00465 | 2014-00466; **Taxon:** namePublishedIn: 1988; kingdom: Animalia; phylum: Arthropoda; class: Insecta; order: Hemiptera; family: Miridae; genus: Apolygus; specificEpithet: subpulchellus; scientificNameAuthorship: Kerzhner; **Location:** country: Japan; stateProvince: Tokyo; municipality: Meguro-ku; locality: The University of Tokyo Campus, Komaba.; minimumElevationInMeters: 31; maximumElevationInMeters: 39; decimalLatitude: 35.66006; decimalLongitude: 139.68521; geodeticDatum: WGS84; **Identification:** identifiedBy: T. Ishikawa; dateIdentified: 2013; **Event:** samplingProtocol: net sweeping; eventDate: 2013-05-18/2013-05-19; **Record Level:** institutionCode: KMUT; collectionCode: IC
- **Type status:** Other material. **Occurrence:** recordedBy: T. Ishikawa; individualCount: 27; sex: 11 males, 16 females; lifeStage: adult; otherCatalogNumbers: 2014-00467 | 2014-00468 | 2014-00469 | 2014-00470 | 2014-00471 | 2014-00472 | 2014-00473 | 2014-00474 | 2014-00475 | 2014-00476 | 2014-00477 | 2014-00478 | 2014-00479 | 2014-00480 | 2014-00481 | 2014-00482 | 2014-00483 | 2014-00484 | 2014-00485 | 2014-00486 | 2014-00487 | 2014-00488 | 2014-00489 | 2014-00490 | 2014-00491 | 2014-00492 | 2014-00493; **Taxon:** namePublishedIn: 1988; kingdom: Animalia; phylum: Arthropoda; class: Insecta; order: Hemiptera; family: Miridae; genus: Apolygus; specificEpithet: subpulchellus; scientificNameAuthorship: Kerzhner; **Location:** country: Japan; stateProvince: Tokyo; municipality: Meguro-ku; locality: The University of Tokyo Campus, Komaba.; minimumElevationInMeters: 31; maximumElevationInMeters: 39; decimalLatitude: 35.66006; decimalLongitude: 139.68521; geodeticDatum: WGS84; **Identification:** identifiedBy: T. Ishikawa; dateIdentified: 2013; **Event:** samplingProtocol: net sweeping; eventDate: 2013-05-25; **Record Level:** institutionCode: KMUT; collectionCode: IC
- **Type status:** Other material. **Occurrence:** recordedBy: T. Ishikawa; individualCount: 39; sex: 20 males, 19 females; lifeStage: adult; otherCatalogNumbers: 2014-00494 | 2014-00495 | 2014-00496 | 2014-00497 | 2014-00498 | 2014-00499 | 2014-00500 | 2014-00501 | 2014-00502 | 2014-00503 | 2014-00504 | 2014-00505 | 2014-00506 | 2014-00507 | 2014-00508 | 2014-00509 | 2014-00510 | 2014-00511 | 2014-00512 | 2014-00513 | 2014-00514 | 2014-00515 | 2014-00516 | 2014-00517 | 2014-00518 | 2014-00519 | 2014-00520 | 2014-00521 | 2014-00522 | 2014-00523 | 2014-00524 | 2014-00525 | 2014-00526 | 2014-00527 | 2014-00528 | 2014-00529 | 2014-00530 | 2014-00531 | 2014-00532; **Taxon:** namePublishedIn: 1988; kingdom: Animalia; phylum: Arthropoda; class: Insecta; order: Hemiptera; family: Miridae; genus: Apolygus; specificEpithet: subpulchellus; scientificNameAuthorship: Kerzhner; **Location:** country: Japan; stateProvince: Tokyo; municipality: Meguro-ku; locality: The University of Tokyo Campus, Komaba.; minimumElevationInMeters: 31; maximumElevationInMeters: 39; decimalLatitude: 35.66006; decimalLongitude: 139.68521; geodeticDatum: WGS84; **Identification:** identifiedBy: T. Ishikawa; dateIdentified: 2013; **Event:** samplingProtocol: net sweeping; eventDate: 2013-06-15/2013-06-18; **Record Level:** institutionCode: KMUT; collectionCode: IC
- **Type status:** Other material. **Occurrence:** recordedBy: T. Ishikawa; individualCount: 26; sex: 8 males, 18 females; lifeStage: adult; otherCatalogNumbers: 2014-00533 | 2014-00534 | 2014-00535 | 2014-00536 | 2014-00537 | 2014-00538 | 2014-00539 | 2014-00540 | 2014-00541 | 2014-00542 | 2014-00543 | 2014-00544 | 2014-00545 | 2014-00546 | 2014-00547 | 2014-00548 | 2014-00549 | 2014-00550 | 2014-00551 | 2014-00552 | 2014-00553 | 2014-00554 | 2014-00555 | 2014-00556 | 2014-00557 | 2014-00558; **Taxon:** namePublishedIn: 1988; kingdom: Animalia; phylum: Arthropoda; class: Insecta; order: Hemiptera; family: Miridae; genus: Apolygus; specificEpithet: subpulchellus; scientificNameAuthorship: Kerzhner; **Location:** country: Japan; stateProvince: Tokyo; municipality: Meguro-ku; locality: The University of Tokyo Campus, Komaba.; minimumElevationInMeters: 31; maximumElevationInMeters: 39; decimalLatitude: 35.66006; decimalLongitude: 139.68521; geodeticDatum: WGS84; **Identification:** identifiedBy: T. Ishikawa; dateIdentified: 2013; **Event:** samplingProtocol: net sweeping; eventDate: 2013-06-22/2013-06-26; **Record Level:** institutionCode: KMUT; collectionCode: IC

#### Atractotomoidea castanea

Yasunaga, 1999

##### Materials

- **Type status:** Other material. **Occurrence:** recordedBy: T. Ishikawa; individualCount: 1; sex: 1 male; lifeStage: adult; otherCatalogNumbers: 2014-00195; **Taxon:** namePublishedIn: 1999; kingdom: Animalia; phylum: Arthropoda; class: Insecta; order: Hemiptera; family: Miridae; genus: Atractotomoidea; specificEpithet: castanea; scientificNameAuthorship: Yasunaga; **Location:** country: Japan; stateProvince: Tokyo; municipality: Meguro-ku; locality: The University of Tokyo Campus, Komaba.; minimumElevationInMeters: 31; maximumElevationInMeters: 39; decimalLatitude: 35.66006; decimalLongitude: 139.68521; geodeticDatum: WGS84; **Identification:** identifiedBy: T. Ishikawa; dateIdentified: 2013; **Event:** samplingProtocol: net sweeping; eventDate: 2013-06-23; **Record Level:** institutionCode: KMUT; collectionCode: IC

##### Notes

First record in Tokyo.

#### Campylomma lividum

Reuter, 1885

##### Materials

- **Type status:** Other material. **Occurrence:** recordedBy: T. Ishikawa; individualCount: 3; sex: 1 male, 2 females; lifeStage: adult; otherCatalogNumbers: 2014-00196 | 2014-00197 | 2014-00198; **Taxon:** namePublishedIn: 1885; kingdom: Animalia; phylum: Arthropoda; class: Insecta; order: Hemiptera; family: Miridae; genus: Campylomma; specificEpithet: lividum; scientificNameAuthorship: Reuter; **Location:** country: Japan; stateProvince: Tokyo; municipality: Meguro-ku; locality: The University of Tokyo Campus, Komaba.; minimumElevationInMeters: 31; maximumElevationInMeters: 39; decimalLatitude: 35.66006; decimalLongitude: 139.68521; geodeticDatum: WGS84; **Identification:** identifiedBy: T. Ishikawa; dateIdentified: 2013; **Event:** samplingProtocol: net sweeping; eventDate: 2013-09-06; **Record Level:** institutionCode: KMUT; collectionCode: IC
- **Type status:** Other material. **Occurrence:** recordedBy: T. Ishikawa; individualCount: 2; sex: 1 male, 1 female; lifeStage: adult; otherCatalogNumbers: 2014-00199 | 2014-00200; **Taxon:** namePublishedIn: 1885; kingdom: Animalia; phylum: Arthropoda; class: Insecta; order: Hemiptera; family: Miridae; genus: Campylomma; specificEpithet: lividum; scientificNameAuthorship: Reuter; **Location:** country: Japan; stateProvince: Tokyo; municipality: Meguro-ku; locality: The University of Tokyo Campus, Komaba.; minimumElevationInMeters: 31; maximumElevationInMeters: 39; decimalLatitude: 35.66006; decimalLongitude: 139.68521; geodeticDatum: WGS84; **Identification:** identifiedBy: T. Ishikawa; dateIdentified: 2013; **Event:** samplingProtocol: net sweeping; eventDate: 2013-10-21; **Record Level:** institutionCode: KMUT; collectionCode: IC
- **Type status:** Other material. **Occurrence:** recordedBy: T. Ishikawa; individualCount: 1; sex: 1 male; lifeStage: adult; otherCatalogNumbers: 2014-00201; **Taxon:** namePublishedIn: 1885; kingdom: Animalia; phylum: Arthropoda; class: Insecta; order: Hemiptera; family: Miridae; genus: Campylomma; specificEpithet: lividum; scientificNameAuthorship: Reuter; **Location:** country: Japan; stateProvince: Tokyo; municipality: Meguro-ku; locality: The University of Tokyo Campus, Komaba.; minimumElevationInMeters: 31; maximumElevationInMeters: 39; decimalLatitude: 35.66006; decimalLongitude: 139.68521; geodeticDatum: WGS84; **Identification:** identifiedBy: T. Ishikawa; dateIdentified: 2013; **Event:** samplingProtocol: net sweeping; eventDate: 2013-10-30; **Record Level:** institutionCode: KMUT; collectionCode: IC

##### Notes

So far known as “*Campylomma chinense* Schuh, 1984” in Japan (Yasunaga and Duwal 2014).

#### Campyloneura virgula

(Herrich-Schaeffer, 1836)

##### Materials

- **Type status:** Other material. **Occurrence:** recordedBy: T. Ishikawa; individualCount: 8; sex: 8 females; lifeStage: adult; otherCatalogNumbers: 2014-01533 | 2014-01534 | 2014-01535 | 2014-01536 | 2014-01537 | 2014-01538 | 2014-01539 | 2014-01540; **Taxon:** namePublishedIn: 1836; kingdom: Animalia; phylum: Arthropoda; class: Insecta; order: Hemiptera; family: Miridae; genus: Campyloneura; specificEpithet: virgula; scientificNameAuthorship: Herrich-Schaeffer; **Location:** country: Japan; stateProvince: Tokyo; municipality: Meguro-ku; locality: The University of Tokyo Campus, Komaba.; minimumElevationInMeters: 31; maximumElevationInMeters: 39; decimalLatitude: 35.66006; decimalLongitude: 139.68521; geodeticDatum: WGS84; **Identification:** identifiedBy: T. Ishikawa; dateIdentified: 2013; **Event:** samplingProtocol: net sweeping; eventDate: 2013-05-25/2013-05-28; **Record Level:** institutionCode: KMUT; collectionCode: IC
- **Type status:** Other material. **Occurrence:** recordedBy: T. Ishikawa; individualCount: 1; sex: 1 female; lifeStage: adult; otherCatalogNumbers: 2014-01541; **Taxon:** namePublishedIn: 1836; kingdom: Animalia; phylum: Arthropoda; class: Insecta; order: Hemiptera; family: Miridae; genus: Campyloneura; specificEpithet: virgula; scientificNameAuthorship: Herrich-Schaeffer; **Location:** country: Japan; stateProvince: Tokyo; municipality: Meguro-ku; locality: The University of Tokyo Campus, Komaba.; minimumElevationInMeters: 31; maximumElevationInMeters: 39; decimalLatitude: 35.66006; decimalLongitude: 139.68521; geodeticDatum: WGS84; **Identification:** identifiedBy: T. Ishikawa; dateIdentified: 2013; **Event:** samplingProtocol: net sweeping; eventDate: 2013-06-22; **Record Level:** institutionCode: KMUT; collectionCode: IC

##### Notes

Known as a recent alien species to Japan (Tokyo and Kanagawa Prefecture) (Yasunaga and Yamada 2014).

#### Castanopsides hasegawai

Yasunaga, 1992

##### Materials

- **Type status:** Other material. **Occurrence:** recordedBy: T. Ishikawa; individualCount: 1; sex: 1 female; lifeStage: adult; otherCatalogNumbers: 2014-00559; **Taxon:** namePublishedIn: 1992; kingdom: Animalia; phylum: Arthropoda; class: Insecta; order: Hemiptera; family: Miridae; genus: Castanopsides; specificEpithet: hasegawai; scientificNameAuthorship: Yasunaga; **Location:** country: Japan; stateProvince: Tokyo; municipality: Meguro-ku; locality: The University of Tokyo Campus, Komaba.; minimumElevationInMeters: 31; maximumElevationInMeters: 39; decimalLatitude: 35.66006; decimalLongitude: 139.68521; geodeticDatum: WGS84; **Identification:** identifiedBy: T. Ishikawa; dateIdentified: 2013; **Event:** samplingProtocol: light trap; eventDate: 2013-05-10; **Record Level:** institutionCode: KMUT; collectionCode: IC
- **Type status:** Other material. **Occurrence:** recordedBy: T. Ishikawa; individualCount: 2; sex: 2 females; lifeStage: adult; otherCatalogNumbers: 2014-00560 | 2014-00561; **Taxon:** namePublishedIn: 1992; kingdom: Animalia; phylum: Arthropoda; class: Insecta; order: Hemiptera; family: Miridae; genus: Castanopsides; specificEpithet: hasegawai; scientificNameAuthorship: Yasunaga; **Location:** country: Japan; stateProvince: Tokyo; municipality: Meguro-ku; locality: The University of Tokyo Campus, Komaba.; minimumElevationInMeters: 31; maximumElevationInMeters: 39; decimalLatitude: 35.66006; decimalLongitude: 139.68521; geodeticDatum: WGS84; **Identification:** identifiedBy: T. Ishikawa; dateIdentified: 2013; **Event:** samplingProtocol: net sweeping; eventDate: 2013-05-18; **Record Level:** institutionCode: KMUT; collectionCode: IC

#### Charagochilus angusticollis

Linnavuori, 1961

##### Materials

- **Type status:** Other material. **Occurrence:** recordedBy: T. Ishikawa; individualCount: 1; sex: 1 female; lifeStage: adult; otherCatalogNumbers: 2014-00562; **Taxon:** namePublishedIn: 1961; kingdom: Animalia; phylum: Arthropoda; class: Insecta; order: Hemiptera; family: Miridae; genus: Charagochilus; specificEpithet: angusticollis; scientificNameAuthorship: Linnavuori; **Location:** country: Japan; stateProvince: Tokyo; municipality: Meguro-ku; locality: The University of Tokyo Campus, Komaba.; minimumElevationInMeters: 31; maximumElevationInMeters: 39; decimalLatitude: 35.66006; decimalLongitude: 139.68521; geodeticDatum: WGS84; **Identification:** identifiedBy: T. Ishikawa; dateIdentified: 2013; **Event:** samplingProtocol: net sweeping; eventDate: 2013-04-28; **Record Level:** institutionCode: KMUT; collectionCode: IC
- **Type status:** Other material. **Occurrence:** recordedBy: T. Ishikawa; individualCount: 2; sex: 2 females; lifeStage: adult; otherCatalogNumbers: 2014-00563 | 2014-00564; **Taxon:** namePublishedIn: 1961; kingdom: Animalia; phylum: Arthropoda; class: Insecta; order: Hemiptera; family: Miridae; genus: Charagochilus; specificEpithet: angusticollis; scientificNameAuthorship: Linnavuori; **Location:** country: Japan; stateProvince: Tokyo; municipality: Meguro-ku; locality: The University of Tokyo Campus, Komaba.; minimumElevationInMeters: 31; maximumElevationInMeters: 39; decimalLatitude: 35.66006; decimalLongitude: 139.68521; geodeticDatum: WGS84; **Identification:** identifiedBy: T. Ishikawa; dateIdentified: 2013; **Event:** samplingProtocol: net sweeping; eventDate: 2013-05-25; **Record Level:** institutionCode: KMUT; collectionCode: IC
- **Type status:** Other material. **Occurrence:** recordedBy: T. Ishikawa; individualCount: 14; sex: 6 males, 8 females; lifeStage: adult; otherCatalogNumbers: 2014-00565 | 2014-00566 | 2014-00567 | 2014-00568 | 2014-00569 | 2014-00570 | 2014-00571 | 2014-00572 | 2014-00573 | 2014-00574 | 2014-00575 | 2014-00576 | 2014-00577 | 2014-00578; **Taxon:** namePublishedIn: 1961; kingdom: Animalia; phylum: Arthropoda; class: Insecta; order: Hemiptera; family: Miridae; genus: Charagochilus; specificEpithet: angusticollis; scientificNameAuthorship: Linnavuori; **Location:** country: Japan; stateProvince: Tokyo; municipality: Meguro-ku; locality: The University of Tokyo Campus, Komaba.; minimumElevationInMeters: 31; maximumElevationInMeters: 39; decimalLatitude: 35.66006; decimalLongitude: 139.68521; geodeticDatum: WGS84; **Identification:** identifiedBy: T. Ishikawa; dateIdentified: 2013; **Event:** samplingProtocol: net sweeping; eventDate: 2013-08-15; **Record Level:** institutionCode: KMUT; collectionCode: IC
- **Type status:** Other material. **Occurrence:** recordedBy: T. Ishikawa; individualCount: 7; sex: 3 males, 4 females; lifeStage: adult; otherCatalogNumbers: 2014-00579 | 2014-00580 | 2014-00581 | 2014-00582 | 2014-00583 | 2014-00584 | 2014-00585; **Taxon:** namePublishedIn: 1961; kingdom: Animalia; phylum: Arthropoda; class: Insecta; order: Hemiptera; family: Miridae; genus: Charagochilus; specificEpithet: angusticollis; scientificNameAuthorship: Linnavuori; **Location:** country: Japan; stateProvince: Tokyo; municipality: Meguro-ku; locality: The University of Tokyo Campus, Komaba.; minimumElevationInMeters: 31; maximumElevationInMeters: 39; decimalLatitude: 35.66006; decimalLongitude: 139.68521; geodeticDatum: WGS84; **Identification:** identifiedBy: T. Ishikawa; dateIdentified: 2013; **Event:** samplingProtocol: net sweeping; eventDate: 2013-10-30; **Record Level:** institutionCode: KMUT; collectionCode: IC

#### Cimidaeorus hasegawai

Nakatani, Yasunaga et Takai, 2000

##### Materials

- **Type status:** Other material. **Occurrence:** recordedBy: T. Ishikawa; individualCount: 1; sex: 1 female; lifeStage: adult; otherCatalogNumbers: 2014-00371; **Taxon:** namePublishedIn: 2000; kingdom: Animalia; phylum: Arthropoda; class: Insecta; order: Hemiptera; family: Miridae; genus: Cimidaeorus; specificEpithet: hasegawai; scientificNameAuthorship: Nakatani, Yasunaga et Takai; **Location:** country: Japan; stateProvince: Tokyo; municipality: Meguro-ku; locality: The University of Tokyo Campus, Komaba.; minimumElevationInMeters: 31; maximumElevationInMeters: 39; decimalLatitude: 35.66006; decimalLongitude: 139.68521; geodeticDatum: WGS84; **Identification:** identifiedBy: T. Ishikawa; dateIdentified: 2013; **Event:** samplingProtocol: net sweeping; eventDate: 2013-05-04; **Record Level:** institutionCode: KMUT; collectionCode: IC

#### Coridromius chinensis

Liu et Zhao, 1999

##### Materials

- **Type status:** Other material. **Occurrence:** recordedBy: T. Ishikawa; individualCount: 1; sex: 1 male; lifeStage: adult; otherCatalogNumbers: 2014-00151; **Taxon:** namePublishedIn: 1999; kingdom: Animalia; phylum: Arthropoda; class: Insecta; order: Hemiptera; family: Miridae; genus: Coridromius; specificEpithet: chinensis; scientificNameAuthorship: Liu et Zhao; **Location:** country: Japan; stateProvince: Tokyo; municipality: Meguro-ku; locality: The University of Tokyo Campus, Komaba.; minimumElevationInMeters: 31; maximumElevationInMeters: 39; decimalLatitude: 35.66006; decimalLongitude: 139.68521; geodeticDatum: WGS84; **Identification:** identifiedBy: T. Ishikawa; dateIdentified: 2013; **Event:** samplingProtocol: net sweeping; eventDate: 2013-06-16; **Record Level:** institutionCode: KMUT; collectionCode: IC

#### Creontiades coloripes

Hsiao, 1963

##### Materials

- **Type status:** Other material. **Occurrence:** recordedBy: T. Ishikawa; individualCount: 3; sex: 2 males, 1 female; lifeStage: adult; otherCatalogNumbers: 2014-00586 | 2014-00587 | 2014-00588; **Taxon:** namePublishedIn: 1963; kingdom: Animalia; phylum: Arthropoda; class: Insecta; order: Hemiptera; family: Miridae; genus: Creontiades; specificEpithet: coloripes; scientificNameAuthorship: Hsiao; **Location:** country: Japan; stateProvince: Tokyo; municipality: Meguro-ku; locality: The University of Tokyo Campus, Komaba.; minimumElevationInMeters: 31; maximumElevationInMeters: 39; decimalLatitude: 35.66006; decimalLongitude: 139.68521; geodeticDatum: WGS84; **Identification:** identifiedBy: T. Ishikawa; dateIdentified: 2013; **Event:** samplingProtocol: net sweeping; eventDate: 2013-08-15; **Record Level:** institutionCode: KMUT; collectionCode: IC

#### Dryophilocoris miyamotoi

Yasunaga, 1999

##### Materials

- **Type status:** Other material. **Occurrence:** recordedBy: T. Kato; individualCount: 1; sex: 1 female; lifeStage: adult; otherCatalogNumbers: 2014-00152; **Taxon:** namePublishedIn: 1999; kingdom: Animalia; phylum: Arthropoda; class: Insecta; order: Hemiptera; family: Miridae; genus: Dryophilocoris; specificEpithet: miyamotoi; scientificNameAuthorship: Yasunaga; **Location:** country: Japan; stateProvince: Tokyo; municipality: Meguro-ku; locality: The University of Tokyo Campus, Komaba.; minimumElevationInMeters: 31; maximumElevationInMeters: 39; decimalLatitude: 35.66006; decimalLongitude: 139.68521; geodeticDatum: WGS84; **Identification:** identifiedBy: T. Ishikawa; dateIdentified: 2013; **Event:** samplingProtocol: net sweeping; eventDate: 2013-04-27; **Record Level:** institutionCode: KMUT; collectionCode: IC
- **Type status:** Other material. **Occurrence:** recordedBy: T. Ishikawa; individualCount: 2; sex: 2 females; lifeStage: adult; otherCatalogNumbers: 2014-00153 | 2014-00154; **Taxon:** namePublishedIn: 1999; kingdom: Animalia; phylum: Arthropoda; class: Insecta; order: Hemiptera; family: Miridae; genus: Dryophilocoris; specificEpithet: miyamotoi; scientificNameAuthorship: Yasunaga; **Location:** country: Japan; stateProvince: Tokyo; municipality: Meguro-ku; locality: The University of Tokyo Campus, Komaba.; minimumElevationInMeters: 31; maximumElevationInMeters: 39; decimalLatitude: 35.66006; decimalLongitude: 139.68521; geodeticDatum: WGS84; **Identification:** identifiedBy: T. Ishikawa; dateIdentified: 2013; **Event:** samplingProtocol: net sweeping; eventDate: 2013-05-01; **Record Level:** institutionCode: KMUT; collectionCode: IC

#### Eurystylus coelestialium

(Kirkaldy, 1902)

##### Materials

- **Type status:** Other material. **Occurrence:** recordedBy: T. Ishikawa; individualCount: 6; sex: 1 male, 5 females; lifeStage: adult; otherCatalogNumbers: 2014-00589 | 2014-00590 | 2014-00591 | 2014-00592 | 2014-00593 | 2014-00594; **Taxon:** namePublishedIn: 1902; kingdom: Animalia; phylum: Arthropoda; class: Insecta; order: Hemiptera; family: Miridae; genus: Eurystylus; specificEpithet: coelestialium; scientificNameAuthorship: Kirkaldy; **Location:** country: Japan; stateProvince: Tokyo; municipality: Meguro-ku; locality: The University of Tokyo Campus, Komaba.; minimumElevationInMeters: 31; maximumElevationInMeters: 39; decimalLatitude: 35.66006; decimalLongitude: 139.68521; geodeticDatum: WGS84; **Identification:** identifiedBy: T. Ishikawa; dateIdentified: 2013; **Event:** samplingProtocol: net sweeping; eventDate: 2013-05-25; **Record Level:** institutionCode: KMUT; collectionCode: IC
- **Type status:** Other material. **Occurrence:** recordedBy: T. Ishikawa; individualCount: 6; sex: 3 males, 3 females; lifeStage: adult; otherCatalogNumbers: 2014-00595 | 2014-00596 | 2014-00597 | 2014-00598 | 2014-00599 | 2014-00600; **Taxon:** namePublishedIn: 1902; kingdom: Animalia; phylum: Arthropoda; class: Insecta; order: Hemiptera; family: Miridae; genus: Eurystylus; specificEpithet: coelestialium; scientificNameAuthorship: Kirkaldy; **Location:** country: Japan; stateProvince: Tokyo; municipality: Meguro-ku; locality: The University of Tokyo Campus, Komaba.; minimumElevationInMeters: 31; maximumElevationInMeters: 39; decimalLatitude: 35.66006; decimalLongitude: 139.68521; geodeticDatum: WGS84; **Identification:** identifiedBy: T. Ishikawa; dateIdentified: 2013; **Event:** samplingProtocol: net sweeping; eventDate: 2013-06-16/2013-06-18; **Record Level:** institutionCode: KMUT; collectionCode: IC
- **Type status:** Other material. **Occurrence:** recordedBy: T. Ishikawa; individualCount: 1; sex: 1 female; lifeStage: adult; otherCatalogNumbers: 2014-00601; **Taxon:** namePublishedIn: 1902; kingdom: Animalia; phylum: Arthropoda; class: Insecta; order: Hemiptera; family: Miridae; genus: Eurystylus; specificEpithet: coelestialium; scientificNameAuthorship: Kirkaldy; **Location:** country: Japan; stateProvince: Tokyo; municipality: Meguro-ku; locality: The University of Tokyo Campus, Komaba.; minimumElevationInMeters: 31; maximumElevationInMeters: 39; decimalLatitude: 35.66006; decimalLongitude: 139.68521; geodeticDatum: WGS84; **Identification:** identifiedBy: T. Ishikawa; dateIdentified: 2013; **Event:** samplingProtocol: net sweeping; eventDate: 2013-06-22; **Record Level:** institutionCode: KMUT; collectionCode: IC

#### Eurystylus luteus

Hsiao, 1941

##### Materials

- **Type status:** Other material. **Occurrence:** recordedBy: T. Ishikawa; individualCount: 4; sex: 3 males, 1 female; lifeStage: adult; otherCatalogNumbers: 2014-00602 | 2014-00603 | 2014-00604 | 2014-00605; **Taxon:** namePublishedIn: 1941; kingdom: Animalia; phylum: Arthropoda; class: Insecta; order: Hemiptera; family: Miridae; genus: Eurystylus; specificEpithet: luteus; scientificNameAuthorship: Hsiao; **Location:** country: Japan; stateProvince: Tokyo; municipality: Meguro-ku; locality: The University of Tokyo Campus, Komaba.; minimumElevationInMeters: 31; maximumElevationInMeters: 39; decimalLatitude: 35.66006; decimalLongitude: 139.68521; geodeticDatum: WGS84; **Identification:** identifiedBy: T. Ishikawa; dateIdentified: 2013; **Event:** samplingProtocol: net sweeping; eventDate: 2013-06-22/2013-06-23; **Record Level:** institutionCode: KMUT; collectionCode: IC
- **Type status:** Other material. **Occurrence:** recordedBy: T. Kato; individualCount: 1; sex: 1 female; lifeStage: adult; otherCatalogNumbers: 2014-00606; **Taxon:** namePublishedIn: 1941; kingdom: Animalia; phylum: Arthropoda; class: Insecta; order: Hemiptera; family: Miridae; genus: Eurystylus; specificEpithet: luteus; scientificNameAuthorship: Hsiao; **Location:** country: Japan; stateProvince: Tokyo; municipality: Meguro-ku; locality: The University of Tokyo Campus, Komaba.; minimumElevationInMeters: 31; maximumElevationInMeters: 39; decimalLatitude: 35.66006; decimalLongitude: 139.68521; geodeticDatum: WGS84; **Identification:** identifiedBy: T. Ishikawa; dateIdentified: 2013; **Event:** samplingProtocol: net sweeping; eventDate: 2013-10-14; **Record Level:** institutionCode: KMUT; collectionCode: IC

#### Harpocera orientalis

Kerzhner, 1979

##### Materials

- **Type status:** Other material. **Occurrence:** recordedBy: T. Ishikawa; individualCount: 1; sex: 1 female; lifeStage: adult; otherCatalogNumbers: 2014-00202; **Taxon:** namePublishedIn: 1979; kingdom: Animalia; phylum: Arthropoda; class: Insecta; order: Hemiptera; family: Miridae; genus: Harpocera; specificEpithet: orientalis; scientificNameAuthorship: Kerzhner; **Location:** country: Japan; stateProvince: Tokyo; municipality: Meguro-ku; locality: The University of Tokyo Campus, Komaba.; minimumElevationInMeters: 31; maximumElevationInMeters: 39; decimalLatitude: 35.66006; decimalLongitude: 139.68521; geodeticDatum: WGS84; **Identification:** identifiedBy: T. Ishikawa; dateIdentified: 2013; **Event:** samplingProtocol: net sweeping; eventDate: 2013-05-01; **Record Level:** institutionCode: KMUT; collectionCode: IC

#### Kasumiphylus kyushuensis

(Linnavuori, 1961)

##### Materials

- **Type status:** Other material. **Occurrence:** recordedBy: T. Ishikawa; individualCount: 7; sex: 2 males, 5 females; lifeStage: adult; otherCatalogNumbers: 2014-00203 | 2014-00204 | 2014-00205 | 2014-00206 | 2014-00207 | 2014-00320 | 2014-00322; **Taxon:** namePublishedIn: 1961; kingdom: Animalia; phylum: Arthropoda; class: Insecta; order: Hemiptera; family: Miridae; genus: Kasumiphylus; specificEpithet: kyushuensis; scientificNameAuthorship: Linnavuori; **Location:** country: Japan; stateProvince: Tokyo; municipality: Meguro-ku; locality: The University of Tokyo Campus, Komaba.; minimumElevationInMeters: 31; maximumElevationInMeters: 39; decimalLatitude: 35.66006; decimalLongitude: 139.68521; geodeticDatum: WGS84; **Identification:** identifiedBy: T. Ishikawa; dateIdentified: 2013; **Event:** samplingProtocol: light trap; eventDate: 2013-08-13; **Record Level:** institutionCode: KMUT; collectionCode: IC
- **Type status:** Other material. **Occurrence:** recordedBy: T. Ishikawa; individualCount: 1; sex: 1 male; lifeStage: adult; otherCatalogNumbers: 2014-00208; **Taxon:** namePublishedIn: 1961; kingdom: Animalia; phylum: Arthropoda; class: Insecta; order: Hemiptera; family: Miridae; genus: Kasumiphylus; specificEpithet: kyushuensis; scientificNameAuthorship: Linnavuori; **Location:** country: Japan; stateProvince: Tokyo; municipality: Meguro-ku; locality: The University of Tokyo Campus, Komaba.; minimumElevationInMeters: 31; maximumElevationInMeters: 39; decimalLatitude: 35.66006; decimalLongitude: 139.68521; geodeticDatum: WGS84; **Identification:** identifiedBy: T. Ishikawa; dateIdentified: 2013; **Event:** samplingProtocol: net sweeping; eventDate: 2013-08-18; **Record Level:** institutionCode: KMUT; collectionCode: IC

#### Monalocoris filicis

(Linnaeus, 1758)

##### Materials

- **Type status:** Other material. **Occurrence:** recordedBy: T. Ishikawa; individualCount: 1; sex: 1 male; lifeStage: adult; otherCatalogNumbers: 2014-00327; **Taxon:** namePublishedIn: 1758; kingdom: Animalia; phylum: Arthropoda; class: Insecta; order: Hemiptera; family: Miridae; genus: Monalocoris; specificEpithet: filicis; scientificNameAuthorship: Linnaeus; **Location:** country: Japan; stateProvince: Tokyo; municipality: Meguro-ku; locality: The University of Tokyo Campus, Komaba.; minimumElevationInMeters: 31; maximumElevationInMeters: 39; decimalLatitude: 35.66006; decimalLongitude: 139.68521; geodeticDatum: WGS84; **Identification:** identifiedBy: T. Ishikawa; dateIdentified: 2013; **Event:** samplingProtocol: net sweeping; eventDate: 2013-05-18; **Record Level:** institutionCode: KMUT; collectionCode: IC
- **Type status:** Other material. **Occurrence:** recordedBy: T. Ishikawa; individualCount: 29; sex: 16 males, 13 females; lifeStage: adult; otherCatalogNumbers: 2014-00328 | 2014-00329 | 2014-00330 | 2014-00331 | 2014-00332 | 2014-00333 | 2014-00334 | 2014-00335 | 2014-00336 | 2014-00337 | 2014-00338 | 2014-00339 | 2014-00340 | 2014-00341 | 2014-00342 | 2014-00343 | 2014-00344 | 2014-00345 | 2014-00346 | 2014-00347 | 2014-00348 | 2014-00349 | 2014-00350 | 2014-00351 | 2014-00352 | 2014-00353 | 2014-00354 | 2014-00355 | 2014-00356; **Taxon:** namePublishedIn: 1758; kingdom: Animalia; phylum: Arthropoda; class: Insecta; order: Hemiptera; family: Miridae; genus: Monalocoris; specificEpithet: filicis; scientificNameAuthorship: Linnaeus; **Location:** country: Japan; stateProvince: Tokyo; municipality: Meguro-ku; locality: The University of Tokyo Campus, Komaba.; minimumElevationInMeters: 31; maximumElevationInMeters: 39; decimalLatitude: 35.66006; decimalLongitude: 139.68521; geodeticDatum: WGS84; **Identification:** identifiedBy: T. Ishikawa; dateIdentified: 2013; **Event:** samplingProtocol: net sweeping; eventDate: 2013-08-15/2013-08-18; **Record Level:** institutionCode: KMUT; collectionCode: IC
- **Type status:** Other material. **Occurrence:** recordedBy: T. Ishikawa; individualCount: 1; sex: 1 male; lifeStage: adult; otherCatalogNumbers: 2014-00357; **Taxon:** namePublishedIn: 1758; kingdom: Animalia; phylum: Arthropoda; class: Insecta; order: Hemiptera; family: Miridae; genus: Monalocoris; specificEpithet: filicis; scientificNameAuthorship: Linnaeus; **Location:** country: Japan; stateProvince: Tokyo; municipality: Meguro-ku; locality: The University of Tokyo Campus, Komaba.; minimumElevationInMeters: 31; maximumElevationInMeters: 39; decimalLatitude: 35.66006; decimalLongitude: 139.68521; geodeticDatum: WGS84; **Identification:** identifiedBy: T. Ishikawa; dateIdentified: 2013; **Event:** samplingProtocol: net sweeping; eventDate: 2013-09-06; **Record Level:** institutionCode: KMUT; collectionCode: IC
- **Type status:** Other material. **Occurrence:** recordedBy: T. Ishikawa; individualCount: 5; sex: 4 males, 1 female; lifeStage: adult; otherCatalogNumbers: 2014-00358 | 2014-00359 | 2014-00360 | 2014-00361 | 2014-00362; **Taxon:** namePublishedIn: 1758; kingdom: Animalia; phylum: Arthropoda; class: Insecta; order: Hemiptera; family: Miridae; genus: Monalocoris; specificEpithet: filicis; scientificNameAuthorship: Linnaeus; **Location:** country: Japan; stateProvince: Tokyo; municipality: Meguro-ku; locality: The University of Tokyo Campus, Komaba.; minimumElevationInMeters: 31; maximumElevationInMeters: 39; decimalLatitude: 35.66006; decimalLongitude: 139.68521; geodeticDatum: WGS84; **Identification:** identifiedBy: T. Ishikawa; dateIdentified: 2013; **Event:** samplingProtocol: net sweeping; eventDate: 2013-10-21; **Record Level:** institutionCode: KMUT; collectionCode: IC
- **Type status:** Other material. **Occurrence:** recordedBy: T. Ishikawa; individualCount: 8; sex: 2 males, 6 females; lifeStage: adult; otherCatalogNumbers: 2014-00363 | 2014-00364 | 2014-00365 | 2014-00366 | 2014-00367 | 2014-00368 | 2014-00369 | 2014-00370; **Taxon:** namePublishedIn: 1758; kingdom: Animalia; phylum: Arthropoda; class: Insecta; order: Hemiptera; family: Miridae; genus: Monalocoris; specificEpithet: filicis; scientificNameAuthorship: Linnaeus; **Location:** country: Japan; stateProvince: Tokyo; municipality: Meguro-ku; locality: The University of Tokyo Campus, Komaba.; minimumElevationInMeters: 31; maximumElevationInMeters: 39; decimalLatitude: 35.66006; decimalLongitude: 139.68521; geodeticDatum: WGS84; **Identification:** identifiedBy: T. Ishikawa; dateIdentified: 2013; **Event:** samplingProtocol: net sweeping; eventDate: 2013-10-30; **Record Level:** institutionCode: KMUT; collectionCode: IC

#### Neolygus pteleinus

(Kerzhner, 1977)

##### Materials

- **Type status:** Other material. **Occurrence:** recordedBy: T. Ishikawa; individualCount: 1; sex: 1 female; lifeStage: adult; otherCatalogNumbers: 2014-00607; **Taxon:** namePublishedIn: 1977; kingdom: Animalia; phylum: Arthropoda; class: Insecta; order: Hemiptera; family: Miridae; genus: Neolygus; specificEpithet: pteleinus; scientificNameAuthorship: Kerzhner; **Location:** country: Japan; stateProvince: Tokyo; municipality: Meguro-ku; locality: The University of Tokyo Campus, Komaba.; minimumElevationInMeters: 31; maximumElevationInMeters: 39; decimalLatitude: 35.66006; decimalLongitude: 139.68521; geodeticDatum: WGS84; **Identification:** identifiedBy: T. Ishikawa; dateIdentified: 2013; **Event:** samplingProtocol: net sweeping; eventDate: 2013-04-28; **Record Level:** institutionCode: KMUT; collectionCode: IC
- **Type status:** Other material. **Occurrence:** recordedBy: T. Ishikawa; individualCount: 7; sex: 3 males, 4 females; lifeStage: adult; otherCatalogNumbers: 2014-00608 | 2014-00609 | 2014-00610 | 2014-00611 | 2014-00612 | 2014-00613 | 2014-00614; **Taxon:** namePublishedIn: 1977; kingdom: Animalia; phylum: Arthropoda; class: Insecta; order: Hemiptera; family: Miridae; genus: Neolygus; specificEpithet: pteleinus; scientificNameAuthorship: Kerzhner; **Location:** country: Japan; stateProvince: Tokyo; municipality: Meguro-ku; locality: The University of Tokyo Campus, Komaba.; minimumElevationInMeters: 31; maximumElevationInMeters: 39; decimalLatitude: 35.66006; decimalLongitude: 139.68521; geodeticDatum: WGS84; **Identification:** identifiedBy: T. Ishikawa; dateIdentified: 2013; **Event:** samplingProtocol: net sweeping; eventDate: 2013-05-01/2013-05-04; **Record Level:** institutionCode: KMUT; collectionCode: IC
- **Type status:** Other material. **Occurrence:** recordedBy: T. Ishikawa; individualCount: 1; sex: 1 female; lifeStage: adult; otherCatalogNumbers: 2014-00615; **Taxon:** namePublishedIn: 1977; kingdom: Animalia; phylum: Arthropoda; class: Insecta; order: Hemiptera; family: Miridae; genus: Neolygus; specificEpithet: pteleinus; scientificNameAuthorship: Kerzhner; **Location:** country: Japan; stateProvince: Tokyo; municipality: Meguro-ku; locality: The University of Tokyo Campus, Komaba.; minimumElevationInMeters: 31; maximumElevationInMeters: 39; decimalLatitude: 35.66006; decimalLongitude: 139.68521; geodeticDatum: WGS84; **Identification:** identifiedBy: T. Ishikawa; dateIdentified: 2013; **Event:** samplingProtocol: light trap; eventDate: 2013-05-12; **Record Level:** institutionCode: KMUT; collectionCode: IC
- **Type status:** Other material. **Occurrence:** recordedBy: T. Ishikawa; individualCount: 2; sex: 2 females; lifeStage: adult; otherCatalogNumbers: 2014-00616 | 2014-00617; **Taxon:** namePublishedIn: 1977; kingdom: Animalia; phylum: Arthropoda; class: Insecta; order: Hemiptera; family: Miridae; genus: Neolygus; specificEpithet: pteleinus; scientificNameAuthorship: Kerzhner; **Location:** country: Japan; stateProvince: Tokyo; municipality: Meguro-ku; locality: The University of Tokyo Campus, Komaba.; minimumElevationInMeters: 31; maximumElevationInMeters: 39; decimalLatitude: 35.66006; decimalLongitude: 139.68521; geodeticDatum: WGS84; **Identification:** identifiedBy: T. Ishikawa; dateIdentified: 2013; **Event:** samplingProtocol: net sweeping; eventDate: 2013-06-16/2013-06-18; **Record Level:** institutionCode: KMUT; collectionCode: IC

##### Notes

First record in Tokyo.

#### Philostephanus rubripes

(Jakovlev, 1876)

##### Materials

- **Type status:** Other material. **Occurrence:** recordedBy: T. Ishikawa; individualCount: 1; sex: 1 male; lifeStage: adult; otherCatalogNumbers: 2014-00618; **Taxon:** namePublishedIn: 1876; kingdom: Animalia; phylum: Arthropoda; class: Insecta; order: Hemiptera; family: Miridae; genus: Philostephanus; specificEpithet: rubripes; scientificNameAuthorship: Jakovlev; **Location:** country: Japan; stateProvince: Tokyo; municipality: Meguro-ku; locality: The University of Tokyo Campus, Komaba.; minimumElevationInMeters: 31; maximumElevationInMeters: 39; decimalLatitude: 35.66006; decimalLongitude: 139.68521; geodeticDatum: WGS84; **Identification:** identifiedBy: T. Ishikawa; dateIdentified: 2013; **Event:** samplingProtocol: net sweeping; eventDate: 2013-05-25; **Record Level:** institutionCode: KMUT; collectionCode: IC

#### Phylus miyamotoi

Yasunaga, 1999

##### Materials

- **Type status:** Other material. **Occurrence:** recordedBy: T. Ishikawa; individualCount: 1; sex: 1 female; lifeStage: adult; otherCatalogNumbers: 2014-00209; **Taxon:** namePublishedIn: 1999; kingdom: Animalia; phylum: Arthropoda; class: Insecta; order: Hemiptera; family: Miridae; genus: Phylus; specificEpithet: miyamotoi; scientificNameAuthorship: Yasunaga; **Location:** country: Japan; stateProvince: Tokyo; municipality: Meguro-ku; locality: The University of Tokyo Campus, Komaba.; minimumElevationInMeters: 31; maximumElevationInMeters: 39; decimalLatitude: 35.66006; decimalLongitude: 139.68521; geodeticDatum: WGS84; **Identification:** identifiedBy: T. Ishikawa; dateIdentified: 2013; **Event:** samplingProtocol: net sweeping; eventDate: 2013-04-29; **Record Level:** institutionCode: KMUT; collectionCode: IC
- **Type status:** Other material. **Occurrence:** recordedBy: T. Ishikawa; individualCount: 8; sex: 4 males, 4 females; lifeStage: adult; otherCatalogNumbers: 2014-00210 | 2014-00211 | 2014-00212 | 2014-00213 | 2014-00214 | 2014-00215 | 2014-00216 | 2014-00217; **Taxon:** namePublishedIn: 1999; kingdom: Animalia; phylum: Arthropoda; class: Insecta; order: Hemiptera; family: Miridae; genus: Phylus; specificEpithet: miyamotoi; scientificNameAuthorship: Yasunaga; **Location:** country: Japan; stateProvince: Tokyo; municipality: Meguro-ku; locality: The University of Tokyo Campus, Komaba.; minimumElevationInMeters: 31; maximumElevationInMeters: 39; decimalLatitude: 35.66006; decimalLongitude: 139.68521; geodeticDatum: WGS84; **Identification:** identifiedBy: T. Ishikawa; dateIdentified: 2013; **Event:** samplingProtocol: net sweeping; eventDate: 2013-05-04; **Record Level:** institutionCode: KMUT; collectionCode: IC
- **Type status:** Other material. **Occurrence:** recordedBy: T. Ishikawa; individualCount: 2; sex: 1 male, 1 female; lifeStage: adult; otherCatalogNumbers: 2014-00218 | 2014-00219; **Taxon:** namePublishedIn: 1999; kingdom: Animalia; phylum: Arthropoda; class: Insecta; order: Hemiptera; family: Miridae; genus: Phylus; specificEpithet: miyamotoi; scientificNameAuthorship: Yasunaga; **Location:** country: Japan; stateProvince: Tokyo; municipality: Meguro-ku; locality: The University of Tokyo Campus, Komaba.; minimumElevationInMeters: 31; maximumElevationInMeters: 39; decimalLatitude: 35.66006; decimalLongitude: 139.68521; geodeticDatum: WGS84; **Identification:** identifiedBy: T. Ishikawa; dateIdentified: 2013; **Event:** samplingProtocol: net sweeping; eventDate: 2013-05-12; **Record Level:** institutionCode: KMUT; collectionCode: IC
- **Type status:** Other material. **Occurrence:** recordedBy: T. Ishikawa; individualCount: 1; sex: 1 female; lifeStage: adult; otherCatalogNumbers: 2014-00220; **Taxon:** namePublishedIn: 1999; kingdom: Animalia; phylum: Arthropoda; class: Insecta; order: Hemiptera; family: Miridae; genus: Phylus; specificEpithet: miyamotoi; scientificNameAuthorship: Yasunaga; **Location:** country: Japan; stateProvince: Tokyo; municipality: Meguro-ku; locality: The University of Tokyo Campus, Komaba.; minimumElevationInMeters: 31; maximumElevationInMeters: 39; decimalLatitude: 35.66006; decimalLongitude: 139.68521; geodeticDatum: WGS84; **Identification:** identifiedBy: T. Ishikawa; dateIdentified: 2013; **Event:** samplingProtocol: net sweeping; eventDate: 2013-05-18; **Record Level:** institutionCode: KMUT; collectionCode: IC

##### Notes

First record in Tokyo.

#### Pilophorus setulosus

Horváth, 1905

##### Materials

- **Type status:** Other material. **Occurrence:** recordedBy: T. Ishikawa; individualCount: 7; sex: 4 males, 3 females; lifeStage: adult; otherCatalogNumbers: 2014-00172 | 2014-00173 | 2014-00174 | 2014-00175 | 2014-00176 | 2014-00177 | 2014-00178; **Taxon:** namePublishedIn: 1905; kingdom: Animalia; phylum: Arthropoda; class: Insecta; order: Hemiptera; family: Miridae; genus: Pilophorus; specificEpithet: setulosus; scientificNameAuthorship: Horváth; **Location:** country: Japan; stateProvince: Tokyo; municipality: Meguro-ku; locality: The University of Tokyo Campus, Komaba.; minimumElevationInMeters: 31; maximumElevationInMeters: 39; decimalLatitude: 35.66006; decimalLongitude: 139.68521; geodeticDatum: WGS84; **Identification:** identifiedBy: T. Ishikawa; dateIdentified: 2013; **Event:** samplingProtocol: net sweeping; eventDate: 2013-06-15/2013-06-18; **Record Level:** institutionCode: KMUT; collectionCode: IC
- **Type status:** Other material. **Occurrence:** recordedBy: T. Ishikawa; individualCount: 7; sex: 6 males, 1 female; lifeStage: adult; otherCatalogNumbers: 2014-00179 | 2014-00180 | 2014-00181 | 2014-00182 | 2014-00183 | 2014-00184 | 2014-00185; **Taxon:** namePublishedIn: 1905; kingdom: Animalia; phylum: Arthropoda; class: Insecta; order: Hemiptera; family: Miridae; genus: Pilophorus; specificEpithet: setulosus; scientificNameAuthorship: Horváth; **Location:** country: Japan; stateProvince: Tokyo; municipality: Meguro-ku; locality: The University of Tokyo Campus, Komaba.; minimumElevationInMeters: 31; maximumElevationInMeters: 39; decimalLatitude: 35.66006; decimalLongitude: 139.68521; geodeticDatum: WGS84; **Identification:** identifiedBy: T. Ishikawa; dateIdentified: 2013; **Event:** samplingProtocol: net sweeping; eventDate: 2013-06-22/2013-06-23; **Record Level:** institutionCode: KMUT; collectionCode: IC
- **Type status:** Other material. **Occurrence:** recordedBy: T. Ishikawa; individualCount: 2; sex: 1 male, 1 female; lifeStage: adult; otherCatalogNumbers: 2014-00186 | 2014-00187; **Taxon:** namePublishedIn: 1905; kingdom: Animalia; phylum: Arthropoda; class: Insecta; order: Hemiptera; family: Miridae; genus: Pilophorus; specificEpithet: setulosus; scientificNameAuthorship: Horváth; **Location:** country: Japan; stateProvince: Tokyo; municipality: Meguro-ku; locality: The University of Tokyo Campus, Komaba.; minimumElevationInMeters: 31; maximumElevationInMeters: 39; decimalLatitude: 35.66006; decimalLongitude: 139.68521; geodeticDatum: WGS84; **Identification:** identifiedBy: T. Ishikawa; dateIdentified: 2013; **Event:** samplingProtocol: net sweeping; eventDate: 2013-08-15; **Record Level:** institutionCode: KMUT; collectionCode: IC

#### Pilophorus typicus

(Dsitant, 1909)

##### Materials

- **Type status:** Other material. **Occurrence:** recordedBy: T. Ishikawa; individualCount: 2; sex: 2 females; lifeStage: adult; otherCatalogNumbers: 2014-00188 | 2014-00189; **Taxon:** namePublishedIn: 1909; kingdom: Animalia; phylum: Arthropoda; class: Insecta; order: Hemiptera; family: Miridae; genus: Pilophorus; specificEpithet: typicus; scientificNameAuthorship: Dsitant; **Location:** country: Japan; stateProvince: Tokyo; municipality: Meguro-ku; locality: The University of Tokyo Campus, Komaba.; minimumElevationInMeters: 31; maximumElevationInMeters: 39; decimalLatitude: 35.66006; decimalLongitude: 139.68521; geodeticDatum: WGS84; **Identification:** identifiedBy: T. Ishikawa; dateIdentified: 2013; **Event:** samplingProtocol: net sweeping; eventDate: 2013-06-22; **Record Level:** institutionCode: KMUT; collectionCode: IC
- **Type status:** Other material. **Occurrence:** recordedBy: T. Ishikawa; individualCount: 1; sex: 1 male; lifeStage: adult; otherCatalogNumbers: 2014-00190; **Taxon:** namePublishedIn: 1909; kingdom: Animalia; phylum: Arthropoda; class: Insecta; order: Hemiptera; family: Miridae; genus: Pilophorus; specificEpithet: typicus; scientificNameAuthorship: Dsitant; **Location:** country: Japan; stateProvince: Tokyo; municipality: Meguro-ku; locality: The University of Tokyo Campus, Komaba.; minimumElevationInMeters: 31; maximumElevationInMeters: 39; decimalLatitude: 35.66006; decimalLongitude: 139.68521; geodeticDatum: WGS84; **Identification:** identifiedBy: T. Ishikawa; dateIdentified: 2013; **Event:** samplingProtocol: light trap; eventDate: 2013-08-02; **Record Level:** institutionCode: KMUT; collectionCode: IC
- **Type status:** Other material. **Occurrence:** recordedBy: T. Ishikawa; individualCount: 3; sex: 3 females; lifeStage: adult; otherCatalogNumbers: 2014-00191 | 2014-00192 | 2014-00193; **Taxon:** namePublishedIn: 1909; kingdom: Animalia; phylum: Arthropoda; class: Insecta; order: Hemiptera; family: Miridae; genus: Pilophorus; specificEpithet: typicus; scientificNameAuthorship: Dsitant; **Location:** country: Japan; stateProvince: Tokyo; municipality: Meguro-ku; locality: The University of Tokyo Campus, Komaba.; minimumElevationInMeters: 31; maximumElevationInMeters: 39; decimalLatitude: 35.66006; decimalLongitude: 139.68521; geodeticDatum: WGS84; **Identification:** identifiedBy: T. Ishikawa; dateIdentified: 2013; **Event:** samplingProtocol: net sweeping; eventDate: 2013-08-18; **Record Level:** institutionCode: KMUT; collectionCode: IC
- **Type status:** Other material. **Occurrence:** recordedBy: T. Ishikawa; individualCount: 1; sex: 1 female; lifeStage: adult; otherCatalogNumbers: 2014-00194; **Taxon:** namePublishedIn: 1909; kingdom: Animalia; phylum: Arthropoda; class: Insecta; order: Hemiptera; family: Miridae; genus: Pilophorus; specificEpithet: typicus; scientificNameAuthorship: Dsitant; **Location:** country: Japan; stateProvince: Tokyo; municipality: Meguro-ku; locality: The University of Tokyo Campus, Komaba.; minimumElevationInMeters: 31; maximumElevationInMeters: 39; decimalLatitude: 35.66006; decimalLongitude: 139.68521; geodeticDatum: WGS84; **Identification:** identifiedBy: T. Ishikawa; dateIdentified: 2013; **Event:** samplingProtocol: net sweeping; eventDate: 2013-10-21; **Record Level:** institutionCode: KMUT; collectionCode: IC

#### Psallus bagjonicus

Josifov, 1983

##### Materials

- **Type status:** Other material. **Occurrence:** recordedBy: T. Ishikawa; individualCount: 9; sex: 4 males, 5 females; lifeStage: adult; otherCatalogNumbers: 2014-00265 | 2014-00266 | 2014-00267 | 2014-00268 | 2014-00269 | 2014-00270 | 2014-00271 | 2014-00272 | 2014-00273; **Taxon:** namePublishedIn: 1983; kingdom: Animalia; phylum: Arthropoda; class: Insecta; order: Hemiptera; family: Miridae; genus: Psallus; specificEpithet: bagjonicus; scientificNameAuthorship: Josifov; **Location:** country: Japan; stateProvince: Tokyo; municipality: Meguro-ku; locality: The University of Tokyo Campus, Komaba.; minimumElevationInMeters: 31; maximumElevationInMeters: 39; decimalLatitude: 35.66006; decimalLongitude: 139.68521; geodeticDatum: WGS84; **Identification:** identifiedBy: T. Ishikawa; dateIdentified: 2013; **Event:** samplingProtocol: net sweeping; eventDate: 2013-05-01/2013-05-04; **Record Level:** institutionCode: KMUT; collectionCode: IC

#### Psallus edoensis

Yasunaga et Vinokurov, 2000

##### Materials

- **Type status:** Other material. **Occurrence:** recordedBy: T. Kato; individualCount: 22; sex: 13 males, 9 females; lifeStage: adult; otherCatalogNumbers: 2014-00229 | 2014-00230 | 2014-00231 | 2014-00232 | 2014-00233 | 2014-00234 | 2014-00235 | 2014-00236 | 2014-00237 | 2014-00238 | 2014-00239 | 2014-00240 | 2014-00241 | 2014-00242 | 2014-00243 | 2014-00244 | 2014-00245 | 2014-00246 | 2014-00247 | 2014-00248 | 2014-00249 | 2014-00250; **Taxon:** namePublishedIn: 2000; kingdom: Animalia; phylum: Arthropoda; class: Insecta; order: Hemiptera; family: Miridae; genus: Psallus; specificEpithet: edoensis; scientificNameAuthorship: Yasunaga et Vinokurov; **Location:** country: Japan; stateProvince: Tokyo; municipality: Meguro-ku; locality: The University of Tokyo Campus, Komaba.; minimumElevationInMeters: 31; maximumElevationInMeters: 39; decimalLatitude: 35.66006; decimalLongitude: 139.68521; geodeticDatum: WGS84; **Identification:** identifiedBy: T. Ishikawa; dateIdentified: 2013; **Event:** samplingProtocol: net sweeping; eventDate: 2013-04-27/2013-04-29; **Record Level:** institutionCode: KMUT; collectionCode: IC
- **Type status:** Other material. **Occurrence:** recordedBy: T. Ishikawa; individualCount: 8; sex: 4 males, 4 females; lifeStage: adult; otherCatalogNumbers: 2014-00251 | 2014-00252 | 2014-00253 | 2014-00254 | 2014-00255 | 2014-00256 | 2014-00257 | 2014-00258; **Taxon:** namePublishedIn: 2000; kingdom: Animalia; phylum: Arthropoda; class: Insecta; order: Hemiptera; family: Miridae; genus: Psallus; specificEpithet: edoensis; scientificNameAuthorship: Yasunaga et Vinokurov; **Location:** country: Japan; stateProvince: Tokyo; municipality: Meguro-ku; locality: The University of Tokyo Campus, Komaba.; minimumElevationInMeters: 31; maximumElevationInMeters: 39; decimalLatitude: 35.66006; decimalLongitude: 139.68521; geodeticDatum: WGS84; **Identification:** identifiedBy: T. Ishikawa; dateIdentified: 2013; **Event:** samplingProtocol: net sweeping; eventDate: 2013-05-04; **Record Level:** institutionCode: KMUT; collectionCode: IC
- **Type status:** Other material. **Occurrence:** recordedBy: T. Ishikawa; individualCount: 2; sex: 1 male, 1 female; lifeStage: adult; otherCatalogNumbers: 2014-00259 | 2014-00260; **Taxon:** namePublishedIn: 2000; kingdom: Animalia; phylum: Arthropoda; class: Insecta; order: Hemiptera; family: Miridae; genus: Psallus; specificEpithet: edoensis; scientificNameAuthorship: Yasunaga et Vinokurov; **Location:** country: Japan; stateProvince: Tokyo; municipality: Meguro-ku; locality: The University of Tokyo Campus, Komaba.; minimumElevationInMeters: 31; maximumElevationInMeters: 39; decimalLatitude: 35.66006; decimalLongitude: 139.68521; geodeticDatum: WGS84; **Identification:** identifiedBy: T. Ishikawa; dateIdentified: 2013; **Event:** samplingProtocol: net sweeping; eventDate: 2013-05-12; **Record Level:** institutionCode: KMUT; collectionCode: IC
- **Type status:** Other material. **Occurrence:** recordedBy: T. Ishikawa; individualCount: 1; sex: 1 male; lifeStage: adult; otherCatalogNumbers: 2014-00261; **Taxon:** namePublishedIn: 2000; kingdom: Animalia; phylum: Arthropoda; class: Insecta; order: Hemiptera; family: Miridae; genus: Psallus; specificEpithet: edoensis; scientificNameAuthorship: Yasunaga et Vinokurov; **Location:** country: Japan; stateProvince: Tokyo; municipality: Meguro-ku; locality: The University of Tokyo Campus, Komaba.; minimumElevationInMeters: 31; maximumElevationInMeters: 39; decimalLatitude: 35.66006; decimalLongitude: 139.68521; geodeticDatum: WGS84; **Identification:** identifiedBy: T. Ishikawa; dateIdentified: 2013; **Event:** samplingProtocol: net sweeping; eventDate: 2013-05-18; **Record Level:** institutionCode: KMUT; collectionCode: IC
- **Type status:** Other material. **Occurrence:** recordedBy: T. Ishikawa; individualCount: 3; sex: 1 male, 2 females; lifeStage: adult; otherCatalogNumbers: 2014-00262 | 2014-00263 | 2014-00264; **Taxon:** namePublishedIn: 2000; kingdom: Animalia; phylum: Arthropoda; class: Insecta; order: Hemiptera; family: Miridae; genus: Psallus; specificEpithet: edoensis; scientificNameAuthorship: Yasunaga et Vinokurov; **Location:** country: Japan; stateProvince: Tokyo; municipality: Meguro-ku; locality: The University of Tokyo Campus, Komaba.; minimumElevationInMeters: 31; maximumElevationInMeters: 39; decimalLatitude: 35.66006; decimalLongitude: 139.68521; geodeticDatum: WGS84; **Identification:** identifiedBy: T. Ishikawa; dateIdentified: 2013; **Event:** samplingProtocol: net sweeping; eventDate: 2013-05-25; **Record Level:** institutionCode: KMUT; collectionCode: IC

##### Notes

Recently rediscovered after being undetected for 59 years (Ishikawa et al. 2014).

#### Psallus roseoguttatus

Yasunaga et Vinokurov, 2000

##### Materials

- **Type status:** Other material. **Occurrence:** recordedBy: T. Ishikawa; individualCount: 5; sex: 4 males, 1 female; lifeStage: adult; otherCatalogNumbers: 2014-00221 | 2014-00222 | 2014-00223 | 2014-00224 | 2014-00225; **Taxon:** namePublishedIn: 2000; kingdom: Animalia; phylum: Arthropoda; class: Insecta; order: Hemiptera; family: Miridae; genus: Psallus; specificEpithet: roseoguttatus; scientificNameAuthorship: Yasunaga et Vinokurov; **Location:** country: Japan; stateProvince: Tokyo; municipality: Meguro-ku; locality: The University of Tokyo Campus, Komaba.; minimumElevationInMeters: 31; maximumElevationInMeters: 39; decimalLatitude: 35.66006; decimalLongitude: 139.68521; geodeticDatum: WGS84; **Identification:** identifiedBy: T. Ishikawa; dateIdentified: 2013; **Event:** samplingProtocol: net sweeping; eventDate: 2013-05-04; **Record Level:** institutionCode: KMUT; collectionCode: IC
- **Type status:** Other material. **Occurrence:** recordedBy: T. Ishikawa; individualCount: 1; sex: 1 male; lifeStage: adult; otherCatalogNumbers: 2014-00226; **Taxon:** namePublishedIn: 2000; kingdom: Animalia; phylum: Arthropoda; class: Insecta; order: Hemiptera; family: Miridae; genus: Psallus; specificEpithet: roseoguttatus; scientificNameAuthorship: Yasunaga et Vinokurov; **Location:** country: Japan; stateProvince: Tokyo; municipality: Meguro-ku; locality: The University of Tokyo Campus, Komaba.; minimumElevationInMeters: 31; maximumElevationInMeters: 39; decimalLatitude: 35.66006; decimalLongitude: 139.68521; geodeticDatum: WGS84; **Identification:** identifiedBy: T. Ishikawa; dateIdentified: 2013; **Event:** samplingProtocol: net sweeping; eventDate: 2013-05-12; **Record Level:** institutionCode: KMUT; collectionCode: IC
- **Type status:** Other material. **Occurrence:** recordedBy: T. Ishikawa; individualCount: 2; sex: 2 males; lifeStage: adult; otherCatalogNumbers: 2014-00227 | 2014-00228; **Taxon:** namePublishedIn: 2000; kingdom: Animalia; phylum: Arthropoda; class: Insecta; order: Hemiptera; family: Miridae; genus: Psallus; specificEpithet: roseoguttatus; scientificNameAuthorship: Yasunaga et Vinokurov; **Location:** country: Japan; stateProvince: Tokyo; municipality: Meguro-ku; locality: The University of Tokyo Campus, Komaba.; minimumElevationInMeters: 31; maximumElevationInMeters: 39; decimalLatitude: 35.66006; decimalLongitude: 139.68521; geodeticDatum: WGS84; **Identification:** identifiedBy: T. Ishikawa; dateIdentified: 2013; **Event:** samplingProtocol: net sweeping; eventDate: 2013-05-18; **Record Level:** institutionCode: KMUT; collectionCode: IC

##### Notes

First record from eastern Japan as well as Tokyo.

#### Pseudoloxops miyamotoi

Yasunaga, 1997

##### Materials

- **Type status:** Other material. **Occurrence:** recordedBy: T. Ishikawa; individualCount: 1; sex: 1 male; lifeStage: adult; otherCatalogNumbers: 2014-00155; **Taxon:** namePublishedIn: 1997; kingdom: Animalia; phylum: Arthropoda; class: Insecta; order: Hemiptera; family: Miridae; genus: Pseudoloxops; specificEpithet: miyamotoi; scientificNameAuthorship: Yasunaga; **Location:** country: Japan; stateProvince: Tokyo; municipality: Meguro-ku; locality: The University of Tokyo Campus, Komaba.; minimumElevationInMeters: 31; maximumElevationInMeters: 39; decimalLatitude: 35.66006; decimalLongitude: 139.68521; geodeticDatum: WGS84; **Identification:** identifiedBy: T. Ishikawa; dateIdentified: 2013; **Event:** samplingProtocol: net sweeping; eventDate: 2013-06-22; **Record Level:** institutionCode: KMUT; collectionCode: IC

##### Notes

First record from eastern Japan as well as Tokyo.

#### Pseudophylus flavipes

(Nitobe, 1906)

##### Materials

- **Type status:** Other material. **Occurrence:** recordedBy: T. Ishikawa; individualCount: 5; sex: 2 males, 3 females; lifeStage: adult; otherCatalogNumbers: 2014-00274 | 2014-00275 | 2014-00276 | 2014-00277 | 2014-00278; **Taxon:** namePublishedIn: 1906; kingdom: Animalia; phylum: Arthropoda; class: Insecta; order: Hemiptera; family: Miridae; genus: Pseudophylus; specificEpithet: flavipes; scientificNameAuthorship: Nitobe; **Location:** country: Japan; stateProvince: Tokyo; municipality: Meguro-ku; locality: The University of Tokyo Campus, Komaba.; minimumElevationInMeters: 31; maximumElevationInMeters: 39; decimalLatitude: 35.66006; decimalLongitude: 139.68521; geodeticDatum: WGS84; **Identification:** identifiedBy: T. Ishikawa; dateIdentified: 2013; **Event:** samplingProtocol: net sweeping; eventDate: 2013-04-28/2013-04-29; **Record Level:** institutionCode: KMUT; collectionCode: IC
- **Type status:** Other material. **Occurrence:** recordedBy: T. Ishikawa; individualCount: 35; sex: 19 males, 16 females; lifeStage: adult; otherCatalogNumbers: 2014-00279 | 2014-00280 | 2014-00281 | 2014-00282 | 2014-00283 | 2014-00284 | 2014-00285 | 2014-00286 | 2014-00287 | 2014-00288 | 2014-00289 | 2014-00290 | 2014-00291 | 2014-00292 | 2014-00293 | 2014-00294 | 2014-00295 | 2014-00296 | 2014-00297 | 2014-00298 | 2014-00299 | 2014-00300 | 2014-00301 | 2014-00302 | 2014-00303 | 2014-00304 | 2014-00305 | 2014-00306 | 2014-00307 | 2014-00308 | 2014-00309 | 2014-00310 | 2014-00311 | 2014-00312 | 2014-00313; **Taxon:** namePublishedIn: 1906; kingdom: Animalia; phylum: Arthropoda; class: Insecta; order: Hemiptera; family: Miridae; genus: Pseudophylus; specificEpithet: flavipes; scientificNameAuthorship: Nitobe; **Location:** country: Japan; stateProvince: Tokyo; municipality: Meguro-ku; locality: The University of Tokyo Campus, Komaba.; minimumElevationInMeters: 31; maximumElevationInMeters: 39; decimalLatitude: 35.66006; decimalLongitude: 139.68521; geodeticDatum: WGS84; **Identification:** identifiedBy: T. Ishikawa; dateIdentified: 2013; **Event:** samplingProtocol: net sweeping; eventDate: 2013-05-01/2013-05-04; **Record Level:** institutionCode: KMUT; collectionCode: IC
- **Type status:** Other material. **Occurrence:** recordedBy: T. Ishikawa; individualCount: 1; sex: 1 male; lifeStage: adult; otherCatalogNumbers: 2014-00314; **Taxon:** namePublishedIn: 1906; kingdom: Animalia; phylum: Arthropoda; class: Insecta; order: Hemiptera; family: Miridae; genus: Pseudophylus; specificEpithet: flavipes; scientificNameAuthorship: Nitobe; **Location:** country: Japan; stateProvince: Tokyo; municipality: Meguro-ku; locality: The University of Tokyo Campus, Komaba.; minimumElevationInMeters: 31; maximumElevationInMeters: 39; decimalLatitude: 35.66006; decimalLongitude: 139.68521; geodeticDatum: WGS84; **Identification:** identifiedBy: T. Ishikawa; dateIdentified: 2013; **Event:** samplingProtocol: net sweeping; eventDate: 2013-05-12; **Record Level:** institutionCode: KMUT; collectionCode: IC
- **Type status:** Other material. **Occurrence:** recordedBy: T. Ishikawa; individualCount: 1; sex: 1 female; lifeStage: adult; otherCatalogNumbers: 2014-00315; **Taxon:** namePublishedIn: 1906; kingdom: Animalia; phylum: Arthropoda; class: Insecta; order: Hemiptera; family: Miridae; genus: Pseudophylus; specificEpithet: flavipes; scientificNameAuthorship: Nitobe; **Location:** country: Japan; stateProvince: Tokyo; municipality: Meguro-ku; locality: The University of Tokyo Campus, Komaba.; minimumElevationInMeters: 31; maximumElevationInMeters: 39; decimalLatitude: 35.66006; decimalLongitude: 139.68521; geodeticDatum: WGS84; **Identification:** identifiedBy: T. Ishikawa; dateIdentified: 2013; **Event:** samplingProtocol: net sweeping; eventDate: 2013-05-18; **Record Level:** institutionCode: KMUT; collectionCode: IC
- **Type status:** Other material. **Occurrence:** recordedBy: T. Ishikawa; individualCount: 1; sex: 1 female; lifeStage: adult; otherCatalogNumbers: 2014-00316; **Taxon:** namePublishedIn: 1906; kingdom: Animalia; phylum: Arthropoda; class: Insecta; order: Hemiptera; family: Miridae; genus: Pseudophylus; specificEpithet: flavipes; scientificNameAuthorship: Nitobe; **Location:** country: Japan; stateProvince: Tokyo; municipality: Meguro-ku; locality: The University of Tokyo Campus, Komaba.; minimumElevationInMeters: 31; maximumElevationInMeters: 39; decimalLatitude: 35.66006; decimalLongitude: 139.68521; geodeticDatum: WGS84; **Identification:** identifiedBy: T. Ishikawa; dateIdentified: 2013; **Event:** samplingProtocol: net sweeping; eventDate: 2013-05-25; **Record Level:** institutionCode: KMUT; collectionCode: IC

#### Sejanus komabanus

Yasunaga, Ishikawa et Ito, 2013

##### Materials

- **Type status:** Other material. **Occurrence:** recordedBy: T. Ishikawa; individualCount: 7; sex: 7 females; lifeStage: adult; otherCatalogNumbers: 2014-00317 | 2014-00318 | 2014-00319 | 2014-00321 | 2014-00323 | 2014-00324 | 2014-00326; **Taxon:** namePublishedIn: 2013; kingdom: Animalia; phylum: Arthropoda; class: Insecta; order: Hemiptera; family: Miridae; genus: Sejanus; specificEpithet: komabanus; scientificNameAuthorship: Yasunaga, Ishikawa et Ito; **Location:** country: Japan; stateProvince: Tokyo; municipality: Meguro-ku; locality: The University of Tokyo Campus, Komaba.; minimumElevationInMeters: 31; maximumElevationInMeters: 39; decimalLatitude: 35.66006; decimalLongitude: 139.68521; geodeticDatum: WGS84; **Identification:** identifiedBy: T. Ishikawa; dateIdentified: 2013; **Event:** samplingProtocol: net sweeping; eventDate: 2013-06-15/2013-06-18; **Record Level:** institutionCode: KMUT; collectionCode: IC

##### Notes

Recently described as a new species from Komaba Campus (Yasunaga et al. 2013).

#### Stethoconus japonicus

Schumacher, 1917

##### Materials

- **Type status:** Other material. **Occurrence:** recordedBy: T. Ishikawa; individualCount: 2; sex: 2 females; lifeStage: adult; otherCatalogNumbers: 2014-00372 | 2014-00373; **Taxon:** namePublishedIn: 1917; kingdom: Animalia; phylum: Arthropoda; class: Insecta; order: Hemiptera; family: Miridae; genus: Stethoconus; specificEpithet: japonicus; scientificNameAuthorship: Schumacher; **Location:** country: Japan; stateProvince: Tokyo; municipality: Meguro-ku; locality: The University of Tokyo Campus, Komaba.; minimumElevationInMeters: 31; maximumElevationInMeters: 39; decimalLatitude: 35.66006; decimalLongitude: 139.68521; geodeticDatum: WGS84; **Identification:** identifiedBy: T. Ishikawa; dateIdentified: 2013; **Event:** samplingProtocol: light trap; eventDate: 2013-08-13; **Record Level:** institutionCode: KMUT; collectionCode: IC
- **Type status:** Other material. **Occurrence:** recordedBy: T. Ishikawa; individualCount: 1; sex: 1 male; lifeStage: adult; otherCatalogNumbers: 2014-00374; **Taxon:** namePublishedIn: 1917; kingdom: Animalia; phylum: Arthropoda; class: Insecta; order: Hemiptera; family: Miridae; genus: Stethoconus; specificEpithet: japonicus; scientificNameAuthorship: Schumacher; **Location:** country: Japan; stateProvince: Tokyo; municipality: Meguro-ku; locality: The University of Tokyo Campus, Komaba.; minimumElevationInMeters: 31; maximumElevationInMeters: 39; decimalLatitude: 35.66006; decimalLongitude: 139.68521; geodeticDatum: WGS84; **Identification:** identifiedBy: T. Ishikawa; dateIdentified: 2013; **Event:** samplingProtocol: net sweeping; eventDate: 2013-08-15; **Record Level:** institutionCode: KMUT; collectionCode: IC

#### Taylorilygus apicalis

(Fieber, 1861)

##### Materials

- **Type status:** Other material. **Occurrence:** recordedBy: T. Ishikawa; individualCount: 15; sex: 2 males, 13 females; lifeStage: adult; otherCatalogNumbers: 2014-00619 | 2014-00620 | 2014-00621 | 2014-00622 | 2014-00623 | 2014-00624 | 2014-00625 | 2014-00626 | 2014-00627 | 2014-00628 | 2014-00629 | 2014-00630 | 2014-00631 | 2014-00632 | 2014-00633; **Taxon:** namePublishedIn: 1861; kingdom: Animalia; phylum: Arthropoda; class: Insecta; order: Hemiptera; family: Miridae; genus: Taylorilygus; specificEpithet: apicalis; scientificNameAuthorship: Fieber; **Location:** country: Japan; stateProvince: Tokyo; municipality: Meguro-ku; locality: The University of Tokyo Campus, Komaba.; minimumElevationInMeters: 31; maximumElevationInMeters: 39; decimalLatitude: 35.66006; decimalLongitude: 139.68521; geodeticDatum: WGS84; **Identification:** identifiedBy: T. Ishikawa; dateIdentified: 2013; **Event:** samplingProtocol: net sweeping; eventDate: 2013-10-30; **Record Level:** institutionCode: KMUT; collectionCode: IC

#### Termatophylum hikosanum

Miyamoto, 1965

##### Materials

- **Type status:** Other material. **Occurrence:** recordedBy: T. Ishikawa; individualCount: 1; sex: 1 male; lifeStage: adult; otherCatalogNumbers: 2014-00375; **Taxon:** namePublishedIn: 1965; kingdom: Animalia; phylum: Arthropoda; class: Insecta; order: Hemiptera; family: Miridae; genus: Termatophylum; specificEpithet: hikosanum; scientificNameAuthorship: Miyamoto; **Location:** country: Japan; stateProvince: Tokyo; municipality: Meguro-ku; locality: The University of Tokyo Campus, Komaba.; minimumElevationInMeters: 31; maximumElevationInMeters: 39; decimalLatitude: 35.66006; decimalLongitude: 139.68521; geodeticDatum: WGS84; **Identification:** identifiedBy: T. Ishikawa; dateIdentified: 2013; **Event:** samplingProtocol: net sweeping; eventDate: 2013-06-18; **Record Level:** institutionCode: KMUT; collectionCode: IC
- **Type status:** Other material. **Occurrence:** recordedBy: T. Ishikawa; individualCount: 1; sex: 1 female; lifeStage: adult; otherCatalogNumbers: 2014-00376; **Taxon:** namePublishedIn: 1965; kingdom: Animalia; phylum: Arthropoda; class: Insecta; order: Hemiptera; family: Miridae; genus: Termatophylum; specificEpithet: hikosanum; scientificNameAuthorship: Miyamoto; **Location:** country: Japan; stateProvince: Tokyo; municipality: Meguro-ku; locality: The University of Tokyo Campus, Komaba.; minimumElevationInMeters: 31; maximumElevationInMeters: 39; decimalLatitude: 35.66006; decimalLongitude: 139.68521; geodeticDatum: WGS84; **Identification:** identifiedBy: T. Ishikawa; dateIdentified: 2013; **Event:** samplingProtocol: net sweeping; eventDate: 2013-10-30; **Record Level:** institutionCode: KMUT; collectionCode: IC

#### Trigonotylus caelestialium

(Kirkaldy, 1902)

##### Materials

- **Type status:** Other material. **Occurrence:** recordedBy: T. Ishikawa; individualCount: 23; sex: 8 males, 15 females; lifeStage: adult; otherCatalogNumbers: 2014-00635 | 2014-00636 | 2014-00637 | 2014-00638 | 2014-00639 | 2014-00640 | 2014-00641 | 2014-00642 | 2014-00643 | 2014-00644 | 2014-00645 | 2014-00646 | 2014-00647 | 2014-00648 | 2014-00649 | 2014-00650 | 2014-00651 | 2014-00652 | 2014-00653 | 2014-00654 | 2014-00655 | 2014-00656 | 2014-00657; **Taxon:** namePublishedIn: 1902; kingdom: Animalia; phylum: Arthropoda; class: Insecta; order: Hemiptera; family: Miridae; genus: Trigonotylus; specificEpithet: caelestialium; scientificNameAuthorship: Kirkaldy; **Location:** country: Japan; stateProvince: Tokyo; municipality: Meguro-ku; locality: The University of Tokyo Campus, Komaba.; minimumElevationInMeters: 31; maximumElevationInMeters: 39; decimalLatitude: 35.66006; decimalLongitude: 139.68521; geodeticDatum: WGS84; **Identification:** identifiedBy: T. Ishikawa; dateIdentified: 2013; **Event:** samplingProtocol: net sweeping; eventDate: 2013-05-12; **Record Level:** institutionCode: KMUT; collectionCode: IC
- **Type status:** Other material. **Occurrence:** recordedBy: T. Ishikawa; individualCount: 2; sex: 2 males; lifeStage: adult; otherCatalogNumbers: 2014-00658 | 2014-00659; **Taxon:** namePublishedIn: 1902; kingdom: Animalia; phylum: Arthropoda; class: Insecta; order: Hemiptera; family: Miridae; genus: Trigonotylus; specificEpithet: caelestialium; scientificNameAuthorship: Kirkaldy; **Location:** country: Japan; stateProvince: Tokyo; municipality: Meguro-ku; locality: The University of Tokyo Campus, Komaba.; minimumElevationInMeters: 31; maximumElevationInMeters: 39; decimalLatitude: 35.66006; decimalLongitude: 139.68521; geodeticDatum: WGS84; **Identification:** identifiedBy: T. Ishikawa; dateIdentified: 2013; **Event:** samplingProtocol: light trap; eventDate: 2013-05-14; **Record Level:** institutionCode: KMUT; collectionCode: IC
- **Type status:** Other material. **Occurrence:** recordedBy: T. Ishikawa; individualCount: 8; sex: 1 male, 7 females; lifeStage: adult; otherCatalogNumbers: 2014-00660 | 2014-00661 | 2014-00662 | 2014-00663 | 2014-00664 | 2014-00665 | 2014-00666 | 2014-00667; **Taxon:** namePublishedIn: 1902; kingdom: Animalia; phylum: Arthropoda; class: Insecta; order: Hemiptera; family: Miridae; genus: Trigonotylus; specificEpithet: caelestialium; scientificNameAuthorship: Kirkaldy; **Location:** country: Japan; stateProvince: Tokyo; municipality: Meguro-ku; locality: The University of Tokyo Campus, Komaba.; minimumElevationInMeters: 31; maximumElevationInMeters: 39; decimalLatitude: 35.66006; decimalLongitude: 139.68521; geodeticDatum: WGS84; **Identification:** identifiedBy: T. Ishikawa; dateIdentified: 2013; **Event:** samplingProtocol: net sweeping; eventDate: 2013-05-19; **Record Level:** institutionCode: KMUT; collectionCode: IC
- **Type status:** Other material. **Occurrence:** recordedBy: T. Ishikawa; individualCount: 1; sex: 1 male; lifeStage: adult; otherCatalogNumbers: 2014-00668; **Taxon:** namePublishedIn: 1902; kingdom: Animalia; phylum: Arthropoda; class: Insecta; order: Hemiptera; family: Miridae; genus: Trigonotylus; specificEpithet: caelestialium; scientificNameAuthorship: Kirkaldy; **Location:** country: Japan; stateProvince: Tokyo; municipality: Meguro-ku; locality: The University of Tokyo Campus, Komaba.; minimumElevationInMeters: 31; maximumElevationInMeters: 39; decimalLatitude: 35.66006; decimalLongitude: 139.68521; geodeticDatum: WGS84; **Identification:** identifiedBy: T. Ishikawa; dateIdentified: 2013; **Event:** samplingProtocol: light trap; eventDate: 2013-08-02; **Record Level:** institutionCode: KMUT; collectionCode: IC
- **Type status:** Other material. **Occurrence:** recordedBy: T. Ishikawa; individualCount: 15; sex: 5 males, 10 females; lifeStage: adult; otherCatalogNumbers: 2014-00669 | 2014-00670 | 2014-00671 | 2014-00672 | 2014-00673 | 2014-00674 | 2014-00675 | 2014-00676 | 2014-00677 | 2014-00678 | 2014-00679 | 2014-00680 | 2014-00681 | 2014-00682 | 2014-00683; **Taxon:** namePublishedIn: 1902; kingdom: Animalia; phylum: Arthropoda; class: Insecta; order: Hemiptera; family: Miridae; genus: Trigonotylus; specificEpithet: caelestialium; scientificNameAuthorship: Kirkaldy; **Location:** country: Japan; stateProvince: Tokyo; municipality: Meguro-ku; locality: The University of Tokyo Campus, Komaba.; minimumElevationInMeters: 31; maximumElevationInMeters: 39; decimalLatitude: 35.66006; decimalLongitude: 139.68521; geodeticDatum: WGS84; **Identification:** identifiedBy: T. Ishikawa; dateIdentified: 2013; **Event:** samplingProtocol: net sweeping; eventDate: 2013-08-15; **Record Level:** institutionCode: KMUT; collectionCode: IC
- **Type status:** Other material. **Occurrence:** recordedBy: T. Ishikawa; individualCount: 1; sex: 1 female; lifeStage: adult; otherCatalogNumbers: 2014-00684; **Taxon:** namePublishedIn: 1902; kingdom: Animalia; phylum: Arthropoda; class: Insecta; order: Hemiptera; family: Miridae; genus: Trigonotylus; specificEpithet: caelestialium; scientificNameAuthorship: Kirkaldy; **Location:** country: Japan; stateProvince: Tokyo; municipality: Meguro-ku; locality: The University of Tokyo Campus, Komaba.; minimumElevationInMeters: 31; maximumElevationInMeters: 39; decimalLatitude: 35.66006; decimalLongitude: 139.68521; geodeticDatum: WGS84; **Identification:** identifiedBy: T. Ishikawa; dateIdentified: 2013; **Event:** samplingProtocol: net sweeping; eventDate: 2013-10-30; **Record Level:** institutionCode: KMUT; collectionCode: IC

#### Yamatolygus sp.

##### Materials

- **Type status:** Other material. **Occurrence:** recordedBy: T. Ishikawa; individualCount: 1; sex: 1 male; lifeStage: adult; otherCatalogNumbers: 2014-00634; **Taxon:** kingdom: Animalia; phylum: Arthropoda; class: Insecta; order: Hemiptera; family: Miridae; genus: Yamatolygus; specificEpithet: sp.; **Location:** country: Japan; stateProvince: Tokyo; municipality: Meguro-ku; locality: The University of Tokyo Campus, Komaba.; minimumElevationInMeters: 31; maximumElevationInMeters: 39; decimalLatitude: 35.66006; decimalLongitude: 139.68521; geodeticDatum: WGS84; **Identification:** identifiedBy: T. Ishikawa; dateIdentified: 2013; **Event:** samplingProtocol: net sweeping; eventDate: 2013-10-30; **Record Level:** institutionCode: KMUT; collectionCode: IC

##### Notes

Probably belongs to an undescribed species.

#### Zanchius tarasovi

Kerzhner, 1988

##### Materials

- **Type status:** Other material. **Occurrence:** recordedBy: T. Ishikawa; individualCount: 13; sex: 7 males, 6 females; lifeStage: adult; otherCatalogNumbers: 2014-00156 | 2014-00157 | 2014-00158 | 2014-00159 | 2014-00160 | 2014-00161 | 2014-00162 | 2014-00163 | 2014-00164 | 2014-00165 | 2014-00166 | 2014-00167 | 2014-00168; **Taxon:** namePublishedIn: 1988; kingdom: Animalia; phylum: Arthropoda; class: Insecta; order: Hemiptera; family: Miridae; genus: Zanchius; specificEpithet: tarasovi; scientificNameAuthorship: Kerzhner; **Location:** country: Japan; stateProvince: Tokyo; municipality: Meguro-ku; locality: The University of Tokyo Campus, Komaba.; minimumElevationInMeters: 31; maximumElevationInMeters: 39; decimalLatitude: 35.66006; decimalLongitude: 139.68521; geodeticDatum: WGS84; **Identification:** identifiedBy: T. Ishikawa; dateIdentified: 2013; **Event:** samplingProtocol: net sweeping; eventDate: 2013-08-15/2013-08-19; **Record Level:** institutionCode: KMUT; collectionCode: IC
- **Type status:** Other material. **Occurrence:** recordedBy: T. Ishikawa; individualCount: 3; sex: 1 male, 2 females; lifeStage: adult; otherCatalogNumbers: 2014-00169 | 2014-00170 | 2014-00171; **Taxon:** namePublishedIn: 1988; kingdom: Animalia; phylum: Arthropoda; class: Insecta; order: Hemiptera; family: Miridae; genus: Zanchius; specificEpithet: tarasovi; scientificNameAuthorship: Kerzhner; **Location:** country: Japan; stateProvince: Tokyo; municipality: Meguro-ku; locality: The University of Tokyo Campus, Komaba.; minimumElevationInMeters: 31; maximumElevationInMeters: 39; decimalLatitude: 35.66006; decimalLongitude: 139.68521; geodeticDatum: WGS84; **Identification:** identifiedBy: T. Ishikawa; dateIdentified: 2013; **Event:** samplingProtocol: net sweeping; eventDate: 2013-10-30; **Record Level:** institutionCode: KMUT; collectionCode: IC

##### Notes

First record in Tokyo.

#### Nabidae

A. Costa, 1853

#### Nabis kinbergii

Reuter, 1872

##### Materials

- **Type status:** Other material. **Occurrence:** recordedBy: T. Ishikawa; individualCount: 1; sex: 1 male; lifeStage: adult; otherCatalogNumbers: 2014-01010; **Taxon:** namePublishedIn: 1872; kingdom: Animalia; phylum: Arthropoda; class: Insecta; order: Hemiptera; family: Nabidae; genus: Nabis; specificEpithet: kinbergii; scientificNameAuthorship: Reuter; **Location:** country: Japan; stateProvince: Tokyo; municipality: Meguro-ku; locality: The University of Tokyo Campus, Komaba.; minimumElevationInMeters: 31; maximumElevationInMeters: 39; decimalLatitude: 35.66006; decimalLongitude: 139.68521; geodeticDatum: WGS84; **Identification:** identifiedBy: T. Ishikawa; dateIdentified: 2013; **Event:** samplingProtocol: net sweeping; eventDate: 2013-04-28; **Record Level:** institutionCode: KMUT; collectionCode: IC
- **Type status:** Other material. **Occurrence:** recordedBy: T. Ishikawa; individualCount: 2; sex: 2 females; lifeStage: adult; otherCatalogNumbers: 2014-01011 | 2014-01012; **Taxon:** namePublishedIn: 1872; kingdom: Animalia; phylum: Arthropoda; class: Insecta; order: Hemiptera; family: Nabidae; genus: Nabis; specificEpithet: kinbergii; scientificNameAuthorship: Reuter; **Location:** country: Japan; stateProvince: Tokyo; municipality: Meguro-ku; locality: The University of Tokyo Campus, Komaba.; minimumElevationInMeters: 31; maximumElevationInMeters: 39; decimalLatitude: 35.66006; decimalLongitude: 139.68521; geodeticDatum: WGS84; **Identification:** identifiedBy: T. Ishikawa; dateIdentified: 2013; **Event:** samplingProtocol: net sweeping; eventDate: 2013-08-15; **Record Level:** institutionCode: KMUT; collectionCode: IC
- **Type status:** Other material. **Occurrence:** recordedBy: T. Ishikawa; individualCount: 6; sex: 3 males, 3 females; lifeStage: adult; otherCatalogNumbers: 2014-01013 | 2014-01014 | 2014-01015 | 2014-01016 | 2014-01017 | 2014-01018; **Taxon:** namePublishedIn: 1872; kingdom: Animalia; phylum: Arthropoda; class: Insecta; order: Hemiptera; family: Nabidae; genus: Nabis; specificEpithet: kinbergii; scientificNameAuthorship: Reuter; **Location:** country: Japan; stateProvince: Tokyo; municipality: Meguro-ku; locality: The University of Tokyo Campus, Komaba.; minimumElevationInMeters: 31; maximumElevationInMeters: 39; decimalLatitude: 35.66006; decimalLongitude: 139.68521; geodeticDatum: WGS84; **Identification:** identifiedBy: T. Ishikawa; dateIdentified: 2013; **Event:** samplingProtocol: net sweeping; eventDate: 2013-09-06; **Record Level:** institutionCode: KMUT; collectionCode: IC
- **Type status:** Other material. **Occurrence:** recordedBy: T. Ishikawa; individualCount: 1; sex: 1 female; lifeStage: adult; otherCatalogNumbers: 2014-01019; **Taxon:** namePublishedIn: 1872; kingdom: Animalia; phylum: Arthropoda; class: Insecta; order: Hemiptera; family: Nabidae; genus: Nabis; specificEpithet: kinbergii; scientificNameAuthorship: Reuter; **Location:** country: Japan; stateProvince: Tokyo; municipality: Meguro-ku; locality: The University of Tokyo Campus, Komaba.; minimumElevationInMeters: 31; maximumElevationInMeters: 39; decimalLatitude: 35.66006; decimalLongitude: 139.68521; geodeticDatum: WGS84; **Identification:** identifiedBy: T. Ishikawa; dateIdentified: 2013; **Event:** samplingProtocol: net sweeping; eventDate: 2013-10-30; **Record Level:** institutionCode: KMUT; collectionCode: IC
- **Type status:** Other material. **Occurrence:** recordedBy: T. Kato; individualCount: 1; sex: 1 female; lifeStage: adult; otherCatalogNumbers: 2014-01020; **Taxon:** namePublishedIn: 1872; kingdom: Animalia; phylum: Arthropoda; class: Insecta; order: Hemiptera; family: Nabidae; genus: Nabis; specificEpithet: kinbergii; scientificNameAuthorship: Reuter; **Location:** country: Japan; stateProvince: Tokyo; municipality: Meguro-ku; locality: The University of Tokyo Campus, Komaba.; minimumElevationInMeters: 31; maximumElevationInMeters: 39; decimalLatitude: 35.66006; decimalLongitude: 139.68521; geodeticDatum: WGS84; **Identification:** identifiedBy: T. Ishikawa; dateIdentified: 2013; **Event:** samplingProtocol: net sweeping; eventDate: 2013-11-03; **Record Level:** institutionCode: KMUT; collectionCode: IC

#### Anthocoridae

Fieber, 1836

#### Amphiareus obscuriceps

(Poppius, 1909)

##### Materials

- **Type status:** Other material. **Occurrence:** recordedBy: T. Ishikawa; individualCount: 13; sex: 3 males, 10 females; lifeStage: adult; otherCatalogNumbers: 2014-00813 | 2014-00814 | 2014-00815 | 2014-00816 | 2014-00817 | 2014-00818 | 2014-00819 | 2014-00820 | 2014-00821 | 2014-00822 | 2014-00823 | 2014-00824 | 2014-00825; **Taxon:** namePublishedIn: 1909; kingdom: Animalia; phylum: Arthropoda; class: Insecta; order: Hemiptera; family: Anthocoridae; genus: Amphiareus; specificEpithet: obscuriceps; scientificNameAuthorship: Poppius; **Location:** country: Japan; stateProvince: Tokyo; municipality: Meguro-ku; locality: The University of Tokyo Campus, Komaba.; minimumElevationInMeters: 31; maximumElevationInMeters: 39; decimalLatitude: 35.66006; decimalLongitude: 139.68521; geodeticDatum: WGS84; **Identification:** identifiedBy: T. Ishikawa; dateIdentified: 2013; **Event:** samplingProtocol: net sweeping; eventDate: 2013-06-16; **Record Level:** institutionCode: KMUT; collectionCode: IC
- **Type status:** Other material. **Occurrence:** recordedBy: T. Ishikawa; individualCount: 9; sex: 3 males, 6 females; lifeStage: adult; otherCatalogNumbers: 2014-00826 | 2014-00827 | 2014-00828 | 2014-00829 | 2014-00830 | 2014-00831 | 2014-00832 | 2014-00833 | 2014-00834; **Taxon:** namePublishedIn: 1909; kingdom: Animalia; phylum: Arthropoda; class: Insecta; order: Hemiptera; family: Anthocoridae; genus: Amphiareus; specificEpithet: obscuriceps; scientificNameAuthorship: Poppius; **Location:** country: Japan; stateProvince: Tokyo; municipality: Meguro-ku; locality: The University of Tokyo Campus, Komaba.; minimumElevationInMeters: 31; maximumElevationInMeters: 39; decimalLatitude: 35.66006; decimalLongitude: 139.68521; geodeticDatum: WGS84; **Identification:** identifiedBy: T. Ishikawa; dateIdentified: 2013; **Event:** samplingProtocol: light trap; eventDate: 2013-08-02; **Record Level:** institutionCode: KMUT; collectionCode: IC
- **Type status:** Other material. **Occurrence:** recordedBy: T. Ishikawa; individualCount: 3; sex: 1 male, 2 females; lifeStage: adult; otherCatalogNumbers: 2014-00835 | 2014-00836 | 2014-00837; **Taxon:** namePublishedIn: 1909; kingdom: Animalia; phylum: Arthropoda; class: Insecta; order: Hemiptera; family: Anthocoridae; genus: Amphiareus; specificEpithet: obscuriceps; scientificNameAuthorship: Poppius; **Location:** country: Japan; stateProvince: Tokyo; municipality: Meguro-ku; locality: The University of Tokyo Campus, Komaba.; minimumElevationInMeters: 31; maximumElevationInMeters: 39; decimalLatitude: 35.66006; decimalLongitude: 139.68521; geodeticDatum: WGS84; **Identification:** identifiedBy: T. Ishikawa; dateIdentified: 2013; **Event:** samplingProtocol: light trap; eventDate: 2013-08-13; **Record Level:** institutionCode: KMUT; collectionCode: IC
- **Type status:** Other material. **Occurrence:** recordedBy: T. Ishikawa; individualCount: 2; sex: 2 females; lifeStage: adult; otherCatalogNumbers: 2014-00838 | 2014-00839; **Taxon:** namePublishedIn: 1909; kingdom: Animalia; phylum: Arthropoda; class: Insecta; order: Hemiptera; family: Anthocoridae; genus: Amphiareus; specificEpithet: obscuriceps; scientificNameAuthorship: Poppius; **Location:** country: Japan; stateProvince: Tokyo; municipality: Meguro-ku; locality: The University of Tokyo Campus, Komaba.; minimumElevationInMeters: 31; maximumElevationInMeters: 39; decimalLatitude: 35.66006; decimalLongitude: 139.68521; geodeticDatum: WGS84; **Identification:** identifiedBy: T. Ishikawa; dateIdentified: 2013; **Event:** samplingProtocol: net sweeping; eventDate: 2013-08-18/2013-08-19; **Record Level:** institutionCode: KMUT; collectionCode: IC
- **Type status:** Other material. **Occurrence:** recordedBy: T. Ishikawa; individualCount: 1; sex: 1 male; lifeStage: adult; otherCatalogNumbers: 2014-00840; **Taxon:** namePublishedIn: 1909; kingdom: Animalia; phylum: Arthropoda; class: Insecta; order: Hemiptera; family: Anthocoridae; genus: Amphiareus; specificEpithet: obscuriceps; scientificNameAuthorship: Poppius; **Location:** country: Japan; stateProvince: Tokyo; municipality: Meguro-ku; locality: The University of Tokyo Campus, Komaba.; minimumElevationInMeters: 31; maximumElevationInMeters: 39; decimalLatitude: 35.66006; decimalLongitude: 139.68521; geodeticDatum: WGS84; **Identification:** identifiedBy: T. Ishikawa; dateIdentified: 2013; **Event:** samplingProtocol: net sweeping; eventDate: 2013-10-30; **Record Level:** institutionCode: KMUT; collectionCode: IC

#### Cardiastethus pygmaeus

Poppius, 1913

##### Materials

- **Type status:** Other material. **Occurrence:** recordedBy: T. Ishikawa; individualCount: 2; sex: 1 male, 1 female; lifeStage: adult; otherCatalogNumbers: 2014-00841 | 2014-00842; **Taxon:** namePublishedIn: 1915; kingdom: Animalia; phylum: Arthropoda; class: Insecta; order: Hemiptera; family: Anthocoridae; genus: Cardiastethus; specificEpithet: pygmaeus; scientificNameAuthorship: Poppius; **Location:** country: Japan; stateProvince: Tokyo; municipality: Meguro-ku; locality: The University of Tokyo Campus, Komaba.; minimumElevationInMeters: 31; maximumElevationInMeters: 39; decimalLatitude: 35.66006; decimalLongitude: 139.68521; geodeticDatum: WGS84; **Identification:** identifiedBy: T. Ishikawa; dateIdentified: 2013; **Event:** samplingProtocol: light trap; eventDate: 2013-08-02; **Record Level:** institutionCode: KMUT; collectionCode: IC
- **Type status:** Other material. **Occurrence:** recordedBy: T. Ishikawa; individualCount: 2; sex: 2 males; lifeStage: adult; otherCatalogNumbers: 2014-00843 | 2014-00844; **Taxon:** namePublishedIn: 1915; kingdom: Animalia; phylum: Arthropoda; class: Insecta; order: Hemiptera; family: Anthocoridae; genus: Cardiastethus; specificEpithet: pygmaeus; scientificNameAuthorship: Poppius; **Location:** country: Japan; stateProvince: Tokyo; municipality: Meguro-ku; locality: The University of Tokyo Campus, Komaba.; minimumElevationInMeters: 31; maximumElevationInMeters: 39; decimalLatitude: 35.66006; decimalLongitude: 139.68521; geodeticDatum: WGS84; **Identification:** identifiedBy: T. Ishikawa; dateIdentified: 2013; **Event:** samplingProtocol: light trap; eventDate: 2013-08-13; **Record Level:** institutionCode: KMUT; collectionCode: IC
- **Type status:** Other material. **Occurrence:** recordedBy: T. Ishikawa; individualCount: 3; sex: 3 females; lifeStage: adult; otherCatalogNumbers: 2014-00845 | 2014-00846 | 2014-00847; **Taxon:** namePublishedIn: 1915; kingdom: Animalia; phylum: Arthropoda; class: Insecta; order: Hemiptera; family: Anthocoridae; genus: Cardiastethus; specificEpithet: pygmaeus; scientificNameAuthorship: Poppius; **Location:** country: Japan; stateProvince: Tokyo; municipality: Meguro-ku; locality: The University of Tokyo Campus, Komaba.; minimumElevationInMeters: 31; maximumElevationInMeters: 39; decimalLatitude: 35.66006; decimalLongitude: 139.68521; geodeticDatum: WGS84; **Identification:** identifiedBy: T. Ishikawa; dateIdentified: 2013; **Event:** samplingProtocol: net sweeping; eventDate: 2013-08-15/2013-08-18; **Record Level:** institutionCode: KMUT; collectionCode: IC

#### Orius minutus

(Linnaeus, 1758)

##### Materials

- **Type status:** Other material. **Occurrence:** recordedBy: T. Ishikawa; individualCount: 1; sex: 1 female; lifeStage: adult; otherCatalogNumbers: 2014-00685; **Taxon:** namePublishedIn: 1758; kingdom: Animalia; phylum: Arthropoda; class: Insecta; order: Hemiptera; family: Anthocoridae; genus: Orius; specificEpithet: minutus; scientificNameAuthorship: Linnaeus; **Location:** country: Japan; stateProvince: Tokyo; municipality: Meguro-ku; locality: The University of Tokyo Campus, Komaba.; minimumElevationInMeters: 31; maximumElevationInMeters: 39; decimalLatitude: 35.66006; decimalLongitude: 139.68521; geodeticDatum: WGS84; **Identification:** identifiedBy: T. Ishikawa; dateIdentified: 2013; **Event:** samplingProtocol: light trap; eventDate: 2013-05-14; **Record Level:** institutionCode: KMUT; collectionCode: IC
- **Type status:** Other material. **Occurrence:** recordedBy: T. Ishikawa; individualCount: 1; sex: 1 female; lifeStage: adult; otherCatalogNumbers: 2014-00686; **Taxon:** namePublishedIn: 1758; kingdom: Animalia; phylum: Arthropoda; class: Insecta; order: Hemiptera; family: Anthocoridae; genus: Orius; specificEpithet: minutus; scientificNameAuthorship: Linnaeus; **Location:** country: Japan; stateProvince: Tokyo; municipality: Meguro-ku; locality: The University of Tokyo Campus, Komaba.; minimumElevationInMeters: 31; maximumElevationInMeters: 39; decimalLatitude: 35.66006; decimalLongitude: 139.68521; geodeticDatum: WGS84; **Identification:** identifiedBy: T. Ishikawa; dateIdentified: 2013; **Event:** samplingProtocol: net sweeping; eventDate: 2013-05-25; **Record Level:** institutionCode: KMUT; collectionCode: IC
- **Type status:** Other material. **Occurrence:** recordedBy: T. Ishikawa; individualCount: 52; sex: 10 males, 42 females; lifeStage: adult; otherCatalogNumbers: 2014-00687 | 2014-00688 | 2014-00689 | 2014-00690 | 2014-00691 | 2014-00692 | 2014-00693 | 2014-00694 | 2014-00695 | 2014-00696 | 2014-00697 | 2014-00698 | 2014-00699 | 2014-00700 | 2014-00701 | 2014-00702 | 2014-00703 | 2014-00704 | 2014-00705 | 2014-00706 | 2014-00707 | 2014-00708 | 2014-00709 | 2014-00710 | 2014-00711 | 2014-00712 | 2014-00713 | 2014-00714 | 2014-00715 | 2014-00716 | 2014-00717 | 2014-00718 | 2014-00719 | 2014-00720 | 2014-00721 | 2014-00722 | 2014-00723 | 2014-00724 | 2014-00725 | 2014-00726 | 2014-00727 | 2014-00728 | 2014-00729 | 2014-00730 | 2014-00731 | 2014-00732 | 2014-00733 | 2014-00734 | 2014-00735 | 2014-00736 | 2014-00737 | 2014-00738; **Taxon:** namePublishedIn: 1758; kingdom: Animalia; phylum: Arthropoda; class: Insecta; order: Hemiptera; family: Anthocoridae; genus: Orius; specificEpithet: minutus; scientificNameAuthorship: Linnaeus; **Location:** country: Japan; stateProvince: Tokyo; municipality: Meguro-ku; locality: The University of Tokyo Campus, Komaba.; minimumElevationInMeters: 31; maximumElevationInMeters: 39; decimalLatitude: 35.66006; decimalLongitude: 139.68521; geodeticDatum: WGS84; **Identification:** identifiedBy: T. Ishikawa; dateIdentified: 2013; **Event:** samplingProtocol: net sweeping; eventDate: 2013-06-15/2013-06-18; **Record Level:** institutionCode: KMUT; collectionCode: IC
- **Type status:** Other material. **Occurrence:** recordedBy: T. Ishikawa; individualCount: 44; sex: 12 males, 32 females; lifeStage: adult; otherCatalogNumbers: 2014-00739 | 2014-00740 | 2014-00741 | 2014-00742 | 2014-00743 | 2014-00744 | 2014-00745 | 2014-00746 | 2014-00747 | 2014-00748 | 2014-00749 | 2014-00750 | 2014-00751 | 2014-00752 | 2014-00753 | 2014-00754 | 2014-00755 | 2014-00756 | 2014-00757 | 2014-00758 | 2014-00759 | 2014-00760 | 2014-00761 | 2014-00762 | 2014-00763 | 2014-00764 | 2014-00765 | 2014-00766 | 2014-00767 | 2014-00768 | 2014-00769 | 2014-00770 | 2014-00771 | 2014-00772 | 2014-00773 | 2014-00774 | 2014-00775 | 2014-00776 | 2014-00777 | 2014-00778 | 2014-00779 | 2014-00780 | 2014-00781 | 2014-00782; **Taxon:** namePublishedIn: 1758; kingdom: Animalia; phylum: Arthropoda; class: Insecta; order: Hemiptera; family: Anthocoridae; genus: Orius; specificEpithet: minutus; scientificNameAuthorship: Linnaeus; **Location:** country: Japan; stateProvince: Tokyo; municipality: Meguro-ku; locality: The University of Tokyo Campus, Komaba.; minimumElevationInMeters: 31; maximumElevationInMeters: 39; decimalLatitude: 35.66006; decimalLongitude: 139.68521; geodeticDatum: WGS84; **Identification:** identifiedBy: T. Ishikawa; dateIdentified: 2013; **Event:** samplingProtocol: net sweeping; eventDate: 2013-06-22/2013-06-28; **Record Level:** institutionCode: KMUT; collectionCode: IC
- **Type status:** Other material. **Occurrence:** recordedBy: T. Ishikawa; individualCount: 5; sex: 5 females; lifeStage: adult; otherCatalogNumbers: 2014-00783 | 2014-00784 | 2014-00785 | 2014-00786 | 2014-00787; **Taxon:** namePublishedIn: 1758; kingdom: Animalia; phylum: Arthropoda; class: Insecta; order: Hemiptera; family: Anthocoridae; genus: Orius; specificEpithet: minutus; scientificNameAuthorship: Linnaeus; **Location:** country: Japan; stateProvince: Tokyo; municipality: Meguro-ku; locality: The University of Tokyo Campus, Komaba.; minimumElevationInMeters: 31; maximumElevationInMeters: 39; decimalLatitude: 35.66006; decimalLongitude: 139.68521; geodeticDatum: WGS84; **Identification:** identifiedBy: T. Ishikawa; dateIdentified: 2013; **Event:** samplingProtocol: net sweeping; eventDate: 2013-09-06; **Record Level:** institutionCode: KMUT; collectionCode: IC
- **Type status:** Other material. **Occurrence:** recordedBy: T. Ishikawa; individualCount: 9; sex: 3 males, 6 females; lifeStage: adult; otherCatalogNumbers: 2014-00788 | 2014-00789 | 2014-00790 | 2014-00791 | 2014-00792 | 2014-00793 | 2014-00794 | 2014-00795 | 2014-00796; **Taxon:** namePublishedIn: 1758; kingdom: Animalia; phylum: Arthropoda; class: Insecta; order: Hemiptera; family: Anthocoridae; genus: Orius; specificEpithet: minutus; scientificNameAuthorship: Linnaeus; **Location:** country: Japan; stateProvince: Tokyo; municipality: Meguro-ku; locality: The University of Tokyo Campus, Komaba.; minimumElevationInMeters: 31; maximumElevationInMeters: 39; decimalLatitude: 35.66006; decimalLongitude: 139.68521; geodeticDatum: WGS84; **Identification:** identifiedBy: T. Ishikawa; dateIdentified: 2013; **Event:** samplingProtocol: net sweeping; eventDate: 2013-10-30; **Record Level:** institutionCode: KMUT; collectionCode: IC
- **Type status:** Other material. **Occurrence:** recordedBy: T. Ishikawa; individualCount: 1; sex: 1 female; lifeStage: adult; otherCatalogNumbers: 2014-00797; **Taxon:** namePublishedIn: 1758; kingdom: Animalia; phylum: Arthropoda; class: Insecta; order: Hemiptera; family: Anthocoridae; genus: Orius; specificEpithet: minutus; scientificNameAuthorship: Linnaeus; **Location:** country: Japan; stateProvince: Tokyo; municipality: Meguro-ku; locality: The University of Tokyo Campus, Komaba.; minimumElevationInMeters: 31; maximumElevationInMeters: 39; decimalLatitude: 35.66006; decimalLongitude: 139.68521; geodeticDatum: WGS84; **Identification:** identifiedBy: T. Ishikawa; dateIdentified: 2014; **Event:** samplingProtocol: net sweeping; eventDate: 2014-01-01; **Record Level:** institutionCode: KMUT; collectionCode: IC

#### Orius nagaii

Yasunaga, 1993

##### Materials

- **Type status:** Other material. **Occurrence:** recordedBy: T. Ishikawa; individualCount: 1; sex: 1 female; lifeStage: adult; otherCatalogNumbers: 2014-00798; **Taxon:** namePublishedIn: 1993; kingdom: Animalia; phylum: Arthropoda; class: Insecta; order: Hemiptera; family: Anthocoridae; genus: Orius; specificEpithet: nagaii; scientificNameAuthorship: Yasunaga; **Location:** country: Japan; stateProvince: Tokyo; municipality: Meguro-ku; locality: The University of Tokyo Campus, Komaba.; minimumElevationInMeters: 31; maximumElevationInMeters: 39; decimalLatitude: 35.66006; decimalLongitude: 139.68521; geodeticDatum: WGS84; **Identification:** identifiedBy: T. Ishikawa; dateIdentified: 2013; **Event:** samplingProtocol: net sweeping; eventDate: 2013-09-06; **Record Level:** institutionCode: KMUT; collectionCode: IC

#### Orius sauteri

(Poppius, 1909)

##### Materials

- **Type status:** Other material. **Occurrence:** recordedBy: T. Ishikawa; individualCount: 7; sex: 6 males, 1 female; lifeStage: adult; otherCatalogNumbers: 2014-00799 | 2014-00800 | 2014-00801 | 2014-00802 | 2014-00803 | 2014-00804 | 2014-00805; **Taxon:** namePublishedIn: 1909; kingdom: Animalia; phylum: Arthropoda; class: Insecta; order: Hemiptera; family: Anthocoridae; genus: Orius; specificEpithet: sauteri; scientificNameAuthorship: Poppius; **Location:** country: Japan; stateProvince: Tokyo; municipality: Meguro-ku; locality: The University of Tokyo Campus, Komaba.; minimumElevationInMeters: 31; maximumElevationInMeters: 39; decimalLatitude: 35.66006; decimalLongitude: 139.68521; geodeticDatum: WGS84; **Identification:** identifiedBy: T. Ishikawa; dateIdentified: 2013; **Event:** samplingProtocol: net sweeping; eventDate: 2013-06-23; **Record Level:** institutionCode: KMUT; collectionCode: IC
- **Type status:** Other material. **Occurrence:** recordedBy: T. Ishikawa; individualCount: 7; sex: 5 males, 2 females; lifeStage: adult; otherCatalogNumbers: 2014-00806 | 2014-00807 | 2014-00808 | 2014-00809 | 2014-00810 | 2014-00811 | 2014-00812; **Taxon:** namePublishedIn: 1909; kingdom: Animalia; phylum: Arthropoda; class: Insecta; order: Hemiptera; family: Anthocoridae; genus: Orius; specificEpithet: sauteri; scientificNameAuthorship: Poppius; **Location:** country: Japan; stateProvince: Tokyo; municipality: Meguro-ku; locality: The University of Tokyo Campus, Komaba.; minimumElevationInMeters: 31; maximumElevationInMeters: 39; decimalLatitude: 35.66006; decimalLongitude: 139.68521; geodeticDatum: WGS84; **Identification:** identifiedBy: T. Ishikawa; dateIdentified: 2013; **Event:** samplingProtocol: net sweeping; eventDate: 2013-09-06; **Record Level:** institutionCode: KMUT; collectionCode: IC

#### Physopleurella armata

Poppius, 1909

##### Materials

- **Type status:** Other material. **Occurrence:** recordedBy: T. Ishikawa; individualCount: 1; sex: 1 female; lifeStage: adult; otherCatalogNumbers: 2014-00848; **Taxon:** namePublishedIn: 1909; kingdom: Animalia; phylum: Arthropoda; class: Insecta; order: Hemiptera; family: Anthocoridae; genus: Physopleurella; specificEpithet: armata; scientificNameAuthorship: Poppius; **Location:** country: Japan; stateProvince: Tokyo; municipality: Meguro-ku; locality: The University of Tokyo Campus, Komaba.; minimumElevationInMeters: 31; maximumElevationInMeters: 39; decimalLatitude: 35.66006; decimalLongitude: 139.68521; geodeticDatum: WGS84; **Identification:** identifiedBy: T. Ishikawa; dateIdentified: 2013; **Event:** samplingProtocol: light trap; eventDate: 2013-08-02; **Record Level:** institutionCode: KMUT; collectionCode: IC
- **Type status:** Other material. **Occurrence:** recordedBy: T. Ishikawa; individualCount: 22; sex: 6 males, 16 females; lifeStage: adult; otherCatalogNumbers: 2014-00849 | 2014-00850 | 2014-00851 | 2014-00852 | 2014-00853 | 2014-00854 | 2014-00855 | 2014-00856 | 2014-00857 | 2014-00858 | 2014-00859 | 2014-00860 | 2014-00861 | 2014-00862 | 2014-00863 | 2014-00864 | 2014-00865 | 2014-00866 | 2014-00867 | 2014-00868 | 2014-00869 | 2014-00870; **Taxon:** namePublishedIn: 1909; kingdom: Animalia; phylum: Arthropoda; class: Insecta; order: Hemiptera; family: Anthocoridae; genus: Physopleurella; specificEpithet: armata; scientificNameAuthorship: Poppius; **Location:** country: Japan; stateProvince: Tokyo; municipality: Meguro-ku; locality: The University of Tokyo Campus, Komaba.; minimumElevationInMeters: 31; maximumElevationInMeters: 39; decimalLatitude: 35.66006; decimalLongitude: 139.68521; geodeticDatum: WGS84; **Identification:** identifiedBy: T. Ishikawa; dateIdentified: 2013; **Event:** samplingProtocol: light trap; eventDate: 2013-08-13; **Record Level:** institutionCode: KMUT; collectionCode: IC

#### Reduviidae

Latreille, 1807

#### Empicoris minutus

Usinger, 1946

##### Materials

- **Type status:** Other material. **Occurrence:** recordedBy: T. Ishikawa; individualCount: 4; sex: 2 males, 2 females; lifeStage: adult; otherCatalogNumbers: 2014-01022 | 2014-01023 | 2014-01024 | 2014-01025; **Taxon:** namePublishedIn: 1946; kingdom: Animalia; phylum: Arthropoda; class: Insecta; order: Hemiptera; family: Reduviidae; genus: Empicoris; specificEpithet: minutus; scientificNameAuthorship: Usinger; **Location:** country: Japan; stateProvince: Tokyo; municipality: Meguro-ku; locality: The University of Tokyo Campus, Komaba.; minimumElevationInMeters: 31; maximumElevationInMeters: 39; decimalLatitude: 35.66006; decimalLongitude: 139.68521; geodeticDatum: WGS84; **Identification:** identifiedBy: T. Ishikawa; dateIdentified: 2013; **Event:** samplingProtocol: net sweeping; eventDate: 2013-06-16/2013-06-18; **Record Level:** institutionCode: KMUT; collectionCode: IC
- **Type status:** Other material. **Occurrence:** recordedBy: T. Ishikawa; individualCount: 1; sex: 1 female; lifeStage: adult; otherCatalogNumbers: 2014-01026; **Taxon:** namePublishedIn: 1946; kingdom: Animalia; phylum: Arthropoda; class: Insecta; order: Hemiptera; family: Reduviidae; genus: Empicoris; specificEpithet: minutus; scientificNameAuthorship: Usinger; **Location:** country: Japan; stateProvince: Tokyo; municipality: Meguro-ku; locality: The University of Tokyo Campus, Komaba.; minimumElevationInMeters: 31; maximumElevationInMeters: 39; decimalLatitude: 35.66006; decimalLongitude: 139.68521; geodeticDatum: WGS84; **Identification:** identifiedBy: T. Ishikawa; dateIdentified: 2013; **Event:** samplingProtocol: net sweeping; eventDate: 2013-06-23; **Record Level:** institutionCode: KMUT; collectionCode: IC
- **Type status:** Other material. **Occurrence:** recordedBy: T. Ishikawa; individualCount: 2; sex: 1 male, 1 female; lifeStage: adult; otherCatalogNumbers: 2014-01027 | 2014-01028; **Taxon:** namePublishedIn: 1946; kingdom: Animalia; phylum: Arthropoda; class: Insecta; order: Hemiptera; family: Reduviidae; genus: Empicoris; specificEpithet: minutus; scientificNameAuthorship: Usinger; **Location:** country: Japan; stateProvince: Tokyo; municipality: Meguro-ku; locality: The University of Tokyo Campus, Komaba.; minimumElevationInMeters: 31; maximumElevationInMeters: 39; decimalLatitude: 35.66006; decimalLongitude: 139.68521; geodeticDatum: WGS84; **Identification:** identifiedBy: T. Ishikawa; dateIdentified: 2013; **Event:** samplingProtocol: net sweeping; eventDate: 2013-08-15/2013-08-18; **Record Level:** institutionCode: KMUT; collectionCode: IC
- **Type status:** Other material. **Occurrence:** recordedBy: T. Ishikawa; individualCount: 2; sex: 1 male, 1 female; lifeStage: adult; otherCatalogNumbers: 2014-01029 | 2014-01030; **Taxon:** namePublishedIn: 1946; kingdom: Animalia; phylum: Arthropoda; class: Insecta; order: Hemiptera; family: Reduviidae; genus: Empicoris; specificEpithet: minutus; scientificNameAuthorship: Usinger; **Location:** country: Japan; stateProvince: Tokyo; municipality: Meguro-ku; locality: The University of Tokyo Campus, Komaba.; minimumElevationInMeters: 31; maximumElevationInMeters: 39; decimalLatitude: 35.66006; decimalLongitude: 139.68521; geodeticDatum: WGS84; **Identification:** identifiedBy: T. Ishikawa; dateIdentified: 2013; **Event:** samplingProtocol: net sweeping; eventDate: 2013-08-19; **Record Level:** institutionCode: KMUT; collectionCode: IC
- **Type status:** Other material. **Occurrence:** recordedBy: T. Ishikawa; individualCount: 1; sex: 1 male; lifeStage: adult; otherCatalogNumbers: 2014-01031; **Taxon:** namePublishedIn: 1946; kingdom: Animalia; phylum: Arthropoda; class: Insecta; order: Hemiptera; family: Reduviidae; genus: Empicoris; specificEpithet: minutus; scientificNameAuthorship: Usinger; **Location:** country: Japan; stateProvince: Tokyo; municipality: Meguro-ku; locality: The University of Tokyo Campus, Komaba.; minimumElevationInMeters: 31; maximumElevationInMeters: 39; decimalLatitude: 35.66006; decimalLongitude: 139.68521; geodeticDatum: WGS84; **Identification:** identifiedBy: T. Ishikawa; dateIdentified: 2013; **Event:** samplingProtocol: net sweeping; eventDate: 2013-10-30; **Record Level:** institutionCode: KMUT; collectionCode: IC

#### Haematoloecha nigrorufa

(Stål, 1867)

##### Materials

- **Type status:** Other material. **Occurrence:** recordedBy: T. Ishikawa; individualCount: 1; sex: 1 female; lifeStage: adult; otherCatalogNumbers: 2014-01021; **Taxon:** namePublishedIn: 1867; kingdom: Animalia; phylum: Arthropoda; class: Insecta; order: Hemiptera; family: Reduviidae; genus: Haematoloecha; specificEpithet: nigrorufa; scientificNameAuthorship: Stål; **Location:** country: Japan; stateProvince: Tokyo; municipality: Meguro-ku; locality: The University of Tokyo Campus, Komaba.; minimumElevationInMeters: 31; maximumElevationInMeters: 39; decimalLatitude: 35.66006; decimalLongitude: 139.68521; geodeticDatum: WGS84; **Identification:** identifiedBy: T. Ishikawa; dateIdentified: 2013; **Event:** samplingProtocol: net sweeping; eventDate: 2013-10-03; **Record Level:** institutionCode: KMUT; collectionCode: IC

#### Velinus nodipes

(Uhler, 1860)

##### Materials

- **Type status:** Other material. **Occurrence:** recordedBy: T. Ishikawa; individualCount: 1; sex: 1 female; lifeStage: adult; otherCatalogNumbers: 2014-01032; **Taxon:** namePublishedIn: 1860; kingdom: Animalia; phylum: Arthropoda; class: Insecta; order: Hemiptera; family: Reduviidae; genus: Velinus; specificEpithet: nodipes; scientificNameAuthorship: Uhler; **Location:** country: Japan; stateProvince: Tokyo; municipality: Meguro-ku; locality: The University of Tokyo Campus, Komaba.; minimumElevationInMeters: 31; maximumElevationInMeters: 39; decimalLatitude: 35.66006; decimalLongitude: 139.68521; geodeticDatum: WGS84; **Identification:** identifiedBy: T. Ishikawa; dateIdentified: 2013; **Event:** samplingProtocol: net sweeping; eventDate: 2013-05-18; **Record Level:** institutionCode: KMUT; collectionCode: IC
- **Type status:** Other material. **Occurrence:** recordedBy: T. Ishikawa; individualCount: 1; sex: 1 unknown; lifeStage: nymph; otherCatalogNumbers: 2014-01033; **Taxon:** namePublishedIn: 1860; kingdom: Animalia; phylum: Arthropoda; class: Insecta; order: Hemiptera; family: Reduviidae; genus: Velinus; specificEpithet: nodipes; scientificNameAuthorship: Uhler; **Location:** country: Japan; stateProvince: Tokyo; municipality: Meguro-ku; locality: The University of Tokyo Campus, Komaba.; minimumElevationInMeters: 31; maximumElevationInMeters: 39; decimalLatitude: 35.66006; decimalLongitude: 139.68521; geodeticDatum: WGS84; **Identification:** identifiedBy: T. Ishikawa; dateIdentified: 2013; **Event:** samplingProtocol: net sweeping; eventDate: 2013-10-30; **Record Level:** institutionCode: KMUT; collectionCode: IC
- **Type status:** Other material. **Occurrence:** recordedBy: K. Kishimoto-Yamada; individualCount: 1; sex: 1 unknown; lifeStage: nymph; otherCatalogNumbers: 2014-01034; **Taxon:** namePublishedIn: 1860; kingdom: Animalia; phylum: Arthropoda; class: Insecta; order: Hemiptera; family: Reduviidae; genus: Velinus; specificEpithet: nodipes; scientificNameAuthorship: Uhler; **Location:** country: Japan; stateProvince: Tokyo; municipality: Meguro-ku; locality: The University of Tokyo Campus, Komaba.; minimumElevationInMeters: 31; maximumElevationInMeters: 39; decimalLatitude: 35.66006; decimalLongitude: 139.68521; geodeticDatum: WGS84; **Identification:** identifiedBy: T. Ishikawa; dateIdentified: 2014; **Event:** samplingProtocol: net sweeping; eventDate: 2014-05-01; **Record Level:** institutionCode: KMUT; collectionCode: IC

#### Pentatomomorpha

Leston, Pendergrast et Southwood, 1954

#### Lygaeoidea

Schilling, 1829

#### Pachygronthidae

Stål, 1865

#### Pachygrontha antennata

(Uhler, 1860)

##### Materials

- **Type status:** Other material. **Occurrence:** recordedBy: T. Kato; individualCount: 1; sex: 1 female; lifeStage: adult; otherCatalogNumbers: 2014-01035; **Taxon:** namePublishedIn: 1860; kingdom: Animalia; phylum: Arthropoda; class: Insecta; order: Hemiptera; family: Pachygronthidae; genus: Pachygrontha; specificEpithet: antennata; scientificNameAuthorship: Uhler; **Location:** country: Japan; stateProvince: Tokyo; municipality: Meguro-ku; locality: The University of Tokyo Campus, Komaba.; minimumElevationInMeters: 31; maximumElevationInMeters: 39; decimalLatitude: 35.66006; decimalLongitude: 139.68521; geodeticDatum: WGS84; **Identification:** identifiedBy: T. Ishikawa; dateIdentified: 2013; **Event:** samplingProtocol: net sweeping; eventDate: 2013-04-25; **Record Level:** institutionCode: KMUT; collectionCode: IC
- **Type status:** Other material. **Occurrence:** recordedBy: T. Ishikawa; individualCount: 2; sex: 2 females; lifeStage: adult; otherCatalogNumbers: 2014-01036 | 2014-01037; **Taxon:** namePublishedIn: 1860; kingdom: Animalia; phylum: Arthropoda; class: Insecta; order: Hemiptera; family: Pachygronthidae; genus: Pachygrontha; specificEpithet: antennata; scientificNameAuthorship: Uhler; **Location:** country: Japan; stateProvince: Tokyo; municipality: Meguro-ku; locality: The University of Tokyo Campus, Komaba.; minimumElevationInMeters: 31; maximumElevationInMeters: 39; decimalLatitude: 35.66006; decimalLongitude: 139.68521; geodeticDatum: WGS84; **Identification:** identifiedBy: T. Ishikawa; dateIdentified: 2013; **Event:** samplingProtocol: net sweeping; eventDate: 2013-04-28; **Record Level:** institutionCode: KMUT; collectionCode: IC
- **Type status:** Other material. **Occurrence:** recordedBy: T. Ishikawa; individualCount: 1; sex: 1 female; lifeStage: adult; otherCatalogNumbers: 2014-01038; **Taxon:** namePublishedIn: 1860; kingdom: Animalia; phylum: Arthropoda; class: Insecta; order: Hemiptera; family: Pachygronthidae; genus: Pachygrontha; specificEpithet: antennata; scientificNameAuthorship: Uhler; **Location:** country: Japan; stateProvince: Tokyo; municipality: Meguro-ku; locality: The University of Tokyo Campus, Komaba.; minimumElevationInMeters: 31; maximumElevationInMeters: 39; decimalLatitude: 35.66006; decimalLongitude: 139.68521; geodeticDatum: WGS84; **Identification:** identifiedBy: T. Ishikawa; dateIdentified: 2013; **Event:** samplingProtocol: net sweeping; eventDate: 2013-05-12; **Record Level:** institutionCode: KMUT; collectionCode: IC
- **Type status:** Other material. **Occurrence:** recordedBy: T. Ishikawa; individualCount: 2; sex: 2 females; lifeStage: adult; otherCatalogNumbers: 2014-01039 | 2014-01040; **Taxon:** namePublishedIn: 1860; kingdom: Animalia; phylum: Arthropoda; class: Insecta; order: Hemiptera; family: Pachygronthidae; genus: Pachygrontha; specificEpithet: antennata; scientificNameAuthorship: Uhler; **Location:** country: Japan; stateProvince: Tokyo; municipality: Meguro-ku; locality: The University of Tokyo Campus, Komaba.; minimumElevationInMeters: 31; maximumElevationInMeters: 39; decimalLatitude: 35.66006; decimalLongitude: 139.68521; geodeticDatum: WGS84; **Identification:** identifiedBy: T. Ishikawa; dateIdentified: 2013; **Event:** samplingProtocol: net sweeping; eventDate: 2013-05-19; **Record Level:** institutionCode: KMUT; collectionCode: IC
- **Type status:** Other material. **Occurrence:** recordedBy: T. Ishikawa; individualCount: 3; sex: 1 male, 2 females; lifeStage: adult; otherCatalogNumbers: 2014-01041 | 2014-01042 | 2014-01043; **Taxon:** namePublishedIn: 1860; kingdom: Animalia; phylum: Arthropoda; class: Insecta; order: Hemiptera; family: Pachygronthidae; genus: Pachygrontha; specificEpithet: antennata; scientificNameAuthorship: Uhler; **Location:** country: Japan; stateProvince: Tokyo; municipality: Meguro-ku; locality: The University of Tokyo Campus, Komaba.; minimumElevationInMeters: 31; maximumElevationInMeters: 39; decimalLatitude: 35.66006; decimalLongitude: 139.68521; geodeticDatum: WGS84; **Identification:** identifiedBy: T. Ishikawa; dateIdentified: 2013; **Event:** samplingProtocol: net sweeping; eventDate: 2013-05-25; **Record Level:** institutionCode: KMUT; collectionCode: IC
- **Type status:** Other material. **Occurrence:** recordedBy: T. Ishikawa; individualCount: 4; sex: 2 males, 2 females; lifeStage: adult; otherCatalogNumbers: 2014-01044 | 2014-01045 | 2014-01046 | 2014-01047; **Taxon:** namePublishedIn: 1860; kingdom: Animalia; phylum: Arthropoda; class: Insecta; order: Hemiptera; family: Pachygronthidae; genus: Pachygrontha; specificEpithet: antennata; scientificNameAuthorship: Uhler; **Location:** country: Japan; stateProvince: Tokyo; municipality: Meguro-ku; locality: The University of Tokyo Campus, Komaba.; minimumElevationInMeters: 31; maximumElevationInMeters: 39; decimalLatitude: 35.66006; decimalLongitude: 139.68521; geodeticDatum: WGS84; **Identification:** identifiedBy: T. Ishikawa; dateIdentified: 2013; **Event:** samplingProtocol: net sweeping; eventDate: 2013-06-22/2013-06-23; **Record Level:** institutionCode: KMUT; collectionCode: IC
- **Type status:** Other material. **Occurrence:** recordedBy: T. Ishikawa; individualCount: 14; sex: 9 males, 5 females; lifeStage: adult; otherCatalogNumbers: 2014-01048 | 2014-01049 | 2014-01050 | 2014-01051 | 2014-01052 | 2014-01053 | 2014-01054 | 2014-01055 | 2014-01056 | 2014-01057 | 2014-01058 | 2014-01059 | 2014-01060 | 2014-01061; **Taxon:** namePublishedIn: 1860; kingdom: Animalia; phylum: Arthropoda; class: Insecta; order: Hemiptera; family: Pachygronthidae; genus: Pachygrontha; specificEpithet: antennata; scientificNameAuthorship: Uhler; **Location:** country: Japan; stateProvince: Tokyo; municipality: Meguro-ku; locality: The University of Tokyo Campus, Komaba.; minimumElevationInMeters: 31; maximumElevationInMeters: 39; decimalLatitude: 35.66006; decimalLongitude: 139.68521; geodeticDatum: WGS84; **Identification:** identifiedBy: T. Ishikawa; dateIdentified: 2013; **Event:** samplingProtocol: net sweeping; eventDate: 2013-08-15; **Record Level:** institutionCode: KMUT; collectionCode: IC

#### Pachygrontha similis

Uhler, 1896

##### Materials

- **Type status:** Other material. **Occurrence:** recordedBy: T. Ishikawa; individualCount: 2; sex: 2 males; lifeStage: adult; otherCatalogNumbers: 2014-01062 | 2014-01063; **Taxon:** namePublishedIn: 1896; kingdom: Animalia; phylum: Arthropoda; class: Insecta; order: Hemiptera; family: Pachygronthidae; genus: Pachygrontha; specificEpithet: similis; scientificNameAuthorship: Uhler; **Location:** country: Japan; stateProvince: Tokyo; municipality: Meguro-ku; locality: The University of Tokyo Campus, Komaba.; minimumElevationInMeters: 31; maximumElevationInMeters: 39; decimalLatitude: 35.66006; decimalLongitude: 139.68521; geodeticDatum: WGS84; **Identification:** identifiedBy: T. Ishikawa; dateIdentified: 2013; **Event:** samplingProtocol: net sweeping; eventDate: 2013-08-18; **Record Level:** institutionCode: KMUT; collectionCode: IC

#### Rhyparochromidae

Amyot et Serville, 1843

#### Botocudo japonicus

(Hidaka, 1959)

##### Materials

- **Type status:** Other material. **Occurrence:** recordedBy: T. Ishikawa; individualCount: 1; sex: 1 female; lifeStage: adult; otherCatalogNumbers: 2014-01064; **Taxon:** namePublishedIn: 1959; kingdom: Animalia; phylum: Arthropoda; class: Insecta; order: Hemiptera; family: Rhyparochromidae; genus: Botocudo; specificEpithet: japonicus; scientificNameAuthorship: Hidaka; **Location:** country: Japan; stateProvince: Tokyo; municipality: Meguro-ku; locality: The University of Tokyo Campus, Komaba.; minimumElevationInMeters: 31; maximumElevationInMeters: 39; decimalLatitude: 35.66006; decimalLongitude: 139.68521; geodeticDatum: WGS84; **Identification:** identifiedBy: T. Ishikawa; dateIdentified: 2013; **Event:** samplingProtocol: light trap; eventDate: 2013-08-02; **Record Level:** institutionCode: KMUT; collectionCode: IC

#### Gyndes pallicornis

(Dallas, 1852)

##### Materials

- **Type status:** Other material. **Occurrence:** recordedBy: T. Ishikawa; individualCount: 1; sex: 1 male; lifeStage: adult; otherCatalogNumbers: 2014-01066; **Taxon:** namePublishedIn: 1852; kingdom: Animalia; phylum: Arthropoda; class: Insecta; order: Hemiptera; family: Rhyparochromidae; genus: Gyndes; specificEpithet: pallicornis; scientificNameAuthorship: Dallas; **Location:** country: Japan; stateProvince: Tokyo; municipality: Meguro-ku; locality: The University of Tokyo Campus, Komaba.; minimumElevationInMeters: 31; maximumElevationInMeters: 39; decimalLatitude: 35.66006; decimalLongitude: 139.68521; geodeticDatum: WGS84; **Identification:** identifiedBy: T. Ishikawa; dateIdentified: 2013; **Event:** samplingProtocol: light trap; eventDate: 2013-05-14; **Record Level:** institutionCode: KMUT; collectionCode: IC
- **Type status:** Other material. **Occurrence:** recordedBy: T. Ishikawa; individualCount: 2; sex: 2 males; lifeStage: adult; otherCatalogNumbers: 2014-01067 | 2014-01068; **Taxon:** namePublishedIn: 1852; kingdom: Animalia; phylum: Arthropoda; class: Insecta; order: Hemiptera; family: Rhyparochromidae; genus: Gyndes; specificEpithet: pallicornis; scientificNameAuthorship: Dallas; **Location:** country: Japan; stateProvince: Tokyo; municipality: Meguro-ku; locality: The University of Tokyo Campus, Komaba.; minimumElevationInMeters: 31; maximumElevationInMeters: 39; decimalLatitude: 35.66006; decimalLongitude: 139.68521; geodeticDatum: WGS84; **Identification:** identifiedBy: T. Ishikawa; dateIdentified: 2013; **Event:** samplingProtocol: net sweeping; eventDate: 2013-05-19; **Record Level:** institutionCode: KMUT; collectionCode: IC
- **Type status:** Other material. **Occurrence:** recordedBy: T. Ishikawa; individualCount: 1; sex: 1 female; lifeStage: adult; otherCatalogNumbers: 2014-01069; **Taxon:** namePublishedIn: 1852; kingdom: Animalia; phylum: Arthropoda; class: Insecta; order: Hemiptera; family: Rhyparochromidae; genus: Gyndes; specificEpithet: pallicornis; scientificNameAuthorship: Dallas; **Location:** country: Japan; stateProvince: Tokyo; municipality: Meguro-ku; locality: The University of Tokyo Campus, Komaba.; minimumElevationInMeters: 31; maximumElevationInMeters: 39; decimalLatitude: 35.66006; decimalLongitude: 139.68521; geodeticDatum: WGS84; **Identification:** identifiedBy: T. Ishikawa; dateIdentified: 2013; **Event:** samplingProtocol: net sweeping; eventDate: 2013-06-23; **Record Level:** institutionCode: KMUT; collectionCode: IC
- **Type status:** Other material. **Occurrence:** recordedBy: T. Ishikawa; individualCount: 3; sex: 3 females; lifeStage: adult; otherCatalogNumbers: 2014-01070 | 2014-01071 | 2014-01072; **Taxon:** namePublishedIn: 1852; kingdom: Animalia; phylum: Arthropoda; class: Insecta; order: Hemiptera; family: Rhyparochromidae; genus: Gyndes; specificEpithet: pallicornis; scientificNameAuthorship: Dallas; **Location:** country: Japan; stateProvince: Tokyo; municipality: Meguro-ku; locality: The University of Tokyo Campus, Komaba.; minimumElevationInMeters: 31; maximumElevationInMeters: 39; decimalLatitude: 35.66006; decimalLongitude: 139.68521; geodeticDatum: WGS84; **Identification:** identifiedBy: T. Ishikawa; dateIdentified: 2013; **Event:** samplingProtocol: net sweeping; eventDate: 2013-08-15; **Record Level:** institutionCode: KMUT; collectionCode: IC
- **Type status:** Other material. **Occurrence:** recordedBy: T. Ishikawa; individualCount: 3; sex: 3 females; lifeStage: adult; otherCatalogNumbers: 2014-01073 | 2014-01074 | 2014-01075; **Taxon:** namePublishedIn: 1852; kingdom: Animalia; phylum: Arthropoda; class: Insecta; order: Hemiptera; family: Rhyparochromidae; genus: Gyndes; specificEpithet: pallicornis; scientificNameAuthorship: Dallas; **Location:** country: Japan; stateProvince: Tokyo; municipality: Meguro-ku; locality: The University of Tokyo Campus, Komaba.; minimumElevationInMeters: 31; maximumElevationInMeters: 39; decimalLatitude: 35.66006; decimalLongitude: 139.68521; geodeticDatum: WGS84; **Identification:** identifiedBy: T. Ishikawa; dateIdentified: 2013; **Event:** samplingProtocol: net sweeping; eventDate: 2013-10-30; **Record Level:** institutionCode: KMUT; collectionCode: IC

#### Metochus abbreviatus

Scott, 1874

##### Materials

- **Type status:** Other material. **Occurrence:** recordedBy: T. Ishikawa; individualCount: 2; sex: 1 male, 1 female; lifeStage: adult; otherCatalogNumbers: 2014-01096 | 2014-01097; **Taxon:** namePublishedIn: 1874; kingdom: Animalia; phylum: Arthropoda; class: Insecta; order: Hemiptera; family: Rhyparochromidae; genus: Metochus; specificEpithet: abbreviatus; scientificNameAuthorship: Scott; **Location:** country: Japan; stateProvince: Tokyo; municipality: Meguro-ku; locality: The University of Tokyo Campus, Komaba.; minimumElevationInMeters: 31; maximumElevationInMeters: 39; decimalLatitude: 35.66006; decimalLongitude: 139.68521; geodeticDatum: WGS84; **Identification:** identifiedBy: T. Ishikawa; dateIdentified: 2013; **Event:** samplingProtocol: light trap; eventDate: 2013-08-13; **Record Level:** institutionCode: KMUT; collectionCode: IC
- **Type status:** Other material. **Occurrence:** recordedBy: T. Ishikawa; individualCount: 1; sex: 1 female; lifeStage: adult; otherCatalogNumbers: 2014-01098; **Taxon:** namePublishedIn: 1874; kingdom: Animalia; phylum: Arthropoda; class: Insecta; order: Hemiptera; family: Rhyparochromidae; genus: Metochus; specificEpithet: abbreviatus; scientificNameAuthorship: Scott; **Location:** country: Japan; stateProvince: Tokyo; municipality: Meguro-ku; locality: The University of Tokyo Campus, Komaba.; minimumElevationInMeters: 31; maximumElevationInMeters: 39; decimalLatitude: 35.66006; decimalLongitude: 139.68521; geodeticDatum: WGS84; **Identification:** identifiedBy: T. Ishikawa; dateIdentified: 2013; **Event:** samplingProtocol: net sweeping; eventDate: 2013-09-01; **Record Level:** institutionCode: KMUT; collectionCode: IC

#### Neolethaeus dallasi

(Scott, 1874)

##### Materials

- **Type status:** Other material. **Occurrence:** recordedBy: T. Ishikawa; individualCount: 1; sex: 1 female; lifeStage: adult; otherCatalogNumbers: 2014-01065; **Taxon:** namePublishedIn: 1874; kingdom: Animalia; phylum: Arthropoda; class: Insecta; order: Hemiptera; family: Rhyparochromidae; genus: Neolethaeus; specificEpithet: dallasi; scientificNameAuthorship: Scott; **Location:** country: Japan; stateProvince: Tokyo; municipality: Meguro-ku; locality: The University of Tokyo Campus, Komaba.; minimumElevationInMeters: 31; maximumElevationInMeters: 39; decimalLatitude: 35.66006; decimalLongitude: 139.68521; geodeticDatum: WGS84; **Identification:** identifiedBy: T. Ishikawa; dateIdentified: 2013; **Event:** samplingProtocol: net sweeping; eventDate: 2013-10-31; **Record Level:** institutionCode: KMUT; collectionCode: IC

#### Pamerana scotti

(Distant, 1901)

##### Materials

- **Type status:** Other material. **Occurrence:** recordedBy: T. Ishikawa; individualCount: 1; sex: 1 female; lifeStage: adult; otherCatalogNumbers: 2014-01076; **Taxon:** namePublishedIn: 1901; kingdom: Animalia; phylum: Arthropoda; class: Insecta; order: Hemiptera; family: Rhyparochromidae; genus: Pamerana; specificEpithet: scotti; scientificNameAuthorship: Distant; **Location:** country: Japan; stateProvince: Tokyo; municipality: Meguro-ku; locality: The University of Tokyo Campus, Komaba.; minimumElevationInMeters: 31; maximumElevationInMeters: 39; decimalLatitude: 35.66006; decimalLongitude: 139.68521; geodeticDatum: WGS84; **Identification:** identifiedBy: T. Ishikawa; dateIdentified: 2013; **Event:** samplingProtocol: light trap; eventDate: 2013-05-10; **Record Level:** institutionCode: KMUT; collectionCode: IC
- **Type status:** Other material. **Occurrence:** recordedBy: T. Ishikawa; individualCount: 1; sex: 1 female; lifeStage: adult; otherCatalogNumbers: 2014-01077; **Taxon:** namePublishedIn: 1901; kingdom: Animalia; phylum: Arthropoda; class: Insecta; order: Hemiptera; family: Rhyparochromidae; genus: Pamerana; specificEpithet: scotti; scientificNameAuthorship: Distant; **Location:** country: Japan; stateProvince: Tokyo; municipality: Meguro-ku; locality: The University of Tokyo Campus, Komaba.; minimumElevationInMeters: 31; maximumElevationInMeters: 39; decimalLatitude: 35.66006; decimalLongitude: 139.68521; geodeticDatum: WGS84; **Identification:** identifiedBy: T. Ishikawa; dateIdentified: 2013; **Event:** samplingProtocol: net sweeping; eventDate: 2013-05-12; **Record Level:** institutionCode: KMUT; collectionCode: IC
- **Type status:** Other material. **Occurrence:** recordedBy: T. Ishikawa; individualCount: 1; sex: 1 female; lifeStage: adult; otherCatalogNumbers: 2014-01078; **Taxon:** namePublishedIn: 1901; kingdom: Animalia; phylum: Arthropoda; class: Insecta; order: Hemiptera; family: Rhyparochromidae; genus: Pamerana; specificEpithet: scotti; scientificNameAuthorship: Distant; **Location:** country: Japan; stateProvince: Tokyo; municipality: Meguro-ku; locality: The University of Tokyo Campus, Komaba.; minimumElevationInMeters: 31; maximumElevationInMeters: 39; decimalLatitude: 35.66006; decimalLongitude: 139.68521; geodeticDatum: WGS84; **Identification:** identifiedBy: T. Ishikawa; dateIdentified: 2013; **Event:** samplingProtocol: net sweeping; eventDate: 2013-08-15; **Record Level:** institutionCode: KMUT; collectionCode: IC

#### Panaorus japonicus

(Stål, 1874)

##### Materials

- **Type status:** Other material. **Occurrence:** recordedBy: T. Ishikawa; individualCount: 1; sex: 1 female; lifeStage: adult; otherCatalogNumbers: 2014-01099; **Taxon:** namePublishedIn: 1874; kingdom: Animalia; phylum: Arthropoda; class: Insecta; order: Hemiptera; family: Rhyparochromidae; genus: Panaorus; specificEpithet: japonicus; scientificNameAuthorship: Stål; **Location:** country: Japan; stateProvince: Tokyo; municipality: Meguro-ku; locality: The University of Tokyo Campus, Komaba.; minimumElevationInMeters: 31; maximumElevationInMeters: 39; decimalLatitude: 35.66006; decimalLongitude: 139.68521; geodeticDatum: WGS84; **Identification:** identifiedBy: T. Ishikawa; dateIdentified: 2013; **Event:** samplingProtocol: net sweeping; eventDate: 2013-10-21; **Record Level:** institutionCode: KMUT; collectionCode: IC

#### Stigmatonotum geniculatum

(Motschulsky, 1863)

##### Materials

- **Type status:** Other material. **Occurrence:** recordedBy: T. Ishikawa; individualCount: 1; sex: 1 female; lifeStage: adult; otherCatalogNumbers: 2014-01079; **Taxon:** namePublishedIn: 1863; kingdom: Animalia; phylum: Arthropoda; class: Insecta; order: Hemiptera; family: Rhyparochromidae; genus: Stigmatonotum; specificEpithet: geniculatum; scientificNameAuthorship: Motschulsky; **Location:** country: Japan; stateProvince: Tokyo; municipality: Meguro-ku; locality: The University of Tokyo Campus, Komaba.; minimumElevationInMeters: 31; maximumElevationInMeters: 39; decimalLatitude: 35.66006; decimalLongitude: 139.68521; geodeticDatum: WGS84; **Identification:** identifiedBy: T. Ishikawa; dateIdentified: 2013; **Event:** samplingProtocol: light trap; eventDate: 2013-08-02; **Record Level:** institutionCode: KMUT; collectionCode: IC

#### Togo hemipterus

(Scott, 1874)

##### Materials

- **Type status:** Other material. **Occurrence:** recordedBy: T. Ishikawa; individualCount: 9; sex: 9 females; lifeStage: adult; otherCatalogNumbers: 2014-01080 | 2014-01081 | 2014-01082 | 2014-01083 | 2014-01084 | 2014-01085 | 2014-01086 | 2014-01087 | 2014-01088; **Taxon:** namePublishedIn: 1874; kingdom: Animalia; phylum: Arthropoda; class: Insecta; order: Hemiptera; family: Rhyparochromidae; genus: Togo; specificEpithet: hemipterus; scientificNameAuthorship: Scott; **Location:** country: Japan; stateProvince: Tokyo; municipality: Meguro-ku; locality: The University of Tokyo Campus, Komaba.; minimumElevationInMeters: 31; maximumElevationInMeters: 39; decimalLatitude: 35.66006; decimalLongitude: 139.68521; geodeticDatum: WGS84; **Identification:** identifiedBy: T. Ishikawa; dateIdentified: 2013; **Event:** samplingProtocol: net sweeping; eventDate: 2013-05-12; **Record Level:** institutionCode: KMUT; collectionCode: IC
- **Type status:** Other material. **Occurrence:** recordedBy: T. Ishikawa; individualCount: 4; sex: 1 male, 3 females; lifeStage: adult; otherCatalogNumbers: 2014-01089 | 2014-01090 | 2014-01091 | 2014-01092; **Taxon:** namePublishedIn: 1874; kingdom: Animalia; phylum: Arthropoda; class: Insecta; order: Hemiptera; family: Rhyparochromidae; genus: Togo; specificEpithet: hemipterus; scientificNameAuthorship: Scott; **Location:** country: Japan; stateProvince: Tokyo; municipality: Meguro-ku; locality: The University of Tokyo Campus, Komaba.; minimumElevationInMeters: 31; maximumElevationInMeters: 39; decimalLatitude: 35.66006; decimalLongitude: 139.68521; geodeticDatum: WGS84; **Identification:** identifiedBy: T. Ishikawa; dateIdentified: 2013; **Event:** samplingProtocol: net sweeping; eventDate: 2013-05-19; **Record Level:** institutionCode: KMUT; collectionCode: IC
- **Type status:** Other material. **Occurrence:** recordedBy: T. Ishikawa; individualCount: 1; sex: 1 female; lifeStage: adult; otherCatalogNumbers: 2014-01093; **Taxon:** namePublishedIn: 1874; kingdom: Animalia; phylum: Arthropoda; class: Insecta; order: Hemiptera; family: Rhyparochromidae; genus: Togo; specificEpithet: hemipterus; scientificNameAuthorship: Scott; **Location:** country: Japan; stateProvince: Tokyo; municipality: Meguro-ku; locality: The University of Tokyo Campus, Komaba.; minimumElevationInMeters: 31; maximumElevationInMeters: 39; decimalLatitude: 35.66006; decimalLongitude: 139.68521; geodeticDatum: WGS84; **Identification:** identifiedBy: T. Ishikawa; dateIdentified: 2013; **Event:** samplingProtocol: net sweeping; eventDate: 2013-06-22; **Record Level:** institutionCode: KMUT; collectionCode: IC
- **Type status:** Other material. **Occurrence:** recordedBy: T. Ishikawa; individualCount: 1; sex: 1 female; lifeStage: adult; otherCatalogNumbers: 2014-01094; **Taxon:** namePublishedIn: 1874; kingdom: Animalia; phylum: Arthropoda; class: Insecta; order: Hemiptera; family: Rhyparochromidae; genus: Togo; specificEpithet: hemipterus; scientificNameAuthorship: Scott; **Location:** country: Japan; stateProvince: Tokyo; municipality: Meguro-ku; locality: The University of Tokyo Campus, Komaba.; minimumElevationInMeters: 31; maximumElevationInMeters: 39; decimalLatitude: 35.66006; decimalLongitude: 139.68521; geodeticDatum: WGS84; **Identification:** identifiedBy: T. Ishikawa; dateIdentified: 2013; **Event:** samplingProtocol: net sweeping; eventDate: 2013-08-15; **Record Level:** institutionCode: KMUT; collectionCode: IC
- **Type status:** Other material. **Occurrence:** recordedBy: T. Ishikawa; individualCount: 1; sex: 1 male; lifeStage: adult; otherCatalogNumbers: 2014-01095; **Taxon:** namePublishedIn: 1874; kingdom: Animalia; phylum: Arthropoda; class: Insecta; order: Hemiptera; family: Rhyparochromidae; genus: Togo; specificEpithet: hemipterus; scientificNameAuthorship: Scott; **Location:** country: Japan; stateProvince: Tokyo; municipality: Meguro-ku; locality: The University of Tokyo Campus, Komaba.; minimumElevationInMeters: 31; maximumElevationInMeters: 39; decimalLatitude: 35.66006; decimalLongitude: 139.68521; geodeticDatum: WGS84; **Identification:** identifiedBy: T. Ishikawa; dateIdentified: 2013; **Event:** samplingProtocol: net sweeping; eventDate: 2013-10-30; **Record Level:** institutionCode: KMUT; collectionCode: IC

#### Geocoridae

Dahlbom, 1851

#### Geocoris proteus

Distant, 1883

##### Materials

- **Type status:** Other material. **Occurrence:** recordedBy: T. Ishikawa; individualCount: 20; sex: 2 males, 18 females; lifeStage: adult; otherCatalogNumbers: 2014-01100 | 2014-01101 | 2014-01102 | 2014-01103 | 2014-01104 | 2014-01105 | 2014-01106 | 2014-01107 | 2014-01108 | 2014-01109 | 2014-01110 | 2014-01111 | 2014-01112 | 2014-01113 | 2014-01114 | 2014-01115 | 2014-01116 | 2014-01117 | 2014-01118 | 2014-01119; **Taxon:** namePublishedIn: 1883; kingdom: Animalia; phylum: Arthropoda; class: Insecta; order: Hemiptera; family: Geocoridae; genus: Geocoris; specificEpithet: proteus; scientificNameAuthorship: Distant; **Location:** country: Japan; stateProvince: Tokyo; municipality: Meguro-ku; locality: The University of Tokyo Campus, Komaba.; minimumElevationInMeters: 31; maximumElevationInMeters: 39; decimalLatitude: 35.66006; decimalLongitude: 139.68521; geodeticDatum: WGS84; **Identification:** identifiedBy: T. Ishikawa; dateIdentified: 2013; **Event:** samplingProtocol: net sweeping; eventDate: 2013-09-06; **Record Level:** institutionCode: KMUT; collectionCode: IC

#### Geocoris varius

(Uhler, 1860)

##### Materials

- **Type status:** Other material. **Occurrence:** recordedBy: T. Ishikawa; individualCount: 1; sex: 1 female; lifeStage: adult; otherCatalogNumbers: 2014-01120; **Taxon:** namePublishedIn: 1860; kingdom: Animalia; phylum: Arthropoda; class: Insecta; order: Hemiptera; family: Geocoridae; genus: Geocoris; specificEpithet: varius; scientificNameAuthorship: Uhler; **Location:** country: Japan; stateProvince: Tokyo; municipality: Meguro-ku; locality: The University of Tokyo Campus, Komaba.; minimumElevationInMeters: 31; maximumElevationInMeters: 39; decimalLatitude: 35.66006; decimalLongitude: 139.68521; geodeticDatum: WGS84; **Identification:** identifiedBy: T. Ishikawa; dateIdentified: 2013; **Event:** samplingProtocol: net sweeping; eventDate: 2013-08-15; **Record Level:** institutionCode: KMUT; collectionCode: IC

#### Blissidae

Stål, 1862

#### Dimorphopterus bicoloripes

(Distant, 1883)

##### Materials

- **Type status:** Other material. **Occurrence:** recordedBy: T. Ishikawa; individualCount: 8; sex: 5 males, 3 females; lifeStage: adult; otherCatalogNumbers: 2014-01121 | 2014-01122 | 2014-01123 | 2014-01124 | 2014-01125 | 2014-01126 | 2014-01127 | 2014-01128; **Taxon:** namePublishedIn: 1883; kingdom: Animalia; phylum: Arthropoda; class: Insecta; order: Hemiptera; family: Blissidae; genus: Dimorphopterus; specificEpithet: bicoloripes; scientificNameAuthorship: Distant; **Location:** country: Japan; stateProvince: Tokyo; municipality: Meguro-ku; locality: The University of Tokyo Campus, Komaba.; minimumElevationInMeters: 31; maximumElevationInMeters: 39; decimalLatitude: 35.66006; decimalLongitude: 139.68521; geodeticDatum: WGS84; **Identification:** identifiedBy: T. Ishikawa; dateIdentified: 2013; **Event:** samplingProtocol: net sweeping; eventDate: 2013-05-18; **Record Level:** institutionCode: KMUT; collectionCode: IC
- **Type status:** Other material. **Occurrence:** recordedBy: T. Ishikawa; individualCount: 37; sex: 21 males, 16 females; lifeStage: adult; otherCatalogNumbers: 2014-01129 | 2014-01130 | 2014-01131 | 2014-01132 | 2014-01133 | 2014-01134 | 2014-01135 | 2014-01136 | 2014-01137 | 2014-01138 | 2014-01139 | 2014-01140 | 2014-01141 | 2014-01142 | 2014-01143 | 2014-01144 | 2014-01145 | 2014-01146 | 2014-01147 | 2014-01148 | 2014-01149 | 2014-01150 | 2014-01151 | 2014-01152 | 2014-01153 | 2014-01154 | 2014-01155 | 2014-01156 | 2014-01157 | 2014-01158 | 2014-01159 | 2014-01160 | 2014-01161 | 2014-01162 | 2014-01163 | 2014-01164 | 2014-01165; **Taxon:** namePublishedIn: 1883; kingdom: Animalia; phylum: Arthropoda; class: Insecta; order: Hemiptera; family: Blissidae; genus: Dimorphopterus; specificEpithet: bicoloripes; scientificNameAuthorship: Distant; **Location:** country: Japan; stateProvince: Tokyo; municipality: Meguro-ku; locality: The University of Tokyo Campus, Komaba.; minimumElevationInMeters: 31; maximumElevationInMeters: 39; decimalLatitude: 35.66006; decimalLongitude: 139.68521; geodeticDatum: WGS84; **Identification:** identifiedBy: T. Ishikawa; dateIdentified: 2014; **Event:** samplingProtocol: net sweeping; eventDate: 2014-01-01; **Record Level:** institutionCode: KMUT; collectionCode: IC

#### Lygaeidae

Schilling, 1829

#### Nysius plebeius

Distant, 1883

##### Materials

- **Type status:** Other material. **Occurrence:** recordedBy: T. Ishikawa; individualCount: 2; sex: 2 males; lifeStage: adult; otherCatalogNumbers: 2014-01166 | 2014-01167; **Taxon:** namePublishedIn: 1883; kingdom: Animalia; phylum: Arthropoda; class: Insecta; order: Hemiptera; family: Lygaeidae; genus: Nysius; specificEpithet: plebeius; scientificNameAuthorship: Distant; **Location:** country: Japan; stateProvince: Tokyo; municipality: Meguro-ku; locality: The University of Tokyo Campus, Komaba.; minimumElevationInMeters: 31; maximumElevationInMeters: 39; decimalLatitude: 35.66006; decimalLongitude: 139.68521; geodeticDatum: WGS84; **Identification:** identifiedBy: T. Ishikawa; dateIdentified: 2013; **Event:** samplingProtocol: net sweeping; eventDate: 2013-06-23; **Record Level:** institutionCode: KMUT; collectionCode: IC
- **Type status:** Other material. **Occurrence:** recordedBy: T. Ishikawa; individualCount: 8; sex: 2 males, 6 females; lifeStage: adult; otherCatalogNumbers: 2014-01168 | 2014-01169 | 2014-01170 | 2014-01171 | 2014-01172 | 2014-01173 | 2014-01174 | 2014-01175; **Taxon:** namePublishedIn: 1883; kingdom: Animalia; phylum: Arthropoda; class: Insecta; order: Hemiptera; family: Lygaeidae; genus: Nysius; specificEpithet: plebeius; scientificNameAuthorship: Distant; **Location:** country: Japan; stateProvince: Tokyo; municipality: Meguro-ku; locality: The University of Tokyo Campus, Komaba.; minimumElevationInMeters: 31; maximumElevationInMeters: 39; decimalLatitude: 35.66006; decimalLongitude: 139.68521; geodeticDatum: WGS84; **Identification:** identifiedBy: T. Ishikawa; dateIdentified: 2013; **Event:** samplingProtocol: net sweeping; eventDate: 2013-08-15; **Record Level:** institutionCode: KMUT; collectionCode: IC
- **Type status:** Other material. **Occurrence:** recordedBy: T. Ishikawa; individualCount: 3; sex: 3 males; lifeStage: adult; otherCatalogNumbers: 2014-01176 | 2014-01177 | 2014-01178; **Taxon:** namePublishedIn: 1883; kingdom: Animalia; phylum: Arthropoda; class: Insecta; order: Hemiptera; family: Lygaeidae; genus: Nysius; specificEpithet: plebeius; scientificNameAuthorship: Distant; **Location:** country: Japan; stateProvince: Tokyo; municipality: Meguro-ku; locality: The University of Tokyo Campus, Komaba.; minimumElevationInMeters: 31; maximumElevationInMeters: 39; decimalLatitude: 35.66006; decimalLongitude: 139.68521; geodeticDatum: WGS84; **Identification:** identifiedBy: T. Ishikawa; dateIdentified: 2013; **Event:** samplingProtocol: net sweeping; eventDate: 2013-09-06; **Record Level:** institutionCode: KMUT; collectionCode: IC
- **Type status:** Other material. **Occurrence:** recordedBy: T. Ishikawa; individualCount: 2; sex: 1 male, 1 female; lifeStage: adult; otherCatalogNumbers: 2014-01179 | 2014-01180; **Taxon:** namePublishedIn: 1883; kingdom: Animalia; phylum: Arthropoda; class: Insecta; order: Hemiptera; family: Lygaeidae; genus: Nysius; specificEpithet: plebeius; scientificNameAuthorship: Distant; **Location:** country: Japan; stateProvince: Tokyo; municipality: Meguro-ku; locality: The University of Tokyo Campus, Komaba.; minimumElevationInMeters: 31; maximumElevationInMeters: 39; decimalLatitude: 35.66006; decimalLongitude: 139.68521; geodeticDatum: WGS84; **Identification:** identifiedBy: T. Ishikawa; dateIdentified: 2013; **Event:** samplingProtocol: net sweeping; eventDate: 2013-10-30; **Record Level:** institutionCode: KMUT; collectionCode: IC

#### Nysius sp.

##### Materials

- **Type status:** Other material. **Occurrence:** recordedBy: T. Ishikawa; individualCount: 3; sex: 3 females; lifeStage: adult; otherCatalogNumbers: 2014-01181 | 2014-01182 | 2014-01183; **Taxon:** kingdom: Animalia; phylum: Arthropoda; class: Insecta; order: Hemiptera; family: Lygaeidae; genus: Nysius; specificEpithet: sp.; **Location:** country: Japan; stateProvince: Tokyo; municipality: Meguro-ku; locality: The University of Tokyo Campus, Komaba.; minimumElevationInMeters: 31; maximumElevationInMeters: 39; decimalLatitude: 35.66006; decimalLongitude: 139.68521; geodeticDatum: WGS84; **Identification:** identifiedBy: T. Ishikawa; dateIdentified: 2013; **Event:** samplingProtocol: net sweeping; eventDate: 2013-04-28; **Record Level:** institutionCode: KMUT; collectionCode: IC
- **Type status:** Other material. **Occurrence:** recordedBy: T. Ishikawa; individualCount: 4; sex: 3 males, 1 female; lifeStage: adult; otherCatalogNumbers: 2014-01184 | 2014-01185 | 2014-01186 | 2014-01187; **Taxon:** kingdom: Animalia; phylum: Arthropoda; class: Insecta; order: Hemiptera; family: Lygaeidae; genus: Nysius; specificEpithet: sp.; **Location:** country: Japan; stateProvince: Tokyo; municipality: Meguro-ku; locality: The University of Tokyo Campus, Komaba.; minimumElevationInMeters: 31; maximumElevationInMeters: 39; decimalLatitude: 35.66006; decimalLongitude: 139.68521; geodeticDatum: WGS84; **Identification:** identifiedBy: T. Ishikawa; dateIdentified: 2013; **Event:** samplingProtocol: net sweeping; eventDate: 2013-05-19; **Record Level:** institutionCode: KMUT; collectionCode: IC
- **Type status:** Other material. **Occurrence:** recordedBy: T. Ishikawa; individualCount: 104; sex: 42 males, 62 females; lifeStage: adult; otherCatalogNumbers: 2014-01188 | 2014-01189 | 2014-01190 | 2014-01191 | 2014-01192 | 2014-01193 | 2014-01194 | 2014-01195 | 2014-01196 | 2014-01197 | 2014-01198 | 2014-01199 | 2014-01200 | 2014-01201 | 2014-01202 | 2014-01203 | 2014-01204 | 2014-01205 | 2014-01206 | 2014-01207 | 2014-01208 | 2014-01209 | 2014-01210 | 2014-01211 | 2014-01212 | 2014-01213 | 2014-01214 | 2014-01215 | 2014-01216 | 2014-01217 | 2014-01218 | 2014-01219 | 2014-01220 | 2014-01221 | 2014-01222 | 2014-01223 | 2014-01224 | 2014-01225 | 2014-01226 | 2014-01227 | 2014-01228 | 2014-01229 | 2014-01230 | 2014-01231 | 2014-01232 | 2014-01233 | 2014-01234 | 2014-01235 | 2014-01236 | 2014-01237 | 2014-01238 | 2014-01239 | 2014-01240 | 2014-01241 | 2014-01242 | 2014-01243 | 2014-01244 | 2014-01245 | 2014-01246 | 2014-01247 | 2014-01248 | 2014-01249 | 2014-01250 | 2014-01251 | 2014-01252 | 2014-01253 | 2014-01254 | 2014-01255 | 2014-01256 | 2014-01257 | 2014-01258 | 2014-01259 | 2014-01260 | 2014-01261 | 2014-01262 | 2014-01263 | 2014-01264 | 2014-01265 | 2014-01266 | 2014-01267 | 2014-01268 | 2014-01269 | 2014-01270 | 2014-01271 | 2014-01272 | 2014-01273 | 2014-01274 | 2014-01275 | 2014-01276 | 2014-01277 | 2014-01278 | 2014-01279 | 2014-01280 | 2014-01281 | 2014-01282 | 2014-01283 | 2014-01284 | 2014-01285 | 2014-01286 | 2014-01287 | 2014-01288 | 2014-01289 | 2014-01290 | 2014-01291; **Taxon:** kingdom: Animalia; phylum: Arthropoda; class: Insecta; order: Hemiptera; family: Lygaeidae; genus: Nysius; specificEpithet: sp.; **Location:** country: Japan; stateProvince: Tokyo; municipality: Meguro-ku; locality: The University of Tokyo Campus, Komaba.; minimumElevationInMeters: 31; maximumElevationInMeters: 39; decimalLatitude: 35.66006; decimalLongitude: 139.68521; geodeticDatum: WGS84; **Identification:** identifiedBy: T. Ishikawa; dateIdentified: 2013; **Event:** samplingProtocol: net sweeping; eventDate: 2013-06-22/2013-06-23; **Record Level:** institutionCode: KMUT; collectionCode: IC
- **Type status:** Other material. **Occurrence:** recordedBy: T. Ishikawa; individualCount: 45; sex: 31 males, 14 females; lifeStage: adult; otherCatalogNumbers: 2014-01292 | 2014-01293 | 2014-01294 | 2014-01295 | 2014-01296 | 2014-01297 | 2014-01298 | 2014-01299 | 2014-01300 | 2014-01301 | 2014-01302 | 2014-01303 | 2014-01304 | 2014-01305 | 2014-01306 | 2014-01307 | 2014-01308 | 2014-01309 | 2014-01310 | 2014-01311 | 2014-01312 | 2014-01313 | 2014-01314 | 2014-01315 | 2014-01316 | 2014-01317 | 2014-01318 | 2014-01319 | 2014-01320 | 2014-01321 | 2014-01322 | 2014-01323 | 2014-01324 | 2014-01325 | 2014-01326 | 2014-01327 | 2014-01328 | 2014-01329 | 2014-01330 | 2014-01331 | 2014-01332 | 2014-01333 | 2014-01334 | 2014-01335 | 2014-01336; **Taxon:** kingdom: Animalia; phylum: Arthropoda; class: Insecta; order: Hemiptera; family: Lygaeidae; genus: Nysius; specificEpithet: sp.; **Location:** country: Japan; stateProvince: Tokyo; municipality: Meguro-ku; locality: The University of Tokyo Campus, Komaba.; minimumElevationInMeters: 31; maximumElevationInMeters: 39; decimalLatitude: 35.66006; decimalLongitude: 139.68521; geodeticDatum: WGS84; **Identification:** identifiedBy: T. Ishikawa; dateIdentified: 2013; **Event:** samplingProtocol: net sweeping; eventDate: 2013-08-15; **Record Level:** institutionCode: KMUT; collectionCode: IC
- **Type status:** Other material. **Occurrence:** recordedBy: T. Ishikawa; individualCount: 1; sex: 1 female; lifeStage: adult; otherCatalogNumbers: 2014-01337; **Taxon:** kingdom: Animalia; phylum: Arthropoda; class: Insecta; order: Hemiptera; family: Lygaeidae; genus: Nysius; specificEpithet: sp.; **Location:** country: Japan; stateProvince: Tokyo; municipality: Meguro-ku; locality: The University of Tokyo Campus, Komaba.; minimumElevationInMeters: 31; maximumElevationInMeters: 39; decimalLatitude: 35.66006; decimalLongitude: 139.68521; geodeticDatum: WGS84; **Identification:** identifiedBy: T. Ishikawa; dateIdentified: 2013; **Event:** samplingProtocol: net sweeping; eventDate: 2013-09-06; **Record Level:** institutionCode: KMUT; collectionCode: IC
- **Type status:** Other material. **Occurrence:** recordedBy: T. Ishikawa; individualCount: 18; sex: 14 males, 4 females; lifeStage: adult; otherCatalogNumbers: 2014-01338 | 2014-01339 | 2014-01340 | 2014-01341 | 2014-01342 | 2014-01343 | 2014-01344 | 2014-01345 | 2014-01346 | 2014-01347 | 2014-01348 | 2014-01349 | 2014-01350 | 2014-01351 | 2014-01352 | 2014-01353 | 2014-01354 | 2014-01355; **Taxon:** kingdom: Animalia; phylum: Arthropoda; class: Insecta; order: Hemiptera; family: Lygaeidae; genus: Nysius; specificEpithet: sp.; **Location:** country: Japan; stateProvince: Tokyo; municipality: Meguro-ku; locality: The University of Tokyo Campus, Komaba.; minimumElevationInMeters: 31; maximumElevationInMeters: 39; decimalLatitude: 35.66006; decimalLongitude: 139.68521; geodeticDatum: WGS84; **Identification:** identifiedBy: T. Ishikawa; dateIdentified: 2013; **Event:** samplingProtocol: net sweeping; eventDate: 2013-10-30; **Record Level:** institutionCode: KMUT; collectionCode: IC

##### Notes

Correspond to an undescribed species listed as “*Nysius* sp. 1” in Ishikawa et al. (2012).

#### Malcidae

Stål, 1865

#### Chauliops fallax

Scott, 1874

##### Materials

- **Type status:** Other material. **Occurrence:** recordedBy: T. Ishikawa; individualCount: 6; sex: 1 male, 5 females; lifeStage: adult; otherCatalogNumbers: 2014-01356 | 2014-01357 | 2014-01358 | 2014-01359 | 2014-01360 | 2014-01361; **Taxon:** namePublishedIn: 1874; kingdom: Animalia; phylum: Arthropoda; class: Insecta; order: Hemiptera; family: Malcidae; genus: Chauliops; specificEpithet: fallax; scientificNameAuthorship: Scott; **Location:** country: Japan; stateProvince: Tokyo; municipality: Meguro-ku; locality: The University of Tokyo Campus, Komaba.; minimumElevationInMeters: 31; maximumElevationInMeters: 39; decimalLatitude: 35.66006; decimalLongitude: 139.68521; geodeticDatum: WGS84; **Identification:** identifiedBy: T. Ishikawa; dateIdentified: 2013; **Event:** samplingProtocol: net sweeping; eventDate: 2013-04-28/2013-04-29; **Record Level:** institutionCode: KMUT; collectionCode: IC
- **Type status:** Other material. **Occurrence:** recordedBy: T. Ishikawa; individualCount: 16; sex: 10 males, 6 females; lifeStage: adult; otherCatalogNumbers: 2014-01362 | 2014-01363 | 2014-01364 | 2014-01365 | 2014-01366 | 2014-01367 | 2014-01368 | 2014-01369 | 2014-01370 | 2014-01371 | 2014-01372 | 2014-01373 | 2014-01374 | 2014-01375 | 2014-01376 | 2014-01377; **Taxon:** namePublishedIn: 1874; kingdom: Animalia; phylum: Arthropoda; class: Insecta; order: Hemiptera; family: Malcidae; genus: Chauliops; specificEpithet: fallax; scientificNameAuthorship: Scott; **Location:** country: Japan; stateProvince: Tokyo; municipality: Meguro-ku; locality: The University of Tokyo Campus, Komaba.; minimumElevationInMeters: 31; maximumElevationInMeters: 39; decimalLatitude: 35.66006; decimalLongitude: 139.68521; geodeticDatum: WGS84; **Identification:** identifiedBy: T. Ishikawa; dateIdentified: 2013; **Event:** samplingProtocol: net sweeping; eventDate: 2013-05-12/2013-05-18; **Record Level:** institutionCode: KMUT; collectionCode: IC
- **Type status:** Other material. **Occurrence:** recordedBy: T. Ishikawa; individualCount: 2; sex: 2 females; lifeStage: adult; otherCatalogNumbers: 2014-01378 | 2014-01379; **Taxon:** namePublishedIn: 1874; kingdom: Animalia; phylum: Arthropoda; class: Insecta; order: Hemiptera; family: Malcidae; genus: Chauliops; specificEpithet: fallax; scientificNameAuthorship: Scott; **Location:** country: Japan; stateProvince: Tokyo; municipality: Meguro-ku; locality: The University of Tokyo Campus, Komaba.; minimumElevationInMeters: 31; maximumElevationInMeters: 39; decimalLatitude: 35.66006; decimalLongitude: 139.68521; geodeticDatum: WGS84; **Identification:** identifiedBy: T. Ishikawa; dateIdentified: 2013; **Event:** samplingProtocol: net sweeping; eventDate: 2013-06-22/2013-06-23; **Record Level:** institutionCode: KMUT; collectionCode: IC
- **Type status:** Other material. **Occurrence:** recordedBy: T. Ishikawa; individualCount: 5; sex: 1 male, 4 females; lifeStage: adult; otherCatalogNumbers: 2014-01380 | 2014-01381 | 2014-01382 | 2014-01383 | 2014-01384; **Taxon:** namePublishedIn: 1874; kingdom: Animalia; phylum: Arthropoda; class: Insecta; order: Hemiptera; family: Malcidae; genus: Chauliops; specificEpithet: fallax; scientificNameAuthorship: Scott; **Location:** country: Japan; stateProvince: Tokyo; municipality: Meguro-ku; locality: The University of Tokyo Campus, Komaba.; minimumElevationInMeters: 31; maximumElevationInMeters: 39; decimalLatitude: 35.66006; decimalLongitude: 139.68521; geodeticDatum: WGS84; **Identification:** identifiedBy: T. Ishikawa; dateIdentified: 2013; **Event:** samplingProtocol: net sweeping; eventDate: 2013-10-30; **Record Level:** institutionCode: KMUT; collectionCode: IC

#### Berytidae

Fieber, 1851

#### Metacanthus pulchellus

Dallas, 1852

##### Materials

- **Type status:** Other material. **Occurrence:** recordedBy: T. Ishikawa; individualCount: 1; sex: 1 male; lifeStage: adult; otherCatalogNumbers: 2014-01385; **Taxon:** namePublishedIn: 1852; kingdom: Animalia; phylum: Arthropoda; class: Insecta; order: Hemiptera; family: Berytidae; genus: Metacanthus; specificEpithet: pulchellus; scientificNameAuthorship: Dallas; **Location:** country: Japan; stateProvince: Tokyo; municipality: Meguro-ku; locality: The University of Tokyo Campus, Komaba.; minimumElevationInMeters: 31; maximumElevationInMeters: 39; decimalLatitude: 35.66006; decimalLongitude: 139.68521; geodeticDatum: WGS84; **Identification:** identifiedBy: T. Ishikawa; dateIdentified: 2013; **Event:** samplingProtocol: net sweeping; eventDate: 2013-08-18; **Record Level:** institutionCode: KMUT; collectionCode: IC
- **Type status:** Other material. **Occurrence:** recordedBy: T. Ishikawa; individualCount: 2; sex: 1 male, 1 female; lifeStage: adult; otherCatalogNumbers: 2014-01386 | 2014-01387; **Taxon:** namePublishedIn: 1852; kingdom: Animalia; phylum: Arthropoda; class: Insecta; order: Hemiptera; family: Berytidae; genus: Metacanthus; specificEpithet: pulchellus; scientificNameAuthorship: Dallas; **Location:** country: Japan; stateProvince: Tokyo; municipality: Meguro-ku; locality: The University of Tokyo Campus, Komaba.; minimumElevationInMeters: 31; maximumElevationInMeters: 39; decimalLatitude: 35.66006; decimalLongitude: 139.68521; geodeticDatum: WGS84; **Identification:** identifiedBy: T. Ishikawa; dateIdentified: 2013; **Event:** samplingProtocol: net sweeping; eventDate: 2013-10-30; **Record Level:** institutionCode: KMUT; collectionCode: IC

#### Yemma exilis

Horváth, 1905

##### Materials

- **Type status:** Other material. **Occurrence:** recordedBy: T. Ishikawa; individualCount: 9; sex: 7 males, 2 females; lifeStage: adult; otherCatalogNumbers: 2014-01388 | 2014-01389 | 2014-01390 | 2014-01391 | 2014-01392 | 2014-01393 | 2014-01394 | 2014-01395 | 2014-01396; **Taxon:** namePublishedIn: 1905; kingdom: Animalia; phylum: Arthropoda; class: Insecta; order: Hemiptera; family: Berytidae; genus: Yemma; specificEpithet: exilis; scientificNameAuthorship: Horváth; **Location:** country: Japan; stateProvince: Tokyo; municipality: Meguro-ku; locality: The University of Tokyo Campus, Komaba.; minimumElevationInMeters: 31; maximumElevationInMeters: 39; decimalLatitude: 35.66006; decimalLongitude: 139.68521; geodeticDatum: WGS84; **Identification:** identifiedBy: T. Ishikawa; dateIdentified: 2013; **Event:** samplingProtocol: net sweeping; eventDate: 2013-04-28; **Record Level:** institutionCode: KMUT; collectionCode: IC
- **Type status:** Other material. **Occurrence:** recordedBy: T. Ishikawa; individualCount: 3; sex: 1 male, 2 females; lifeStage: adult; otherCatalogNumbers: 2014-01397 | 2014-01398 | 2014-01399; **Taxon:** namePublishedIn: 1905; kingdom: Animalia; phylum: Arthropoda; class: Insecta; order: Hemiptera; family: Berytidae; genus: Yemma; specificEpithet: exilis; scientificNameAuthorship: Horváth; **Location:** country: Japan; stateProvince: Tokyo; municipality: Meguro-ku; locality: The University of Tokyo Campus, Komaba.; minimumElevationInMeters: 31; maximumElevationInMeters: 39; decimalLatitude: 35.66006; decimalLongitude: 139.68521; geodeticDatum: WGS84; **Identification:** identifiedBy: T. Ishikawa; dateIdentified: 2013; **Event:** samplingProtocol: net sweeping; eventDate: 2013-05-18; **Record Level:** institutionCode: KMUT; collectionCode: IC
- **Type status:** Other material. **Occurrence:** recordedBy: T. Ishikawa; individualCount: 1; sex: 1 female; lifeStage: adult; otherCatalogNumbers: 2014-01400; **Taxon:** namePublishedIn: 1905; kingdom: Animalia; phylum: Arthropoda; class: Insecta; order: Hemiptera; family: Berytidae; genus: Yemma; specificEpithet: exilis; scientificNameAuthorship: Horváth; **Location:** country: Japan; stateProvince: Tokyo; municipality: Meguro-ku; locality: The University of Tokyo Campus, Komaba.; minimumElevationInMeters: 31; maximumElevationInMeters: 39; decimalLatitude: 35.66006; decimalLongitude: 139.68521; geodeticDatum: WGS84; **Identification:** identifiedBy: T. Ishikawa; dateIdentified: 2013; **Event:** samplingProtocol: net sweeping; eventDate: 2013-05-25; **Record Level:** institutionCode: KMUT; collectionCode: IC
- **Type status:** Other material. **Occurrence:** recordedBy: T. Ishikawa; individualCount: 4; sex: 1 male, 3 females; lifeStage: adult; otherCatalogNumbers: 2014-01401 | 2014-01402 | 2014-01403 | 2014-01404; **Taxon:** namePublishedIn: 1905; kingdom: Animalia; phylum: Arthropoda; class: Insecta; order: Hemiptera; family: Berytidae; genus: Yemma; specificEpithet: exilis; scientificNameAuthorship: Horváth; **Location:** country: Japan; stateProvince: Tokyo; municipality: Meguro-ku; locality: The University of Tokyo Campus, Komaba.; minimumElevationInMeters: 31; maximumElevationInMeters: 39; decimalLatitude: 35.66006; decimalLongitude: 139.68521; geodeticDatum: WGS84; **Identification:** identifiedBy: T. Ishikawa; dateIdentified: 2013; **Event:** samplingProtocol: net sweeping; eventDate: 2013-06-16/2013-06-22; **Record Level:** institutionCode: KMUT; collectionCode: IC
- **Type status:** Other material. **Occurrence:** recordedBy: T. Ishikawa; individualCount: 1; sex: 1 male; lifeStage: adult; otherCatalogNumbers: 2014-01405; **Taxon:** namePublishedIn: 1905; kingdom: Animalia; phylum: Arthropoda; class: Insecta; order: Hemiptera; family: Berytidae; genus: Yemma; specificEpithet: exilis; scientificNameAuthorship: Horváth; **Location:** country: Japan; stateProvince: Tokyo; municipality: Meguro-ku; locality: The University of Tokyo Campus, Komaba.; minimumElevationInMeters: 31; maximumElevationInMeters: 39; decimalLatitude: 35.66006; decimalLongitude: 139.68521; geodeticDatum: WGS84; **Identification:** identifiedBy: T. Ishikawa; dateIdentified: 2013; **Event:** samplingProtocol: light trap; eventDate: 2013-08-02; **Record Level:** institutionCode: KMUT; collectionCode: IC
- **Type status:** Other material. **Occurrence:** recordedBy: T. Ishikawa; individualCount: 1; sex: 1 female; lifeStage: adult; otherCatalogNumbers: 2014-01406; **Taxon:** namePublishedIn: 1905; kingdom: Animalia; phylum: Arthropoda; class: Insecta; order: Hemiptera; family: Berytidae; genus: Yemma; specificEpithet: exilis; scientificNameAuthorship: Horváth; **Location:** country: Japan; stateProvince: Tokyo; municipality: Meguro-ku; locality: The University of Tokyo Campus, Komaba.; minimumElevationInMeters: 31; maximumElevationInMeters: 39; decimalLatitude: 35.66006; decimalLongitude: 139.68521; geodeticDatum: WGS84; **Identification:** identifiedBy: T. Ishikawa; dateIdentified: 2013; **Event:** samplingProtocol: net sweeping; eventDate: 2013-10-30; **Record Level:** institutionCode: KMUT; collectionCode: IC

#### Pyrrhocoroidea

Amyot et Serville, 1843

#### Largidae

Amyot et Serville, 1843

#### Physopelta gutta

(Burmeister, 1834)

##### Materials

- **Type status:** Other material. **Occurrence:** recordedBy: T. Ishikawa; individualCount: 1; sex: 1 male; lifeStage: adult; otherCatalogNumbers: 2014-01409; **Taxon:** namePublishedIn: 1834; kingdom: Animalia; phylum: Arthropoda; class: Insecta; order: Hemiptera; family: Largidae; genus: Physopelta; specificEpithet: gutta; scientificNameAuthorship: Burmeister; **Location:** country: Japan; stateProvince: Tokyo; municipality: Meguro-ku; locality: The University of Tokyo Campus, Komaba.; minimumElevationInMeters: 31; maximumElevationInMeters: 39; decimalLatitude: 35.66006; decimalLongitude: 139.68521; geodeticDatum: WGS84; **Identification:** identifiedBy: T. Ishikawa; dateIdentified: 2013; **Event:** samplingProtocol: net sweeping; eventDate: 2013-06-16; **Record Level:** institutionCode: KMUT; collectionCode: IC
- **Type status:** Other material. **Occurrence:** recordedBy: N. Utsuki; individualCount: 1; sex: 1 female; lifeStage: adult; otherCatalogNumbers: 2014-01410; **Taxon:** namePublishedIn: 1834; kingdom: Animalia; phylum: Arthropoda; class: Insecta; order: Hemiptera; family: Largidae; genus: Physopelta; specificEpithet: gutta; scientificNameAuthorship: Burmeister; **Location:** country: Japan; stateProvince: Tokyo; municipality: Meguro-ku; locality: The University of Tokyo Campus, Komaba.; minimumElevationInMeters: 31; maximumElevationInMeters: 39; decimalLatitude: 35.66006; decimalLongitude: 139.68521; geodeticDatum: WGS84; **Identification:** identifiedBy: T. Ishikawa; dateIdentified: 2013; **Event:** samplingProtocol: net sweeping; eventDate: 2013-08-29; **Record Level:** institutionCode: KMUT; collectionCode: IC
- **Type status:** Other material. **Occurrence:** recordedBy: T. Ishikawa; individualCount: 1; sex: 1 male; lifeStage: adult; otherCatalogNumbers: 2014-01411; **Taxon:** namePublishedIn: 1834; kingdom: Animalia; phylum: Arthropoda; class: Insecta; order: Hemiptera; family: Largidae; genus: Physopelta; specificEpithet: gutta; scientificNameAuthorship: Burmeister; **Location:** country: Japan; stateProvince: Tokyo; municipality: Meguro-ku; locality: The University of Tokyo Campus, Komaba.; minimumElevationInMeters: 31; maximumElevationInMeters: 39; decimalLatitude: 35.66006; decimalLongitude: 139.68521; geodeticDatum: WGS84; **Identification:** identifiedBy: T. Ishikawa; dateIdentified: 2013; **Event:** samplingProtocol: net sweeping; eventDate: 2013-10-30; **Record Level:** institutionCode: KMUT; collectionCode: IC

#### Physopelta parviceps

Blöte, 1931

##### Materials

- **Type status:** Other material. **Occurrence:** recordedBy: T. Ishikawa; individualCount: 1; sex: 1 male; lifeStage: adult; otherCatalogNumbers: 2014-01407; **Taxon:** namePublishedIn: 1931; kingdom: Animalia; phylum: Arthropoda; class: Insecta; order: Hemiptera; family: Largidae; genus: Physopelta; specificEpithet: parviceps; scientificNameAuthorship: Blöte; **Location:** country: Japan; stateProvince: Tokyo; municipality: Meguro-ku; locality: The University of Tokyo Campus, Komaba.; minimumElevationInMeters: 31; maximumElevationInMeters: 39; decimalLatitude: 35.66006; decimalLongitude: 139.68521; geodeticDatum: WGS84; **Identification:** identifiedBy: T. Ishikawa; dateIdentified: 2013; **Event:** samplingProtocol: net sweeping; eventDate: 2013-06-18; **Record Level:** institutionCode: KMUT; collectionCode: IC
- **Type status:** Other material. **Occurrence:** recordedBy: T. Ishikawa; individualCount: 1; sex: 1 male; lifeStage: adult; otherCatalogNumbers: 2014-01408; **Taxon:** namePublishedIn: 1931; kingdom: Animalia; phylum: Arthropoda; class: Insecta; order: Hemiptera; family: Largidae; genus: Physopelta; specificEpithet: parviceps; scientificNameAuthorship: Blöte; **Location:** country: Japan; stateProvince: Tokyo; municipality: Meguro-ku; locality: The University of Tokyo Campus, Komaba.; minimumElevationInMeters: 31; maximumElevationInMeters: 39; decimalLatitude: 35.66006; decimalLongitude: 139.68521; geodeticDatum: WGS84; **Identification:** identifiedBy: T. Ishikawa; dateIdentified: 2013; **Event:** samplingProtocol: net sweeping; eventDate: 2013-08-20; **Record Level:** institutionCode: KMUT; collectionCode: IC

#### Pyrrhocoridae

Amyot et Serville, 1843

#### Pyrrhocoris sibiricus

Kuschakewitsch, 1866

##### Materials

- **Type status:** Other material. **Occurrence:** recordedBy: T. Ishikawa; individualCount: 1; sex: 1 male; lifeStage: adult; otherCatalogNumbers: 2014-01412; **Taxon:** namePublishedIn: 1866; kingdom: Animalia; phylum: Arthropoda; class: Insecta; order: Hemiptera; family: Pyrrhocoridae; genus: Pyrrhocoris; specificEpithet: sibiricus; scientificNameAuthorship: Kuschakewitsch; **Location:** country: Japan; stateProvince: Tokyo; municipality: Meguro-ku; locality: The University of Tokyo Campus, Komaba.; minimumElevationInMeters: 31; maximumElevationInMeters: 39; decimalLatitude: 35.66006; decimalLongitude: 139.68521; geodeticDatum: WGS84; **Identification:** identifiedBy: T. Ishikawa; dateIdentified: 2013; **Event:** samplingProtocol: net sweeping; eventDate: 2013-09-03; **Record Level:** institutionCode: KMUT; collectionCode: IC
- **Type status:** Other material. **Occurrence:** recordedBy: T. Ishikawa; individualCount: 1; sex: 1 female; lifeStage: adult; otherCatalogNumbers: 2014-01413; **Taxon:** namePublishedIn: 1866; kingdom: Animalia; phylum: Arthropoda; class: Insecta; order: Hemiptera; family: Pyrrhocoridae; genus: Pyrrhocoris; specificEpithet: sibiricus; scientificNameAuthorship: Kuschakewitsch; **Location:** country: Japan; stateProvince: Tokyo; municipality: Meguro-ku; locality: The University of Tokyo Campus, Komaba.; minimumElevationInMeters: 31; maximumElevationInMeters: 39; decimalLatitude: 35.66006; decimalLongitude: 139.68521; geodeticDatum: WGS84; **Identification:** identifiedBy: T. Ishikawa; dateIdentified: 2013; **Event:** samplingProtocol: net sweeping; eventDate: 2013-09-19; **Record Level:** institutionCode: KMUT; collectionCode: IC

#### Coreoidea

Leach, 1815

#### Alydidae

Amyot et Serville, 1843

#### Leptocorisa chinensis

Dallas, 1852

##### Materials

- **Type status:** Other material. **Occurrence:** recordedBy: T. Ishikawa; individualCount: 4; sex: 4 females; lifeStage: adult; otherCatalogNumbers: 2014-01414 | 2014-01415 | 2014-01416 | 2014-01417; **Taxon:** namePublishedIn: 1852; kingdom: Animalia; phylum: Arthropoda; class: Insecta; order: Hemiptera; family: Alydidae; genus: Leptocorisa; specificEpithet: chinensis; scientificNameAuthorship: Dallas; **Location:** country: Japan; stateProvince: Tokyo; municipality: Meguro-ku; locality: The University of Tokyo Campus, Komaba.; minimumElevationInMeters: 31; maximumElevationInMeters: 39; decimalLatitude: 35.66006; decimalLongitude: 139.68521; geodeticDatum: WGS84; **Identification:** identifiedBy: T. Ishikawa; dateIdentified: 2013; **Event:** samplingProtocol: net sweeping; eventDate: 2013-09-06; **Record Level:** institutionCode: KMUT; collectionCode: IC

#### Paraplesius vulgaris

(Hsiao, 1964)

##### Materials

- **Type status:** Other material. **Occurrence:** recordedBy: T. Ishikawa; individualCount: 2; sex: 2 males; lifeStage: adult; otherCatalogNumbers: 2014-01418 | 2014-01419; **Taxon:** namePublishedIn: 1964; kingdom: Animalia; phylum: Arthropoda; class: Insecta; order: Hemiptera; family: Alydidae; genus: Paraplesius; specificEpithet: vulgaris; scientificNameAuthorship: Hsiao; **Location:** country: Japan; stateProvince: Tokyo; municipality: Meguro-ku; locality: The University of Tokyo Campus, Komaba.; minimumElevationInMeters: 31; maximumElevationInMeters: 39; decimalLatitude: 35.66006; decimalLongitude: 139.68521; geodeticDatum: WGS84; **Identification:** identifiedBy: T. Ishikawa; dateIdentified: 2013; **Event:** samplingProtocol: net sweeping; eventDate: 2013-05-19; **Record Level:** institutionCode: KMUT; collectionCode: IC
- **Type status:** Other material. **Occurrence:** recordedBy: T. Ishikawa; individualCount: 1; sex: 1 male; lifeStage: adult; otherCatalogNumbers: 2014-01420; **Taxon:** namePublishedIn: 1964; kingdom: Animalia; phylum: Arthropoda; class: Insecta; order: Hemiptera; family: Alydidae; genus: Paraplesius; specificEpithet: vulgaris; scientificNameAuthorship: Hsiao; **Location:** country: Japan; stateProvince: Tokyo; municipality: Meguro-ku; locality: The University of Tokyo Campus, Komaba.; minimumElevationInMeters: 31; maximumElevationInMeters: 39; decimalLatitude: 35.66006; decimalLongitude: 139.68521; geodeticDatum: WGS84; **Identification:** identifiedBy: T. Ishikawa; dateIdentified: 2013; **Event:** samplingProtocol: net sweeping; eventDate: 2013-09-06; **Record Level:** institutionCode: KMUT; collectionCode: IC

##### Notes

First record in Tokyo.

#### Riptortus pedestris

(Fabricius, 1775)

##### Materials

- **Type status:** Other material. **Occurrence:** recordedBy: T. Ishikawa; individualCount: 1; sex: 1 female; lifeStage: adult; otherCatalogNumbers: 2014-01421; **Taxon:** namePublishedIn: 1775; kingdom: Animalia; phylum: Arthropoda; class: Insecta; order: Hemiptera; family: Alydidae; genus: Riptortus; specificEpithet: pedestris; scientificNameAuthorship: Fabricius; **Location:** country: Japan; stateProvince: Tokyo; municipality: Meguro-ku; locality: The University of Tokyo Campus, Komaba.; minimumElevationInMeters: 31; maximumElevationInMeters: 39; decimalLatitude: 35.66006; decimalLongitude: 139.68521; geodeticDatum: WGS84; **Identification:** identifiedBy: T. Ishikawa; dateIdentified: 2013; **Event:** samplingProtocol: net sweeping; eventDate: 2013-05-18; **Record Level:** institutionCode: KMUT; collectionCode: IC
- **Type status:** Other material. **Occurrence:** recordedBy: T. Ishikawa; individualCount: 1; sex: 1 male; lifeStage: adult; otherCatalogNumbers: 2014-01422; **Taxon:** namePublishedIn: 1775; kingdom: Animalia; phylum: Arthropoda; class: Insecta; order: Hemiptera; family: Alydidae; genus: Riptortus; specificEpithet: pedestris; scientificNameAuthorship: Fabricius; **Location:** country: Japan; stateProvince: Tokyo; municipality: Meguro-ku; locality: The University of Tokyo Campus, Komaba.; minimumElevationInMeters: 31; maximumElevationInMeters: 39; decimalLatitude: 35.66006; decimalLongitude: 139.68521; geodeticDatum: WGS84; **Identification:** identifiedBy: T. Ishikawa; dateIdentified: 2013; **Event:** samplingProtocol: net sweeping; eventDate: 2013-06-22; **Record Level:** institutionCode: KMUT; collectionCode: IC

#### Rhopalidae

Amyot et Serville, 1843

#### Liorhyssus hyalinus

(Fabricius, 1794)

##### Materials

- **Type status:** Other material. **Occurrence:** recordedBy: T. Ishikawa; individualCount: 1; sex: 1 male; lifeStage: adult; otherCatalogNumbers: 2014-01423; **Taxon:** namePublishedIn: 1794; kingdom: Animalia; phylum: Arthropoda; class: Insecta; order: Hemiptera; family: Rhopalidae; genus: Liorhyssus; specificEpithet: hyalinus; scientificNameAuthorship: Fabricius; **Location:** country: Japan; stateProvince: Tokyo; municipality: Meguro-ku; locality: The University of Tokyo Campus, Komaba.; minimumElevationInMeters: 31; maximumElevationInMeters: 39; decimalLatitude: 35.66006; decimalLongitude: 139.68521; geodeticDatum: WGS84; **Identification:** identifiedBy: T. Ishikawa; dateIdentified: 2013; **Event:** samplingProtocol: net sweeping; eventDate: 2013-04-28; **Record Level:** institutionCode: KMUT; collectionCode: IC
- **Type status:** Other material. **Occurrence:** recordedBy: T. Ishikawa; individualCount: 1; sex: 1 male; lifeStage: adult; otherCatalogNumbers: 2014-01424; **Taxon:** namePublishedIn: 1794; kingdom: Animalia; phylum: Arthropoda; class: Insecta; order: Hemiptera; family: Rhopalidae; genus: Liorhyssus; specificEpithet: hyalinus; scientificNameAuthorship: Fabricius; **Location:** country: Japan; stateProvince: Tokyo; municipality: Meguro-ku; locality: The University of Tokyo Campus, Komaba.; minimumElevationInMeters: 31; maximumElevationInMeters: 39; decimalLatitude: 35.66006; decimalLongitude: 139.68521; geodeticDatum: WGS84; **Identification:** identifiedBy: T. Ishikawa; dateIdentified: 2013; **Event:** samplingProtocol: net sweeping; eventDate: 2013-06-23; **Record Level:** institutionCode: KMUT; collectionCode: IC
- **Type status:** Other material. **Occurrence:** recordedBy: T. Ishikawa; individualCount: 3; sex: 3 males; lifeStage: adult; otherCatalogNumbers: 2014-01425 | 2014-01426 | 2014-01427; **Taxon:** namePublishedIn: 1794; kingdom: Animalia; phylum: Arthropoda; class: Insecta; order: Hemiptera; family: Rhopalidae; genus: Liorhyssus; specificEpithet: hyalinus; scientificNameAuthorship: Fabricius; **Location:** country: Japan; stateProvince: Tokyo; municipality: Meguro-ku; locality: The University of Tokyo Campus, Komaba.; minimumElevationInMeters: 31; maximumElevationInMeters: 39; decimalLatitude: 35.66006; decimalLongitude: 139.68521; geodeticDatum: WGS84; **Identification:** identifiedBy: T. Ishikawa; dateIdentified: 2013; **Event:** samplingProtocol: net sweeping; eventDate: 2013-08-15; **Record Level:** institutionCode: KMUT; collectionCode: IC
- **Type status:** Other material. **Occurrence:** recordedBy: T. Ishikawa; individualCount: 1; sex: 1 male; lifeStage: adult; otherCatalogNumbers: 2014-01428; **Taxon:** namePublishedIn: 1794; kingdom: Animalia; phylum: Arthropoda; class: Insecta; order: Hemiptera; family: Rhopalidae; genus: Liorhyssus; specificEpithet: hyalinus; scientificNameAuthorship: Fabricius; **Location:** country: Japan; stateProvince: Tokyo; municipality: Meguro-ku; locality: The University of Tokyo Campus, Komaba.; minimumElevationInMeters: 31; maximumElevationInMeters: 39; decimalLatitude: 35.66006; decimalLongitude: 139.68521; geodeticDatum: WGS84; **Identification:** identifiedBy: T. Ishikawa; dateIdentified: 2013; **Event:** samplingProtocol: net sweeping; eventDate: 2013-09-06; **Record Level:** institutionCode: KMUT; collectionCode: IC
- **Type status:** Other material. **Occurrence:** recordedBy: T. Ishikawa; individualCount: 1; sex: 1 female; lifeStage: adult; otherCatalogNumbers: 2014-01429; **Taxon:** namePublishedIn: 1794; kingdom: Animalia; phylum: Arthropoda; class: Insecta; order: Hemiptera; family: Rhopalidae; genus: Liorhyssus; specificEpithet: hyalinus; scientificNameAuthorship: Fabricius; **Location:** country: Japan; stateProvince: Tokyo; municipality: Meguro-ku; locality: The University of Tokyo Campus, Komaba.; minimumElevationInMeters: 31; maximumElevationInMeters: 39; decimalLatitude: 35.66006; decimalLongitude: 139.68521; geodeticDatum: WGS84; **Identification:** identifiedBy: T. Ishikawa; dateIdentified: 2013; **Event:** samplingProtocol: net sweeping; eventDate: 2013-10-30; **Record Level:** institutionCode: KMUT; collectionCode: IC

##### Notes

First record in Tokyo.

#### Rhopalus maculatus

(Fieber, 1837)

##### Materials

- **Type status:** Other material. **Occurrence:** recordedBy: T. Ishikawa; individualCount: 1; sex: 1 male; lifeStage: adult; otherCatalogNumbers: 2014-01430; **Taxon:** namePublishedIn: 1837; kingdom: Animalia; phylum: Arthropoda; class: Insecta; order: Hemiptera; family: Rhopalidae; genus: Rhopalus; specificEpithet: maculatus; scientificNameAuthorship: Fieber; **Location:** country: Japan; stateProvince: Tokyo; municipality: Meguro-ku; locality: The University of Tokyo Campus, Komaba.; minimumElevationInMeters: 31; maximumElevationInMeters: 39; decimalLatitude: 35.66006; decimalLongitude: 139.68521; geodeticDatum: WGS84; **Identification:** identifiedBy: T. Ishikawa; dateIdentified: 2013; **Event:** samplingProtocol: net sweeping; eventDate: 2013-08-15; **Record Level:** institutionCode: KMUT; collectionCode: IC

#### Stictopleurus punctatonervosus

(Goeze, 1778)

##### Materials

- **Type status:** Other material. **Occurrence:** recordedBy: T. Ishikawa; individualCount: 1; sex: 1 female; lifeStage: adult; otherCatalogNumbers: 2014-01431; **Taxon:** namePublishedIn: 1778; kingdom: Animalia; phylum: Arthropoda; class: Insecta; order: Hemiptera; family: Rhopalidae; genus: Stictopleurus; specificEpithet: punctatonervosus; scientificNameAuthorship: Goeze; **Location:** country: Japan; stateProvince: Tokyo; municipality: Meguro-ku; locality: The University of Tokyo Campus, Komaba.; minimumElevationInMeters: 31; maximumElevationInMeters: 39; decimalLatitude: 35.66006; decimalLongitude: 139.68521; geodeticDatum: WGS84; **Identification:** identifiedBy: T. Ishikawa; dateIdentified: 2013; **Event:** samplingProtocol: net sweeping; eventDate: 2013-04-28; **Record Level:** institutionCode: KMUT; collectionCode: IC
- **Type status:** Other material. **Occurrence:** recordedBy: T. Ishikawa; individualCount: 3; sex: 2 males, 1 female; lifeStage: adult; otherCatalogNumbers: 2014-01432 | 2014-01433 | 2014-01434; **Taxon:** namePublishedIn: 1778; kingdom: Animalia; phylum: Arthropoda; class: Insecta; order: Hemiptera; family: Rhopalidae; genus: Stictopleurus; specificEpithet: punctatonervosus; scientificNameAuthorship: Goeze; **Location:** country: Japan; stateProvince: Tokyo; municipality: Meguro-ku; locality: The University of Tokyo Campus, Komaba.; minimumElevationInMeters: 31; maximumElevationInMeters: 39; decimalLatitude: 35.66006; decimalLongitude: 139.68521; geodeticDatum: WGS84; **Identification:** identifiedBy: T. Ishikawa; dateIdentified: 2013; **Event:** samplingProtocol: net sweeping; eventDate: 2013-06-23; **Record Level:** institutionCode: KMUT; collectionCode: IC

#### Coreidae

Leach, 1815

#### Acanthocoris sordidus

(Thunberg, 1783)

##### Materials

- **Type status:** Other material. **Occurrence:** recordedBy: T. Ishikawa; individualCount: 1; sex: 1 male; lifeStage: adult; otherCatalogNumbers: 2014-01435; **Taxon:** namePublishedIn: 1783; kingdom: Animalia; phylum: Arthropoda; class: Insecta; order: Hemiptera; family: Coreidae; genus: Acanthocoris; specificEpithet: sordidus; scientificNameAuthorship: Thunberg; **Location:** country: Japan; stateProvince: Tokyo; municipality: Meguro-ku; locality: The University of Tokyo Campus, Komaba.; minimumElevationInMeters: 31; maximumElevationInMeters: 39; decimalLatitude: 35.66006; decimalLongitude: 139.68521; geodeticDatum: WGS84; **Identification:** identifiedBy: T. Ishikawa; dateIdentified: 2013; **Event:** samplingProtocol: net sweeping; eventDate: 2013-05-12; **Record Level:** institutionCode: KMUT; collectionCode: IC
- **Type status:** Other material. **Occurrence:** recordedBy: T. Ishikawa; individualCount: 2; sex: 2 females; lifeStage: adult; otherCatalogNumbers: 2014-01436 | 2014-01437; **Taxon:** namePublishedIn: 1783; kingdom: Animalia; phylum: Arthropoda; class: Insecta; order: Hemiptera; family: Coreidae; genus: Acanthocoris; specificEpithet: sordidus; scientificNameAuthorship: Thunberg; **Location:** country: Japan; stateProvince: Tokyo; municipality: Meguro-ku; locality: The University of Tokyo Campus, Komaba.; minimumElevationInMeters: 31; maximumElevationInMeters: 39; decimalLatitude: 35.66006; decimalLongitude: 139.68521; geodeticDatum: WGS84; **Identification:** identifiedBy: T. Ishikawa; dateIdentified: 2014; **Event:** samplingProtocol: net sweeping; eventDate: 2014-05-01; **Record Level:** institutionCode: KMUT; collectionCode: IC

#### Cletus punctiger

(Dallas, 1852)

##### Materials

- **Type status:** Other material. **Occurrence:** recordedBy: T. Ishikawa; individualCount: 1; sex: 1 male; lifeStage: adult; otherCatalogNumbers: 2014-01441; **Taxon:** namePublishedIn: 1852; kingdom: Animalia; phylum: Arthropoda; class: Insecta; order: Hemiptera; family: Coreidae; genus: Cletus; specificEpithet: punctiger; scientificNameAuthorship: Dallas; **Location:** country: Japan; stateProvince: Tokyo; municipality: Meguro-ku; locality: The University of Tokyo Campus, Komaba.; minimumElevationInMeters: 31; maximumElevationInMeters: 39; decimalLatitude: 35.66006; decimalLongitude: 139.68521; geodeticDatum: WGS84; **Identification:** identifiedBy: T. Ishikawa; dateIdentified: 2013; **Event:** samplingProtocol: net sweeping; eventDate: 2013-08-15; **Record Level:** institutionCode: KMUT; collectionCode: IC

#### Cletus schmidti

Kiritshenko, 1916

##### Materials

- **Type status:** Other material. **Occurrence:** recordedBy: T. Ishikawa; individualCount: 1; sex: 1 female; lifeStage: adult; otherCatalogNumbers: 2014-01442; **Taxon:** namePublishedIn: 1916; kingdom: Animalia; phylum: Arthropoda; class: Insecta; order: Hemiptera; family: Coreidae; genus: Cletus; specificEpithet: schmidti; scientificNameAuthorship: Kiritshenko; **Location:** country: Japan; stateProvince: Tokyo; municipality: Meguro-ku; locality: The University of Tokyo Campus, Komaba.; minimumElevationInMeters: 31; maximumElevationInMeters: 39; decimalLatitude: 35.66006; decimalLongitude: 139.68521; geodeticDatum: WGS84; **Identification:** identifiedBy: T. Ishikawa; dateIdentified: 2013; **Event:** samplingProtocol: net sweeping; eventDate: 2013-05-12; **Record Level:** institutionCode: KMUT; collectionCode: IC
- **Type status:** Other material. **Occurrence:** recordedBy: T. Ishikawa; individualCount: 1; sex: 1 male; lifeStage: adult; otherCatalogNumbers: 2014-01443; **Taxon:** namePublishedIn: 1916; kingdom: Animalia; phylum: Arthropoda; class: Insecta; order: Hemiptera; family: Coreidae; genus: Cletus; specificEpithet: schmidti; scientificNameAuthorship: Kiritshenko; **Location:** country: Japan; stateProvince: Tokyo; municipality: Meguro-ku; locality: The University of Tokyo Campus, Komaba.; minimumElevationInMeters: 31; maximumElevationInMeters: 39; decimalLatitude: 35.66006; decimalLongitude: 139.68521; geodeticDatum: WGS84; **Identification:** identifiedBy: T. Ishikawa; dateIdentified: 2013; **Event:** samplingProtocol: net sweeping; eventDate: 2013-05-25; **Record Level:** institutionCode: KMUT; collectionCode: IC
- **Type status:** Other material. **Occurrence:** recordedBy: T. Ishikawa; individualCount: 1; sex: 1 female; lifeStage: adult; otherCatalogNumbers: 2014-01444; **Taxon:** namePublishedIn: 1916; kingdom: Animalia; phylum: Arthropoda; class: Insecta; order: Hemiptera; family: Coreidae; genus: Cletus; specificEpithet: schmidti; scientificNameAuthorship: Kiritshenko; **Location:** country: Japan; stateProvince: Tokyo; municipality: Meguro-ku; locality: The University of Tokyo Campus, Komaba.; minimumElevationInMeters: 31; maximumElevationInMeters: 39; decimalLatitude: 35.66006; decimalLongitude: 139.68521; geodeticDatum: WGS84; **Identification:** identifiedBy: T. Ishikawa; dateIdentified: 2013; **Event:** samplingProtocol: net sweeping; eventDate: 2013-06-22; **Record Level:** institutionCode: KMUT; collectionCode: IC
- **Type status:** Other material. **Occurrence:** recordedBy: T. Ishikawa; individualCount: 1; sex: 1 male; lifeStage: adult; otherCatalogNumbers: 2014-01445; **Taxon:** namePublishedIn: 1916; kingdom: Animalia; phylum: Arthropoda; class: Insecta; order: Hemiptera; family: Coreidae; genus: Cletus; specificEpithet: schmidti; scientificNameAuthorship: Kiritshenko; **Location:** country: Japan; stateProvince: Tokyo; municipality: Meguro-ku; locality: The University of Tokyo Campus, Komaba.; minimumElevationInMeters: 31; maximumElevationInMeters: 39; decimalLatitude: 35.66006; decimalLongitude: 139.68521; geodeticDatum: WGS84; **Identification:** identifiedBy: T. Ishikawa; dateIdentified: 2013; **Event:** samplingProtocol: net sweeping; eventDate: 2013-08-15; **Record Level:** institutionCode: KMUT; collectionCode: IC
- **Type status:** Other material. **Occurrence:** recordedBy: T. Ishikawa; individualCount: 4; sex: 2 males, 2 females; lifeStage: adult; otherCatalogNumbers: 2014-01446 | 2014-01447 | 2014-01448 | 2014-01449; **Taxon:** namePublishedIn: 1916; kingdom: Animalia; phylum: Arthropoda; class: Insecta; order: Hemiptera; family: Coreidae; genus: Cletus; specificEpithet: schmidti; scientificNameAuthorship: Kiritshenko; **Location:** country: Japan; stateProvince: Tokyo; municipality: Meguro-ku; locality: The University of Tokyo Campus, Komaba.; minimumElevationInMeters: 31; maximumElevationInMeters: 39; decimalLatitude: 35.66006; decimalLongitude: 139.68521; geodeticDatum: WGS84; **Identification:** identifiedBy: T. Ishikawa; dateIdentified: 2013; **Event:** samplingProtocol: net sweeping; eventDate: 2013-10-30; **Record Level:** institutionCode: KMUT; collectionCode: IC

#### Homoeocerus unipunctatus

(Thunberg, 1783)

##### Materials

- **Type status:** Other material. **Occurrence:** recordedBy: T. Ishikawa; individualCount: 1; sex: 1 female; lifeStage: adult; otherCatalogNumbers: 2014-01450; **Taxon:** namePublishedIn: 1783; kingdom: Animalia; phylum: Arthropoda; class: Insecta; order: Hemiptera; family: Coreidae; genus: Homoeocerus; specificEpithet: unipunctatus; scientificNameAuthorship: Thunberg; **Location:** country: Japan; stateProvince: Tokyo; municipality: Meguro-ku; locality: The University of Tokyo Campus, Komaba.; minimumElevationInMeters: 31; maximumElevationInMeters: 39; decimalLatitude: 35.66006; decimalLongitude: 139.68521; geodeticDatum: WGS84; **Identification:** identifiedBy: T. Ishikawa; dateIdentified: 2013; **Event:** samplingProtocol: net sweeping; eventDate: 2013-04-28; **Record Level:** institutionCode: KMUT; collectionCode: IC
- **Type status:** Other material. **Occurrence:** recordedBy: T. Ishikawa; individualCount: 2; sex: 1 male, 1 female; lifeStage: adult; otherCatalogNumbers: 2014-01451 | 2014-01452; **Taxon:** namePublishedIn: 1783; kingdom: Animalia; phylum: Arthropoda; class: Insecta; order: Hemiptera; family: Coreidae; genus: Homoeocerus; specificEpithet: unipunctatus; scientificNameAuthorship: Thunberg; **Location:** country: Japan; stateProvince: Tokyo; municipality: Meguro-ku; locality: The University of Tokyo Campus, Komaba.; minimumElevationInMeters: 31; maximumElevationInMeters: 39; decimalLatitude: 35.66006; decimalLongitude: 139.68521; geodeticDatum: WGS84; **Identification:** identifiedBy: T. Ishikawa; dateIdentified: 2013; **Event:** samplingProtocol: net sweeping; eventDate: 2013-05-12/2013-05-18; **Record Level:** institutionCode: KMUT; collectionCode: IC
- **Type status:** Other material. **Occurrence:** recordedBy: T. Ishikawa; individualCount: 1; sex: 1 female; lifeStage: adult; otherCatalogNumbers: 2014-01453; **Taxon:** namePublishedIn: 1783; kingdom: Animalia; phylum: Arthropoda; class: Insecta; order: Hemiptera; family: Coreidae; genus: Homoeocerus; specificEpithet: unipunctatus; scientificNameAuthorship: Thunberg; **Location:** country: Japan; stateProvince: Tokyo; municipality: Meguro-ku; locality: The University of Tokyo Campus, Komaba.; minimumElevationInMeters: 31; maximumElevationInMeters: 39; decimalLatitude: 35.66006; decimalLongitude: 139.68521; geodeticDatum: WGS84; **Identification:** identifiedBy: T. Ishikawa; dateIdentified: 2013; **Event:** samplingProtocol: net sweeping; eventDate: 2013-06-22; **Record Level:** institutionCode: KMUT; collectionCode: IC
- **Type status:** Other material. **Occurrence:** recordedBy: T. Ishikawa; individualCount: 1; sex: 1 female; lifeStage: adult; otherCatalogNumbers: 2014-01454; **Taxon:** namePublishedIn: 1783; kingdom: Animalia; phylum: Arthropoda; class: Insecta; order: Hemiptera; family: Coreidae; genus: Homoeocerus; specificEpithet: unipunctatus; scientificNameAuthorship: Thunberg; **Location:** country: Japan; stateProvince: Tokyo; municipality: Meguro-ku; locality: The University of Tokyo Campus, Komaba.; minimumElevationInMeters: 31; maximumElevationInMeters: 39; decimalLatitude: 35.66006; decimalLongitude: 139.68521; geodeticDatum: WGS84; **Identification:** identifiedBy: T. Ishikawa; dateIdentified: 2013; **Event:** samplingProtocol: net sweeping; eventDate: 2013-08-15; **Record Level:** institutionCode: KMUT; collectionCode: IC

#### Leptoglossus occidentalis

Heidemann, 1910

##### Materials

- **Type status:** Other material. **Occurrence:** recordedBy: T. Kato; individualCount: 1; sex: 1 female; lifeStage: adult; otherCatalogNumbers: 2014-01438; **Taxon:** namePublishedIn: 1910; kingdom: Animalia; phylum: Arthropoda; class: Insecta; order: Hemiptera; family: Coreidae; genus: Leptoglossus; specificEpithet: occidentalis; scientificNameAuthorship: Heidemann; **Location:** country: Japan; stateProvince: Tokyo; municipality: Meguro-ku; locality: The University of Tokyo Campus, Komaba.; minimumElevationInMeters: 31; maximumElevationInMeters: 39; decimalLatitude: 35.66006; decimalLongitude: 139.68521; geodeticDatum: WGS84; **Identification:** identifiedBy: T. Ishikawa; dateIdentified: 2013; **Event:** samplingProtocol: net sweeping; eventDate: 2013-10-28; **Record Level:** institutionCode: KMUT; collectionCode: IC

##### Notes

Known as a recent alien species to Japan (Tokyo) (Ishikawa and Kikuhara 2009).

#### Paradasynus spinosus

Hsiao, 1963

##### Materials

- **Type status:** Other material. **Occurrence:** recordedBy: T. Ishikawa; individualCount: 2; sex: 2 females; lifeStage: adult; otherCatalogNumbers: 2014-01439 | 2014-01440; **Taxon:** namePublishedIn: 1963; kingdom: Animalia; phylum: Arthropoda; class: Insecta; order: Hemiptera; family: Coreidae; genus: Paradasynus; specificEpithet: spinosus; scientificNameAuthorship: Hsiao; **Location:** country: Japan; stateProvince: Tokyo; municipality: Meguro-ku; locality: The University of Tokyo Campus, Komaba.; minimumElevationInMeters: 31; maximumElevationInMeters: 39; decimalLatitude: 35.66006; decimalLongitude: 139.68521; geodeticDatum: WGS84; **Identification:** identifiedBy: T. Ishikawa; dateIdentified: 2013; **Event:** samplingProtocol: net sweeping; eventDate: 2013-10-30; **Record Level:** institutionCode: KMUT; collectionCode: IC

#### Pentatomoidea

Leach, 1815

#### Plataspidae

Dallas, 1851

#### Megacopta punctatissima

(Montandon, 1896)

##### Materials

- **Type status:** Other material. **Occurrence:** recordedBy: T. Ishikawa; individualCount: 7; sex: 5 males, 2 females; lifeStage: adult; otherCatalogNumbers: 2014-01455 | 2014-01456 | 2014-01457 | 2014-01458 | 2014-01459 | 2014-01460 | 2014-01461; **Taxon:** namePublishedIn: 1896; kingdom: Animalia; phylum: Arthropoda; class: Insecta; order: Hemiptera; family: Plataspidae; genus: Megacopta; specificEpithet: punctatissima; scientificNameAuthorship: Montandon; **Location:** country: Japan; stateProvince: Tokyo; municipality: Meguro-ku; locality: The University of Tokyo Campus, Komaba.; minimumElevationInMeters: 31; maximumElevationInMeters: 39; decimalLatitude: 35.66006; decimalLongitude: 139.68521; geodeticDatum: WGS84; **Identification:** identifiedBy: T. Ishikawa; dateIdentified: 2013; **Event:** samplingProtocol: net sweeping; eventDate: 2013-04-28/2013-04-29; **Record Level:** institutionCode: KMUT; collectionCode: IC
- **Type status:** Other material. **Occurrence:** recordedBy: T. Ishikawa; individualCount: 3; sex: 1 male, 2 females; lifeStage: adult; otherCatalogNumbers: 2014-01462 | 2014-01463 | 2014-01464; **Taxon:** namePublishedIn: 1896; kingdom: Animalia; phylum: Arthropoda; class: Insecta; order: Hemiptera; family: Plataspidae; genus: Megacopta; specificEpithet: punctatissima; scientificNameAuthorship: Montandon; **Location:** country: Japan; stateProvince: Tokyo; municipality: Meguro-ku; locality: The University of Tokyo Campus, Komaba.; minimumElevationInMeters: 31; maximumElevationInMeters: 39; decimalLatitude: 35.66006; decimalLongitude: 139.68521; geodeticDatum: WGS84; **Identification:** identifiedBy: T. Ishikawa; dateIdentified: 2013; **Event:** samplingProtocol: net sweeping; eventDate: 2013-05-12/2013-05-18; **Record Level:** institutionCode: KMUT; collectionCode: IC
- **Type status:** Other material. **Occurrence:** recordedBy: T. Ishikawa; individualCount: 1; sex: 1 female; lifeStage: adult; otherCatalogNumbers: 2014-01465; **Taxon:** namePublishedIn: 1896; kingdom: Animalia; phylum: Arthropoda; class: Insecta; order: Hemiptera; family: Plataspidae; genus: Megacopta; specificEpithet: punctatissima; scientificNameAuthorship: Montandon; **Location:** country: Japan; stateProvince: Tokyo; municipality: Meguro-ku; locality: The University of Tokyo Campus, Komaba.; minimumElevationInMeters: 31; maximumElevationInMeters: 39; decimalLatitude: 35.66006; decimalLongitude: 139.68521; geodeticDatum: WGS84; **Identification:** identifiedBy: T. Ishikawa; dateIdentified: 2013; **Event:** samplingProtocol: net sweeping; eventDate: 2013-06-22; **Record Level:** institutionCode: KMUT; collectionCode: IC
- **Type status:** Other material. **Occurrence:** recordedBy: T. Ishikawa; individualCount: 1; sex: 1 male; lifeStage: adult; otherCatalogNumbers: 2014-01466; **Taxon:** namePublishedIn: 1896; kingdom: Animalia; phylum: Arthropoda; class: Insecta; order: Hemiptera; family: Plataspidae; genus: Megacopta; specificEpithet: punctatissima; scientificNameAuthorship: Montandon; **Location:** country: Japan; stateProvince: Tokyo; municipality: Meguro-ku; locality: The University of Tokyo Campus, Komaba.; minimumElevationInMeters: 31; maximumElevationInMeters: 39; decimalLatitude: 35.66006; decimalLongitude: 139.68521; geodeticDatum: WGS84; **Identification:** identifiedBy: T. Ishikawa; dateIdentified: 2013; **Event:** samplingProtocol: net sweeping; eventDate: 2013-10-30; **Record Level:** institutionCode: KMUT; collectionCode: IC

#### Cydnidae

Billberg, 1820

#### Adomerus triguttulus

(Motschulsky, 1866)

##### Materials

- **Type status:** Other material. **Occurrence:** recordedBy: K. Kishimoto-Yamada; individualCount: 5; sex: 3 males, 2 females; lifeStage: adult; otherCatalogNumbers: 2014-01477 | 2014-01478 | 2014-01479 | 2014-01480 | 2014-01481; **Taxon:** namePublishedIn: 1866; kingdom: Animalia; phylum: Arthropoda; class: Insecta; order: Hemiptera; family: Cydnidae; genus: Adomerus; specificEpithet: triguttulus; scientificNameAuthorship: Motschulsky; **Location:** country: Japan; stateProvince: Tokyo; municipality: Meguro-ku; locality: The University of Tokyo Campus, Komaba.; minimumElevationInMeters: 31; maximumElevationInMeters: 39; decimalLatitude: 35.66006; decimalLongitude: 139.68521; geodeticDatum: WGS84; **Identification:** identifiedBy: T. Ishikawa; dateIdentified: 2014; **Event:** samplingProtocol: net sweeping; eventDate: 2014-05-01; **Record Level:** institutionCode: KMUT; collectionCode: IC

#### Adrisa magna

(Uhler, 1860)

##### Materials

- **Type status:** Other material. **Occurrence:** recordedBy: T. Kato; individualCount: 1; sex: 1 male; lifeStage: adult; otherCatalogNumbers: 2014-01469; **Taxon:** namePublishedIn: 1860; kingdom: Animalia; phylum: Arthropoda; class: Insecta; order: Hemiptera; family: Cydnidae; genus: Adrisa; specificEpithet: magna; scientificNameAuthorship: Uhler; **Location:** country: Japan; stateProvince: Tokyo; municipality: Meguro-ku; locality: The University of Tokyo Campus, Komaba.; minimumElevationInMeters: 31; maximumElevationInMeters: 39; decimalLatitude: 35.66006; decimalLongitude: 139.68521; geodeticDatum: WGS84; **Identification:** identifiedBy: T. Ishikawa; dateIdentified: 2013; **Event:** samplingProtocol: net sweeping; eventDate: 2013-05-14; **Record Level:** institutionCode: KMUT; collectionCode: IC
- **Type status:** Other material. **Occurrence:** recordedBy: M. Saito; individualCount: 1; sex: 1 female; lifeStage: adult; otherCatalogNumbers: 2014-01470; **Taxon:** namePublishedIn: 1860; kingdom: Animalia; phylum: Arthropoda; class: Insecta; order: Hemiptera; family: Cydnidae; genus: Adrisa; specificEpithet: magna; scientificNameAuthorship: Uhler; **Location:** country: Japan; stateProvince: Tokyo; municipality: Meguro-ku; locality: The University of Tokyo Campus, Komaba.; minimumElevationInMeters: 31; maximumElevationInMeters: 39; decimalLatitude: 35.66006; decimalLongitude: 139.68521; geodeticDatum: WGS84; **Identification:** identifiedBy: T. Ishikawa; dateIdentified: 2014; **Event:** samplingProtocol: net sweeping; eventDate: 2014-05-01; **Record Level:** institutionCode: KMUT; collectionCode: IC

#### Chilocoris confusus

Horváth, 1919

##### Materials

- **Type status:** Other material. **Occurrence:** recordedBy: T. Ishikawa & K. Kishimoto-Yamada; individualCount: 2; sex: 2 males; lifeStage: adult; otherCatalogNumbers: 2014-01467 | 2014-01468; **Taxon:** namePublishedIn: 1919; kingdom: Animalia; phylum: Arthropoda; class: Insecta; order: Hemiptera; family: Cydnidae; genus: Chilocoris; specificEpithet: confusus; scientificNameAuthorship: Horváth; **Location:** country: Japan; stateProvince: Tokyo; municipality: Meguro-ku; locality: The University of Tokyo Campus, Komaba.; minimumElevationInMeters: 31; maximumElevationInMeters: 39; decimalLatitude: 35.66006; decimalLongitude: 139.68521; geodeticDatum: WGS84; **Identification:** identifiedBy: T. Ishikawa; dateIdentified: 2013; **Event:** samplingProtocol: Berlese funnel; eventDate: 2013-11-28; **Record Level:** institutionCode: KMUT; collectionCode: IC

##### Notes

First record in Tokyo.

#### Macroscytus japonensis

Scott, 1874

##### Materials

- **Type status:** Other material. **Occurrence:** recordedBy: T. Ishikawa; individualCount: 1; sex: 1 female; lifeStage: adult; otherCatalogNumbers: 2014-01471; **Taxon:** namePublishedIn: 1874; kingdom: Animalia; phylum: Arthropoda; class: Insecta; order: Hemiptera; family: Cydnidae; genus: Macroscytus; specificEpithet: japonensis; scientificNameAuthorship: Scott; **Location:** country: Japan; stateProvince: Tokyo; municipality: Meguro-ku; locality: The University of Tokyo Campus, Komaba.; minimumElevationInMeters: 31; maximumElevationInMeters: 39; decimalLatitude: 35.66006; decimalLongitude: 139.68521; geodeticDatum: WGS84; **Identification:** identifiedBy: T. Ishikawa; dateIdentified: 2013; **Event:** samplingProtocol: net sweeping; eventDate: 2013-05-04; **Record Level:** institutionCode: KMUT; collectionCode: IC
- **Type status:** Other material. **Occurrence:** recordedBy: T. Ishikawa; individualCount: 2; sex: 2 females; lifeStage: adult; otherCatalogNumbers: 2014-01472 | 2014-01473; **Taxon:** namePublishedIn: 1874; kingdom: Animalia; phylum: Arthropoda; class: Insecta; order: Hemiptera; family: Cydnidae; genus: Macroscytus; specificEpithet: japonensis; scientificNameAuthorship: Scott; **Location:** country: Japan; stateProvince: Tokyo; municipality: Meguro-ku; locality: The University of Tokyo Campus, Komaba.; minimumElevationInMeters: 31; maximumElevationInMeters: 39; decimalLatitude: 35.66006; decimalLongitude: 139.68521; geodeticDatum: WGS84; **Identification:** identifiedBy: T. Ishikawa; dateIdentified: 2013; **Event:** samplingProtocol: net sweeping; eventDate: 2013-06-17/2013-06-23; **Record Level:** institutionCode: KMUT; collectionCode: IC
- **Type status:** Other material. **Occurrence:** recordedBy: T. Ishikawa; individualCount: 1; sex: 1 female; lifeStage: adult; otherCatalogNumbers: 2014-01474; **Taxon:** namePublishedIn: 1874; kingdom: Animalia; phylum: Arthropoda; class: Insecta; order: Hemiptera; family: Cydnidae; genus: Macroscytus; specificEpithet: japonensis; scientificNameAuthorship: Scott; **Location:** country: Japan; stateProvince: Tokyo; municipality: Meguro-ku; locality: The University of Tokyo Campus, Komaba.; minimumElevationInMeters: 31; maximumElevationInMeters: 39; decimalLatitude: 35.66006; decimalLongitude: 139.68521; geodeticDatum: WGS84; **Identification:** identifiedBy: T. Ishikawa; dateIdentified: 2013; **Event:** samplingProtocol: net sweeping; eventDate: 2013-10-31; **Record Level:** institutionCode: KMUT; collectionCode: IC
- **Type status:** Other material. **Occurrence:** recordedBy: T. Ishikawa & K. Kishimoto-Yamada; individualCount: 1; sex: 1 female; lifeStage: adult; otherCatalogNumbers: 2014-01475; **Taxon:** namePublishedIn: 1874; kingdom: Animalia; phylum: Arthropoda; class: Insecta; order: Hemiptera; family: Cydnidae; genus: Macroscytus; specificEpithet: japonensis; scientificNameAuthorship: Scott; **Location:** country: Japan; stateProvince: Tokyo; municipality: Meguro-ku; locality: The University of Tokyo Campus, Komaba.; minimumElevationInMeters: 31; maximumElevationInMeters: 39; decimalLatitude: 35.66006; decimalLongitude: 139.68521; geodeticDatum: WGS84; **Identification:** identifiedBy: T. Ishikawa; dateIdentified: 2013; **Event:** samplingProtocol: Berlese funnel; eventDate: 2013-11-28; **Record Level:** institutionCode: KMUT; collectionCode: IC

#### Microporus nigrita

(Fabricius, 1794)

##### Materials

- **Type status:** Other material. **Occurrence:** recordedBy: T. Kato; individualCount: 1; sex: 1 male; lifeStage: adult; otherCatalogNumbers: 2014-01476; **Taxon:** namePublishedIn: 1794; kingdom: Animalia; phylum: Arthropoda; class: Insecta; order: Hemiptera; family: Cydnidae; genus: Microporus; specificEpithet: nigrita; scientificNameAuthorship: Fabricius; **Location:** country: Japan; stateProvince: Tokyo; municipality: Meguro-ku; locality: The University of Tokyo Campus, Komaba.; minimumElevationInMeters: 31; maximumElevationInMeters: 39; decimalLatitude: 35.66006; decimalLongitude: 139.68521; geodeticDatum: WGS84; **Identification:** identifiedBy: T. Ishikawa; dateIdentified: 2013; **Event:** samplingProtocol: net sweeping; eventDate: 2013-06-08; **Record Level:** institutionCode: KMUT; collectionCode: IC

#### Scutelleridae

Leach, 1815

#### Poecilocoris lewisi

(Distant, 1883)

##### Materials

- **Type status:** Other material. **Occurrence:** recordedBy: T. Ishikawa; individualCount: 1; sex: 1 female; lifeStage: adult; otherCatalogNumbers: 2014-01482; **Taxon:** namePublishedIn: 1883; kingdom: Animalia; phylum: Arthropoda; class: Insecta; order: Hemiptera; family: Scutelleridae; genus: Poecilocoris; specificEpithet: lewisi; scientificNameAuthorship: Distant; **Location:** country: Japan; stateProvince: Tokyo; municipality: Meguro-ku; locality: The University of Tokyo Campus, Komaba.; minimumElevationInMeters: 31; maximumElevationInMeters: 39; decimalLatitude: 35.66006; decimalLongitude: 139.68521; geodeticDatum: WGS84; **Identification:** identifiedBy: T. Ishikawa; dateIdentified: 2013; **Event:** samplingProtocol: net sweeping; eventDate: 2013-06-22; **Record Level:** institutionCode: KMUT; collectionCode: IC
- **Type status:** Other material. **Occurrence:** recordedBy: T. Ishikawa; individualCount: 1; sex: 1 male; lifeStage: adult; otherCatalogNumbers: 2014-01483; **Taxon:** namePublishedIn: 1883; kingdom: Animalia; phylum: Arthropoda; class: Insecta; order: Hemiptera; family: Scutelleridae; genus: Poecilocoris; specificEpithet: lewisi; scientificNameAuthorship: Distant; **Location:** country: Japan; stateProvince: Tokyo; municipality: Meguro-ku; locality: The University of Tokyo Campus, Komaba.; minimumElevationInMeters: 31; maximumElevationInMeters: 39; decimalLatitude: 35.66006; decimalLongitude: 139.68521; geodeticDatum: WGS84; **Identification:** identifiedBy: T. Ishikawa; dateIdentified: 2013; **Event:** samplingProtocol: net sweeping; eventDate: 2013-08-15; **Record Level:** institutionCode: KMUT; collectionCode: IC

#### Pentatomidae

Leach, 1815

#### Aelia fieberi

Scott, 1874

##### Materials

- **Type status:** Other material. **Occurrence:** recordedBy: T. Ishikawa; individualCount: 7; sex: 4 males, 3 females; lifeStage: adult; otherCatalogNumbers: 2014-01486 | 2014-01487 | 2014-01488 | 2014-01489 | 2014-01490 | 2014-01491 | 2014-01492; **Taxon:** namePublishedIn: 1874; kingdom: Animalia; phylum: Arthropoda; class: Insecta; order: Hemiptera; family: Pentatomidae; genus: Aelia; specificEpithet: fieberi; scientificNameAuthorship: Scott; **Location:** country: Japan; stateProvince: Tokyo; municipality: Meguro-ku; locality: The University of Tokyo Campus, Komaba.; minimumElevationInMeters: 31; maximumElevationInMeters: 39; decimalLatitude: 35.66006; decimalLongitude: 139.68521; geodeticDatum: WGS84; **Identification:** identifiedBy: T. Ishikawa; dateIdentified: 2013; **Event:** samplingProtocol: net sweeping; eventDate: 2013-05-12; **Record Level:** institutionCode: KMUT; collectionCode: IC
- **Type status:** Other material. **Occurrence:** recordedBy: T. Ishikawa; individualCount: 3; sex: 1 male, 2 females; lifeStage: adult; otherCatalogNumbers: 2014-01493 | 2014-01494 | 2014-01495; **Taxon:** namePublishedIn: 1874; kingdom: Animalia; phylum: Arthropoda; class: Insecta; order: Hemiptera; family: Pentatomidae; genus: Aelia; specificEpithet: fieberi; scientificNameAuthorship: Scott; **Location:** country: Japan; stateProvince: Tokyo; municipality: Meguro-ku; locality: The University of Tokyo Campus, Komaba.; minimumElevationInMeters: 31; maximumElevationInMeters: 39; decimalLatitude: 35.66006; decimalLongitude: 139.68521; geodeticDatum: WGS84; **Identification:** identifiedBy: T. Ishikawa; dateIdentified: 2013; **Event:** samplingProtocol: net sweeping; eventDate: 2013-05-19; **Record Level:** institutionCode: KMUT; collectionCode: IC

#### Dolycoris baccarum

(Linnaeus, 1758)

##### Materials

- **Type status:** Other material. **Occurrence:** recordedBy: T. Ishikawa; individualCount: 1; sex: 1 female; lifeStage: adult; otherCatalogNumbers: 2014-01524; **Taxon:** namePublishedIn: 1758; kingdom: Animalia; phylum: Arthropoda; class: Insecta; order: Hemiptera; family: Pentatomidae; genus: Dolycoris; specificEpithet: baccarum; scientificNameAuthorship: Linnaeus; **Location:** country: Japan; stateProvince: Tokyo; municipality: Meguro-ku; locality: The University of Tokyo Campus, Komaba.; minimumElevationInMeters: 31; maximumElevationInMeters: 39; decimalLatitude: 35.66006; decimalLongitude: 139.68521; geodeticDatum: WGS84; **Identification:** identifiedBy: T. Ishikawa; dateIdentified: 2013; **Event:** samplingProtocol: net sweeping; eventDate: 2013-06-23; **Record Level:** institutionCode: KMUT; collectionCode: IC
- **Type status:** Other material. **Occurrence:** recordedBy: T. Ishikawa; individualCount: 4; sex: 3 males, 1 female; lifeStage: adult; otherCatalogNumbers: 2014-01525 | 2014-01526 | 2014-01527 | 2014-01528; **Taxon:** namePublishedIn: 1758; kingdom: Animalia; phylum: Arthropoda; class: Insecta; order: Hemiptera; family: Pentatomidae; genus: Dolycoris; specificEpithet: baccarum; scientificNameAuthorship: Linnaeus; **Location:** country: Japan; stateProvince: Tokyo; municipality: Meguro-ku; locality: The University of Tokyo Campus, Komaba.; minimumElevationInMeters: 31; maximumElevationInMeters: 39; decimalLatitude: 35.66006; decimalLongitude: 139.68521; geodeticDatum: WGS84; **Identification:** identifiedBy: T. Ishikawa; dateIdentified: 2013; **Event:** samplingProtocol: net sweeping; eventDate: 2013-08-15; **Record Level:** institutionCode: KMUT; collectionCode: IC

#### Dybowskyia reticulata

(Dallas, 1851)

##### Materials

- **Type status:** Other material. **Occurrence:** recordedBy: T. Ishikawa; individualCount: 1; sex: 1 male; lifeStage: adult; otherCatalogNumbers: 2014-01529; **Taxon:** namePublishedIn: 1851; kingdom: Animalia; phylum: Arthropoda; class: Insecta; order: Hemiptera; family: Pentatomidae; genus: Dybowskyia; specificEpithet: reticulata; scientificNameAuthorship: Dallas; **Location:** country: Japan; stateProvince: Tokyo; municipality: Meguro-ku; locality: The University of Tokyo Campus, Komaba.; minimumElevationInMeters: 31; maximumElevationInMeters: 39; decimalLatitude: 35.66006; decimalLongitude: 139.68521; geodeticDatum: WGS84; **Identification:** identifiedBy: T. Ishikawa; dateIdentified: 2013; **Event:** samplingProtocol: net sweeping; eventDate: 2013-08-18; **Record Level:** institutionCode: KMUT; collectionCode: IC

#### Eysarcoris annamita

Breddin, 1909

##### Materials

- **Type status:** Other material. **Occurrence:** recordedBy: T. Ishikawa; individualCount: 1; sex: 1 male; lifeStage: adult; otherCatalogNumbers: 2014-01484; **Taxon:** namePublishedIn: 1909; kingdom: Animalia; phylum: Arthropoda; class: Insecta; order: Hemiptera; family: Pentatomidae; genus: Eysarcoris; specificEpithet: annamita; scientificNameAuthorship: Breddin; **Location:** country: Japan; stateProvince: Tokyo; municipality: Meguro-ku; locality: The University of Tokyo Campus, Komaba.; minimumElevationInMeters: 31; maximumElevationInMeters: 39; decimalLatitude: 35.66006; decimalLongitude: 139.68521; geodeticDatum: WGS84; **Identification:** identifiedBy: T. Ishikawa; dateIdentified: 2013; **Event:** samplingProtocol: net sweeping; eventDate: 2013-10-30; **Record Level:** institutionCode: KMUT; collectionCode: IC
- **Type status:** Other material. **Occurrence:** recordedBy: K. Kishimoto-Yamada; individualCount: 1; sex: 1 male; lifeStage: adult; otherCatalogNumbers: 2014-01485; **Taxon:** namePublishedIn: 1909; kingdom: Animalia; phylum: Arthropoda; class: Insecta; order: Hemiptera; family: Pentatomidae; genus: Eysarcoris; specificEpithet: annamita; scientificNameAuthorship: Breddin; **Location:** country: Japan; stateProvince: Tokyo; municipality: Meguro-ku; locality: The University of Tokyo Campus, Komaba.; minimumElevationInMeters: 31; maximumElevationInMeters: 39; decimalLatitude: 35.66006; decimalLongitude: 139.68521; geodeticDatum: WGS84; **Identification:** identifiedBy: T. Ishikawa; dateIdentified: 2014; **Event:** samplingProtocol: net sweeping; eventDate: 2014-05-01; **Record Level:** institutionCode: KMUT; collectionCode: IC

#### Glaucias subpunctatus

(Walker, 1867)

##### Materials

- **Type status:** Other material. **Occurrence:** recordedBy: T. Ishikawa; individualCount: 1; sex: 1 male; lifeStage: adult; otherCatalogNumbers: 2014-01519; **Taxon:** namePublishedIn: 1867; kingdom: Animalia; phylum: Arthropoda; class: Insecta; order: Hemiptera; family: Pentatomidae; genus: Glaucias; specificEpithet: subpunctatus; scientificNameAuthorship: Walker; **Location:** country: Japan; stateProvince: Tokyo; municipality: Meguro-ku; locality: The University of Tokyo Campus, Komaba.; minimumElevationInMeters: 31; maximumElevationInMeters: 39; decimalLatitude: 35.66006; decimalLongitude: 139.68521; geodeticDatum: WGS84; **Identification:** identifiedBy: T. Ishikawa; dateIdentified: 2013; **Event:** samplingProtocol: light trap; eventDate: 2013-08-02; **Record Level:** institutionCode: KMUT; collectionCode: IC
- **Type status:** Other material. **Occurrence:** recordedBy: T. Ishikawa; individualCount: 1; sex: 1 male; lifeStage: adult; otherCatalogNumbers: 2014-01520; **Taxon:** namePublishedIn: 1867; kingdom: Animalia; phylum: Arthropoda; class: Insecta; order: Hemiptera; family: Pentatomidae; genus: Glaucias; specificEpithet: subpunctatus; scientificNameAuthorship: Walker; **Location:** country: Japan; stateProvince: Tokyo; municipality: Meguro-ku; locality: The University of Tokyo Campus, Komaba.; minimumElevationInMeters: 31; maximumElevationInMeters: 39; decimalLatitude: 35.66006; decimalLongitude: 139.68521; geodeticDatum: WGS84; **Identification:** identifiedBy: T. Ishikawa; dateIdentified: 2013; **Event:** samplingProtocol: net sweeping; eventDate: 2013-10-21; **Record Level:** institutionCode: KMUT; collectionCode: IC
- **Type status:** Other material. **Occurrence:** recordedBy: T. Ishikawa; individualCount: 1; sex: 1 male; lifeStage: adult; otherCatalogNumbers: 2014-01521; **Taxon:** namePublishedIn: 1867; kingdom: Animalia; phylum: Arthropoda; class: Insecta; order: Hemiptera; family: Pentatomidae; genus: Glaucias; specificEpithet: subpunctatus; scientificNameAuthorship: Walker; **Location:** country: Japan; stateProvince: Tokyo; municipality: Meguro-ku; locality: The University of Tokyo Campus, Komaba.; minimumElevationInMeters: 31; maximumElevationInMeters: 39; decimalLatitude: 35.66006; decimalLongitude: 139.68521; geodeticDatum: WGS84; **Identification:** identifiedBy: T. Ishikawa; dateIdentified: 2013; **Event:** samplingProtocol: light trap; eventDate: 2013-10-28; **Record Level:** institutionCode: KMUT; collectionCode: IC
- **Type status:** Other material. **Occurrence:** recordedBy: T. Kato; individualCount: 1; sex: 1 female; lifeStage: adult; otherCatalogNumbers: 2014-01522; **Taxon:** namePublishedIn: 1867; kingdom: Animalia; phylum: Arthropoda; class: Insecta; order: Hemiptera; family: Pentatomidae; genus: Glaucias; specificEpithet: subpunctatus; scientificNameAuthorship: Walker; **Location:** country: Japan; stateProvince: Tokyo; municipality: Meguro-ku; locality: The University of Tokyo Campus, Komaba.; minimumElevationInMeters: 31; maximumElevationInMeters: 39; decimalLatitude: 35.66006; decimalLongitude: 139.68521; geodeticDatum: WGS84; **Identification:** identifiedBy: T. Ishikawa; dateIdentified: 2014; **Event:** samplingProtocol: light trap; eventDate: 2014-02-19; **Record Level:** institutionCode: KMUT; collectionCode: IC

#### Halyomorpha halys

(Stål, 1855)

##### Materials

- **Type status:** Other material. **Occurrence:** recordedBy: T. Ishikawa; individualCount: 1; sex: 1 male; lifeStage: adult; otherCatalogNumbers: 2014-01513; **Taxon:** namePublishedIn: 1855; kingdom: Animalia; phylum: Arthropoda; class: Insecta; order: Hemiptera; family: Pentatomidae; genus: Halyomorpha; specificEpithet: halys; scientificNameAuthorship: Stål; **Location:** country: Japan; stateProvince: Tokyo; municipality: Meguro-ku; locality: The University of Tokyo Campus, Komaba.; minimumElevationInMeters: 31; maximumElevationInMeters: 39; decimalLatitude: 35.66006; decimalLongitude: 139.68521; geodeticDatum: WGS84; **Identification:** identifiedBy: T. Ishikawa; dateIdentified: 2013; **Event:** samplingProtocol: light trap; eventDate: 2013-05-14; **Record Level:** institutionCode: KMUT; collectionCode: IC
- **Type status:** Other material. **Occurrence:** recordedBy: T. Ishikawa; individualCount: 2; sex: 1 male, 1 female; lifeStage: adult; otherCatalogNumbers: 2014-01514 | 2014-01515; **Taxon:** namePublishedIn: 1855; kingdom: Animalia; phylum: Arthropoda; class: Insecta; order: Hemiptera; family: Pentatomidae; genus: Halyomorpha; specificEpithet: halys; scientificNameAuthorship: Stål; **Location:** country: Japan; stateProvince: Tokyo; municipality: Meguro-ku; locality: The University of Tokyo Campus, Komaba.; minimumElevationInMeters: 31; maximumElevationInMeters: 39; decimalLatitude: 35.66006; decimalLongitude: 139.68521; geodeticDatum: WGS84; **Identification:** identifiedBy: T. Ishikawa; dateIdentified: 2013; **Event:** samplingProtocol: net sweeping; eventDate: 2013-05-19; **Record Level:** institutionCode: KMUT; collectionCode: IC
- **Type status:** Other material. **Occurrence:** recordedBy: T. Ishikawa; individualCount: 1; sex: 1 female; lifeStage: adult; otherCatalogNumbers: 2014-01516; **Taxon:** namePublishedIn: 1855; kingdom: Animalia; phylum: Arthropoda; class: Insecta; order: Hemiptera; family: Pentatomidae; genus: Halyomorpha; specificEpithet: halys; scientificNameAuthorship: Stål; **Location:** country: Japan; stateProvince: Tokyo; municipality: Meguro-ku; locality: The University of Tokyo Campus, Komaba.; minimumElevationInMeters: 31; maximumElevationInMeters: 39; decimalLatitude: 35.66006; decimalLongitude: 139.68521; geodeticDatum: WGS84; **Identification:** identifiedBy: T. Ishikawa; dateIdentified: 2013; **Event:** samplingProtocol: net sweeping; eventDate: 2013-06-18; **Record Level:** institutionCode: KMUT; collectionCode: IC
- **Type status:** Other material. **Occurrence:** recordedBy: T. Ishikawa; individualCount: 2; sex: 1 male, 1 female; lifeStage: adult; otherCatalogNumbers: 2014-01517 | 2014-01518; **Taxon:** namePublishedIn: 1855; kingdom: Animalia; phylum: Arthropoda; class: Insecta; order: Hemiptera; family: Pentatomidae; genus: Halyomorpha; specificEpithet: halys; scientificNameAuthorship: Stål; **Location:** country: Japan; stateProvince: Tokyo; municipality: Meguro-ku; locality: The University of Tokyo Campus, Komaba.; minimumElevationInMeters: 31; maximumElevationInMeters: 39; decimalLatitude: 35.66006; decimalLongitude: 139.68521; geodeticDatum: WGS84; **Identification:** identifiedBy: T. Ishikawa; dateIdentified: 2013; **Event:** samplingProtocol: light trap; eventDate: 2013-08-02; **Record Level:** institutionCode: KMUT; collectionCode: IC

#### Nezara viridula

(Linnaeus, 1758)

##### Materials

- **Type status:** Other material. **Occurrence:** recordedBy: T. Iwasaki; individualCount: 1; sex: 1 male; lifeStage: adult; otherCatalogNumbers: 2014-01523; **Taxon:** namePublishedIn: 1758; kingdom: Animalia; phylum: Arthropoda; class: Insecta; order: Hemiptera; family: Pentatomidae; genus: Nezara; specificEpithet: viridula; scientificNameAuthorship: Linnaeus; **Location:** country: Japan; stateProvince: Tokyo; municipality: Meguro-ku; locality: The University of Tokyo Campus, Komaba.; minimumElevationInMeters: 31; maximumElevationInMeters: 39; decimalLatitude: 35.66006; decimalLongitude: 139.68521; geodeticDatum: WGS84; **Identification:** identifiedBy: T. Ishikawa; dateIdentified: 2013; **Event:** samplingProtocol: net sweeping; eventDate: 2013-12-01; **Record Level:** institutionCode: KMUT; collectionCode: IC

##### Notes

First record in Tokyo.

#### Plautia stali

Scott, 1874

##### Materials

- **Type status:** Other material. **Occurrence:** recordedBy: T. Ishikawa; individualCount: 3; sex: 3 females; lifeStage: adult; otherCatalogNumbers: 2014-01496 | 2014-01497 | 2014-01498; **Taxon:** namePublishedIn: 1874; kingdom: Animalia; phylum: Arthropoda; class: Insecta; order: Hemiptera; family: Pentatomidae; genus: Plautia; specificEpithet: stali; scientificNameAuthorship: Scott; **Location:** country: Japan; stateProvince: Tokyo; municipality: Meguro-ku; locality: The University of Tokyo Campus, Komaba.; minimumElevationInMeters: 31; maximumElevationInMeters: 39; decimalLatitude: 35.66006; decimalLongitude: 139.68521; geodeticDatum: WGS84; **Identification:** identifiedBy: T. Ishikawa; dateIdentified: 2013; **Event:** samplingProtocol: net sweeping; eventDate: 2013-04-28/2013-05-01; **Record Level:** institutionCode: KMUT; collectionCode: IC
- **Type status:** Other material. **Occurrence:** recordedBy: T. Ishikawa; individualCount: 3; sex: 2 males, 1 female; lifeStage: adult; otherCatalogNumbers: 2014-01499 | 2014-01500 | 2014-01501; **Taxon:** namePublishedIn: 1874; kingdom: Animalia; phylum: Arthropoda; class: Insecta; order: Hemiptera; family: Pentatomidae; genus: Plautia; specificEpithet: stali; scientificNameAuthorship: Scott; **Location:** country: Japan; stateProvince: Tokyo; municipality: Meguro-ku; locality: The University of Tokyo Campus, Komaba.; minimumElevationInMeters: 31; maximumElevationInMeters: 39; decimalLatitude: 35.66006; decimalLongitude: 139.68521; geodeticDatum: WGS84; **Identification:** identifiedBy: T. Ishikawa; dateIdentified: 2013; **Event:** samplingProtocol: net sweeping; eventDate: 2013-05-12/2013-05-18; **Record Level:** institutionCode: KMUT; collectionCode: IC
- **Type status:** Other material. **Occurrence:** recordedBy: T. Ishikawa; individualCount: 1; sex: 1 female; lifeStage: adult; otherCatalogNumbers: 2014-01502; **Taxon:** namePublishedIn: 1874; kingdom: Animalia; phylum: Arthropoda; class: Insecta; order: Hemiptera; family: Pentatomidae; genus: Plautia; specificEpithet: stali; scientificNameAuthorship: Scott; **Location:** country: Japan; stateProvince: Tokyo; municipality: Meguro-ku; locality: The University of Tokyo Campus, Komaba.; minimumElevationInMeters: 31; maximumElevationInMeters: 39; decimalLatitude: 35.66006; decimalLongitude: 139.68521; geodeticDatum: WGS84; **Identification:** identifiedBy: T. Ishikawa; dateIdentified: 2013; **Event:** samplingProtocol: light trap; eventDate: 2013-05-23; **Record Level:** institutionCode: KMUT; collectionCode: IC
- **Type status:** Other material. **Occurrence:** recordedBy: T. Ishikawa; individualCount: 1; sex: 1 female; lifeStage: adult; otherCatalogNumbers: 2014-01503; **Taxon:** namePublishedIn: 1874; kingdom: Animalia; phylum: Arthropoda; class: Insecta; order: Hemiptera; family: Pentatomidae; genus: Plautia; specificEpithet: stali; scientificNameAuthorship: Scott; **Location:** country: Japan; stateProvince: Tokyo; municipality: Meguro-ku; locality: The University of Tokyo Campus, Komaba.; minimumElevationInMeters: 31; maximumElevationInMeters: 39; decimalLatitude: 35.66006; decimalLongitude: 139.68521; geodeticDatum: WGS84; **Identification:** identifiedBy: T. Ishikawa; dateIdentified: 2013; **Event:** samplingProtocol: net sweeping; eventDate: 2013-06-18; **Record Level:** institutionCode: KMUT; collectionCode: IC
- **Type status:** Other material. **Occurrence:** recordedBy: T. Ishikawa; individualCount: 5; sex: 5 females; lifeStage: adult; otherCatalogNumbers: 2014-01504 | 2014-01505 | 2014-01506 | 2014-01507 | 2014-01508; **Taxon:** namePublishedIn: 1874; kingdom: Animalia; phylum: Arthropoda; class: Insecta; order: Hemiptera; family: Pentatomidae; genus: Plautia; specificEpithet: stali; scientificNameAuthorship: Scott; **Location:** country: Japan; stateProvince: Tokyo; municipality: Meguro-ku; locality: The University of Tokyo Campus, Komaba.; minimumElevationInMeters: 31; maximumElevationInMeters: 39; decimalLatitude: 35.66006; decimalLongitude: 139.68521; geodeticDatum: WGS84; **Identification:** identifiedBy: T. Ishikawa; dateIdentified: 2013; **Event:** samplingProtocol: light trap; eventDate: 2013-08-02; **Record Level:** institutionCode: KMUT; collectionCode: IC
- **Type status:** Other material. **Occurrence:** recordedBy: T. Ishikawa; individualCount: 3; sex: 1 male, 2 females; lifeStage: adult; otherCatalogNumbers: 2014-01509 | 2014-01510 | 2014-01511; **Taxon:** namePublishedIn: 1874; kingdom: Animalia; phylum: Arthropoda; class: Insecta; order: Hemiptera; family: Pentatomidae; genus: Plautia; specificEpithet: stali; scientificNameAuthorship: Scott; **Location:** country: Japan; stateProvince: Tokyo; municipality: Meguro-ku; locality: The University of Tokyo Campus, Komaba.; minimumElevationInMeters: 31; maximumElevationInMeters: 39; decimalLatitude: 35.66006; decimalLongitude: 139.68521; geodeticDatum: WGS84; **Identification:** identifiedBy: T. Ishikawa; dateIdentified: 2013; **Event:** samplingProtocol: net sweeping; eventDate: 2013-10-30; **Record Level:** institutionCode: KMUT; collectionCode: IC
- **Type status:** Other material. **Occurrence:** recordedBy: T. Ishikawa & K. Kishimoto-Yamada; individualCount: 1; sex: 1 male; lifeStage: adult; otherCatalogNumbers: 2014-01512; **Taxon:** namePublishedIn: 1874; kingdom: Animalia; phylum: Arthropoda; class: Insecta; order: Hemiptera; family: Pentatomidae; genus: Plautia; specificEpithet: stali; scientificNameAuthorship: Scott; **Location:** country: Japan; stateProvince: Tokyo; municipality: Meguro-ku; locality: The University of Tokyo Campus, Komaba.; minimumElevationInMeters: 31; maximumElevationInMeters: 39; decimalLatitude: 35.66006; decimalLongitude: 139.68521; geodeticDatum: WGS84; **Identification:** identifiedBy: T. Ishikawa; dateIdentified: 2013; **Event:** samplingProtocol: Berlese funnel; eventDate: 2013-11-28; **Record Level:** institutionCode: KMUT; collectionCode: IC

#### Acanthosomatidae

Signoret, 1864

#### Acanthosoma denticaudum

Jakovlev, 1880

##### Materials

- **Type status:** Other material. **Occurrence:** recordedBy: T. Ishikawa; individualCount: 1; sex: 1 male; lifeStage: adult; otherCatalogNumbers: 2014-01530; **Taxon:** namePublishedIn: 1880; kingdom: Animalia; phylum: Arthropoda; class: Insecta; order: Hemiptera; family: Acanthosomatidae; genus: Acanthosoma; specificEpithet: denticaudum; scientificNameAuthorship: Jakovlev; **Location:** country: Japan; stateProvince: Tokyo; municipality: Meguro-ku; locality: The University of Tokyo Campus, Komaba.; minimumElevationInMeters: 31; maximumElevationInMeters: 39; decimalLatitude: 35.66006; decimalLongitude: 139.68521; geodeticDatum: WGS84; **Identification:** identifiedBy: T. Ishikawa; dateIdentified: 2013; **Event:** samplingProtocol: net sweeping; eventDate: 2013-08-15; **Record Level:** institutionCode: KMUT; collectionCode: IC

#### Acanthosoma giganteum

Matsumura, 1913

##### Materials

- **Type status:** Other material. **Occurrence:** recordedBy: T. Ishikawa; individualCount: 1; sex: 1 male; lifeStage: adult; otherCatalogNumbers: 2014-01531; **Taxon:** namePublishedIn: 1913; kingdom: Animalia; phylum: Arthropoda; class: Insecta; order: Hemiptera; family: Acanthosomatidae; genus: Acanthosoma; specificEpithet: giganteum; scientificNameAuthorship: Matsumura; **Location:** country: Japan; stateProvince: Tokyo; municipality: Meguro-ku; locality: The University of Tokyo Campus, Komaba.; minimumElevationInMeters: 31; maximumElevationInMeters: 39; decimalLatitude: 35.66006; decimalLongitude: 139.68521; geodeticDatum: WGS84; **Identification:** identifiedBy: T. Ishikawa; dateIdentified: 2013; **Event:** samplingProtocol: net sweeping; eventDate: 2013-10-30; **Record Level:** institutionCode: KMUT; collectionCode: IC

#### Sastragala esakii

Hasegawa, 1959

##### Materials

- **Type status:** Other material. **Occurrence:** recordedBy: T. Ishikawa; individualCount: 1; sex: 1 female; lifeStage: adult; otherCatalogNumbers: 2014-01532; **Taxon:** namePublishedIn: 1959; kingdom: Animalia; phylum: Arthropoda; class: Insecta; order: Hemiptera; family: Acanthosomatidae; genus: Sastragala; specificEpithet: esakii; scientificNameAuthorship: Hasegawa; **Location:** country: Japan; stateProvince: Tokyo; municipality: Meguro-ku; locality: The University of Tokyo Campus, Komaba.; minimumElevationInMeters: 31; maximumElevationInMeters: 39; decimalLatitude: 35.66006; decimalLongitude: 139.68521; geodeticDatum: WGS84; **Identification:** identifiedBy: T. Ishikawa; dateIdentified: 2013; **Event:** samplingProtocol: net sweeping; eventDate: 2013-10-30; **Record Level:** institutionCode: KMUT; collectionCode: IC

## Analysis

**Figure 7.**
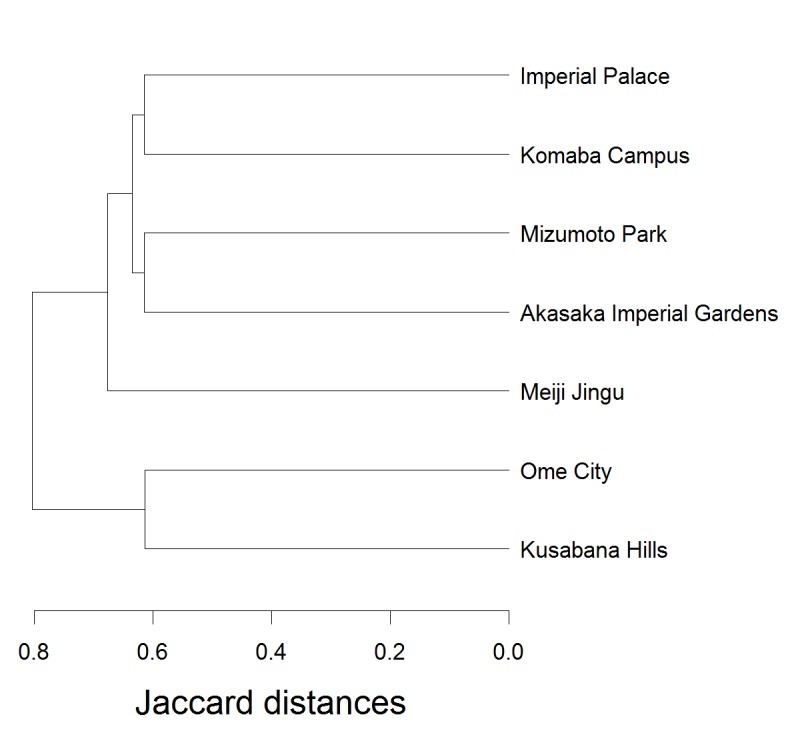
Cluster analysis of Heteroptera assemblages in Komaba Campus and the six reference sites based on Jaccard distances.

Cluster analysis based on Jaccard distances revealed two major assemblage groups; one consisted of highly to moderately urbanized localities (Meiji Jingu, Akasaka Imperial Gardens, Imperial Palace, Mizumoto Park, and the campus) and the other of suburbanized localities (Kusabana Hills and Ome City) (Fig. 7).

## Discussion

In our qualitative survey, we recorded 115 species of Heteroptera on the Komaba Campus of the University of Tokyo (Table 2). The species richness at campus locations tends to be higher than that of other references sites (Table 1), even though the campus is situated in an urban area within the center of Tokyo and has the smallest area of all sites. The rich campus vegetation presumably derived from effective landscaping managements. These activities may have enhanced heteropteran species diversity. It is, however, possible that the surveys for the majority of reference sites were insufficient, both in terms of quantity and quality, resulting in the relatively low documented species richness. Faunal surveys are often affected by biases related to season, research frequency, collection method, and sampling effort (Tomokuni 2005). More intensive surveys may reveal similar species richness to that of the campus, even in green spaces in highly urbanized zones.

Cluster analysis of assemblages revealed two major groups (Fig. 7). This indicates that the heteropteran fauna detected on the campus was more similar to those of highly urbanized localities than to those of suburbanized localities. However, the analysis also indicated differences in species composition among the five urbanized localities, including the campus (Fig. 7), irrespective of the distance between the respective urbanized localities, even for the closest two locations, the campus and Meiji Jingu. The differences might reflect that each of the green spaces has a peculiar ecosystem in terms of the heteropteran fauna. However, it is also necessary to consider the relative sufficiency of the surveys for accurately evaluating the urban faunae and ecosystems. Further surveys will clarify the attributes of the urban fauna and biodiversity, and suggest appropriate, sustainable urbanization, or exploitation.

## Figures and Tables

**Table 1.** Detailed characteristics of each reference site. All sites are situated in Tokyo (see Fig. 1).

Locality	Site area (ha)	Environment aspect	Number of species	Reference (for number of species)
Meiji Jingu	70	highly urbanized	83	Ishikawa and Hayashi (2013)
Akasaka Imperial Gardens	51	highly urbanized	80	Tomokuni (2005), Tomokuni (2006)
Imperial Palace	115	highly urbanized	133	Tomokuni et al. (2000), Tomokuni (2006)
Mizumoto Park	94	moderately urbanized	96	Tago (2006)
Kusabana Hills	2200	suburbanized	81	Kubota (1995)
Ome City	10000	suburbanized	90	Ome Municipal Museum of Provincial History (1982)

**Table 2.** List of species collected by net sweeping, light traps, and Tullgren funnels in Komaba Campus of the University of Tokyo, Tokyo, Japan.

Family	Species	Net sweeping	Light trap	Tullgren funnel
Enicocephalidae	*Hoplitocoris lewisi* (Distant, 1903)	3	0	10
Enicocephalidae	*Stenopirates japonicus* (Esaki, 1935)	0	0	1
Corixidae	*Micronecta orientalis* Wróblewski, 1960	1	0	0
Notonectidae	*Anisops ogasawarensis* Matsumura, 1915	5	0	0
Hydrometridae	*Hydrometra procera* Horváth, 1905	0	1	0
Veliidae	*Microvelia douglasi* Scott, 1874	106	0	0
Veliidae	*Microvelia horvathi* Lundblad, 1933	6	0	0
Gerridae	*Aquarius elongatus* (Uhler, 1897)	1	0	0
Gerridae	*Aquarius paludum paludum* (Fabricius, 1794)	3	0	0
Gerridae	*Gerris latiabdominis* Miyamoto, 1958	12	0	0
Saldidae	*Saldula saltatoria* (Linnaeus, 1758)	1	0	0
Tingidae	*Corythucha ciliata* (Say, 1832)	28	0	0
Tingidae	*Corythucha marmorata* (Uhler, 1878)	45	0	0
Tingidae	*Cysteochila consueta* Drake, 1948	36	0	0
Tingidae	*Dulinius conchatus* Distant, 1903	16	0	0
Tingidae	*Stephanitis nashi* Esaki et Takeya, 1931	6	0	0
Tingidae	*Stephanitis pyrioides* (Scott, 1874)	2	0	0
Tingidae	*Stephanitis svensoni* Drake, 1912	1	0	0
Tingidae	*Stephanitis takeyai* Drake et Maa, 1955	1	0	0
Tingidae	*Uhlerites debilis* (Uhler, 1896)	5	0	0
Miridae	*Apolygus hilaris* (Horváth, 1905)	47	0	0
Miridae	*Apolygus spinolae* (Meyer-Dür, 1841)	1	0	0
Miridae	*Apolygus subpulchellus* (Kerzhner, 1988)	134	0	0
Miridae	*Atractotomoidea castanea* Yasunaga, 1999	1	0	0
Miridae	*Campylomma lividum* Reuter, 1885	6	0	0
Miridae	*Campyloneura virgula* (Herrich-Schaeffer, 1836)	9	0	0
Miridae	*Castanopsides hasegawai* Yasunaga, 1992	2	1	0
Miridae	*Charagochilus angusticollis* Linnavuori, 1961	24	0	0
Miridae	*Cimidaeorus hasegawai* Nakatani, Yasunaga et Takai, 2000	1	0	0
Miridae	*Coridromius chinensis* Liu et Zhao, 1999	1	0	0
Miridae	*Creontiades coloripes* Hsiao, 1963	3	0	0
Miridae	*Dryophilocoris miyamotoi* Yasunaga, 1999	3	0	0
Miridae	*Eurystylus coelestialium* (Kirkaldy, 1902)	13	0	0
Miridae	*Eurystylus luteus* Hsiao, 1941	5	0	0
Miridae	*Harpocera orientalis* Kerzhner, 1979	1	0	0
Miridae	*Kasumiphylus kyushuensis* (Linnavuori, 1961)	1	7	0
Miridae	*Monalocoris filicis* (Linnaeus, 1758)	44	0	0
Miridae	*Neolygus pteleinus* (Kerzhner, 1977)	10	1	0
Miridae	*Philostephanus rubripes* (Jakovlev, 1876)	1	0	0
Miridae	*Phylus miyamotoi* Yasunaga, 1999	12	0	0
Miridae	*Pilophorus setulosus* Horváth, 1905	16	0	0
Miridae	*Pilophorus typicus* (Dsitant, 1909)	6	1	0
Miridae	*Psallus bagjonicus* Josifov, 1983	9	0	0
Miridae	*Psallus edoensis* Yasunaga et Vinokurov, 2000	36	0	0
Miridae	*Psallus roseoguttatus* Yasunaga et Vinokurov, 2000	8	0	0
Miridae	*Pseudoloxops miyamotoi* Yasunaga, 1997	1	0	0
Miridae	*Pseudophylus flavipes* (Nitobe, 1906)	43	0	0
Miridae	*Sejanus komabanus* Yasunaga, Ishikawa et Ito, 2013	7	0	0
Miridae	*Stethoconus japonicus* Schumacher, 1917	1	2	0
Miridae	*Taylorilygus apicalis* (Fieber, 1861)	15	0	0
Miridae	*Termatophylum hikosanum* Miyamoto, 1965	2	0	0
Miridae	*Trigonotylus caelestialium* (Kirkaldy, 1902)	47	3	0
Miridae	*Yamatolygus* sp.	1	0	0
Miridae	*Zanchius tarasovi* Kerzhner, 1988	16	0	0
Nabidae	*Nabis kinbergii* Reuter, 1872	11	0	0
Anthocoridae	*Amphiareus obscuriceps* (Poppius, 1909)	16	12	0
Anthocoridae	*Cardiastethus exiguus* Poppius, 1913	3	4	0
Anthocoridae	*Orius minutus* (Linnaeus, 1758)	112	1	0
Anthocoridae	*Orius nagaii* Yasunaga, 1993	1	0	0
Anthocoridae	*Orius sauteri* (Poppius, 1909)	14	0	0
Anthocoridae	*Physopleurella armata* Poppius, 1909	0	23	0
Reduviidae	*Empicoris minutus* Usinger, 1946	10	0	0
Reduviidae	*Haematoloecha nigrorufa* (Stål, 1867)	1	0	0
Reduviidae	*Velinus nodipes* (Uhler, 1860)	3	0	0
Pachygronthidae	*Pachygrontha antennata* (Uhler, 1860)	27	0	0
Pachygronthidae	*Pachygrontha similis* Uhler, 1896	2	0	0
Rhyparochromidae	*Botocudo japonicus* (Hidaka, 1959)	0	1	0
Rhyparochromidae	*Gyndes pallicornis* (Dallas, 1852)	9	1	0
Rhyparochromidae	*Metochus abbreviatus* Scott, 1874	1	2	0
Rhyparochromidae	*Neolethaeus dallasi* (Scott, 1874)	1	0	0
Rhyparochromidae	*Pamerana scotti* (Distant, 1901)	2	1	0
Rhyparochromidae	*Panaorus japonicus* (Stål, 1874)	1	0	0
Rhyparochromidae	*Stigmatonotum geniculatum* (Motschulsky, 1863)	0	1	0
Rhyparochromidae	*Togo hemipterus* (Scott, 1874)	16	0	0
Geocoridae	*Geocoris proteus* Distant, 1883	20	0	0
Geocoridae	*Geocoris varius* (Uhler, 1860)	1	0	0
Blissidae	*Dimorphopterus bicoloripes* (Distant, 1883)	45	0	0
Lygaeidae	*Nysius plebeius* Distant, 1883	15	0	0
Lygaeidae	*Nysius* sp.	175	0	0
Malcidae	*Chauliops fallax* Scott, 1874	29	0	0
Berytidae	*Metacanthus pulchellus* Dallas, 1852	3	0	0
Berytidae	*Yemma exilis* Horváth, 1905	18	1	0
Largidae	*Physopelta gutta* (Burmeister, 1834)	3	0	0
Largidae	*Physopelta parviceps* Blöte, 1931	2	0	0
Pyrrhocoridae	*Pyrrhocoris sibiricus* Kuschakewitsch, 1866	2	0	0
Alydidae	*Leptocorisa chinensis* Dallas, 1852	4	0	0
Alydidae	*Paraplesius vulgaris* (Hsiao, 1964)	3	0	0
Alydidae	*Riptortus pedestris* (Fabricius, 1775)	2	0	0
Rhopalidae	*Liorhyssus hyalinus* (Fabricius, 1794)	7	0	0
Rhopalidae	*Rhopalus maculatus* (Fieber, 1837)	1	0	0
Rhopalidae	*Stictopleurus punctatonervosus* (Goeze, 1778)	4	0	0
Coreidae	*Acanthocoris sordidus* (Thunberg, 1783)	3	0	0
Coreidae	*Cletus punctiger* (Dallas, 1852)	1	0	0
Coreidae	*Cletus schmidti* Kiritshenko, 1916	8	0	0
Coreidae	*Homoeocerus unipunctatus* (Thunberg, 1783)	5	0	0
Coreidae	*Leptoglossus occidentalis* Heidemann, 1910	1	0	0
Coreidae	*Paradasynus spinosus* Hsiao, 1963	2	0	0
Plataspidae	*Megacopta punctatissima* (Montandon, 1896)	12	0	0
Cydnidae	*Adomerus triguttulus* (Motschulsky, 1866)	5	0	0
Cydnidae	*Adrisa magna* (Uhler, 1860)	2	0	0
Cydnidae	*Chilocoris confusus* Horváth, 1919	0	0	2
Cydnidae	*Macroscytus japonensis* Scott, 1874	4	0	1
Cydnidae	*Microporus nigrita* (Fabricius, 1794)	1	0	0
Scutelleridae	*Poecilocoris lewisi* (Distant, 1883)	2	0	0
Pentatomidae	*Aelia fieberi* Scott, 1874	10	0	0
Pentatomidae	*Dolycoris baccarum* (Linnaeus, 1758)	5	0	0
Pentatomidae	*Dybowskyia reticulata* (Dallas, 1851)	1	0	0
Pentatomidae	*Eysarcoris annamita* Breddin, 1909	2	0	0
Pentatomidae	*Glaucias subpunctatus* (Walker, 1867)	1	3	0
Pentatomidae	*Halyomorpha halys* (Stål, 1855)	3	3	0
Pentatomidae	*Nezara viridula* (Linnaeus, 1758)	1	0	0
Pentatomidae	*Plautia stali* Scott, 1874	10	6	1
Acanthosomatidae	*Acanthosoma denticaudum* Jakovlev, 1880	1	0	0
Acanthosomatidae	*Acanthosoma giganteum* Matsumura, 1913	1	0	0
Acanthosomatidae	*Sastragala esakii* Hasegawa, 1959	1	0	0
